# Discovery and In Vivo Proof of Concept of a Highly Potent Dual Inhibitor of Soluble Epoxide Hydrolase and Acetylcholinesterase for the Treatment of Alzheimer’s Disease

**DOI:** 10.1021/acs.jmedchem.1c02150

**Published:** 2022-03-10

**Authors:** Sandra Codony, Caterina Pont, Christian Griñán-Ferré, Ania Di Pede-Mattatelli, Carla Calvó-Tusell, Ferran Feixas, Sílvia Osuna, Júlia Jarné-Ferrer, Marina Naldi, Manuela Bartolini, María Isabel Loza, José Brea, Belén Pérez, Clara Bartra, Coral Sanfeliu, Jordi Juárez-Jiménez, Christophe Morisseau, Bruce D. Hammock, Mercè Pallàs, Santiago Vázquez, Diego Muñoz-Torrero

**Affiliations:** † Laboratory of Medicinal Chemistry (CSIC Associated Unit), Faculty of Pharmacy and Food Sciences, and Institute of Biomedicine (IBUB), University of Barcelona (UB), Av. Joan XXIII 27-31, E-08028 Barcelona, Spain; ‡ Pharmacology Section, Department of Pharmacology, Toxicology and Therapeutic Chemistry, Faculty of Pharmacy and Food Sciences, and Institute of Neurosciences, University of Barcelona (UB), Av. Joan XXIII 27-31, E-08028 Barcelona, Spain; § Department of Pharmacy and Pharmaceutical Technology and Physical Chemistry, Faculty of Pharmacy and Food Sciences, and Institute of Theoretical and Computational Chemistry (IQTCUB), University of Barcelona (UB), Av. Joan XXIII 27-31, E-08028 Barcelona, Spain; ∥ CompBioLab Group, Departament de Química and Institut de Química Computacional i Catàlisi (IQCC), Universitat de Girona, C/ Maria Aurèlia Capmany 69, E-17003 Girona, Spain; ⊥ Institució Catalana de Recerca i Estudis Avançats (ICREA), E-08010 Barcelona, Spain; # Department of Pharmacy and Biotechnology, University of Bologna, Via Belmeloro, 6, I-40126 Bologna, Italy; ∇ BioFarma Research Group, Centro Singular de Investigación en Medicina Molecular y Enfermedades Crónicas (CIMUS), Universidade de Santiago de Compostela, Av. de Barcelona s/n, E-15782 Santiago de Compostela, Spain; ○ Department of Pharmacology, Therapeutics and Toxicology, Autonomous University of Barcelona, E-08193 Bellaterra, Spain; ◆ Institute of Biomedical Research of Barcelona, CSIC and Institut d’Investigacions Biomèdiques August Pi i Sunyer (IDIBAPS), Rosselló, 149, E-08036 Barcelona, Spain; ¶ Department of Entomology and Nematology and Comprehensive Cancer Center, University of California, One Shields Avenue, Davis, California 95616, United States

## Abstract

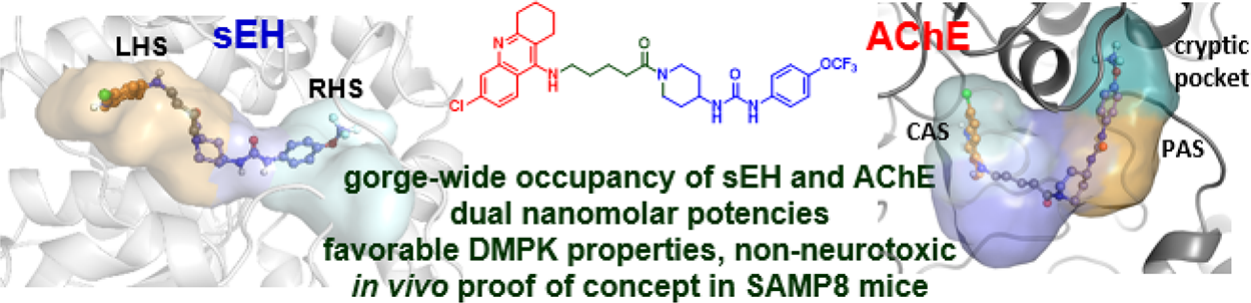

With innumerable clinical failures of target-specific drug candidates for multifactorial diseases, such as Alzheimer’s disease (AD), which remains inefficiently treated, the advent of multitarget drug discovery has brought a new breath of hope. Here, we disclose a class of 6-chlorotacrine (huprine)–TPPU hybrids as dual inhibitors of the enzymes soluble epoxide hydrolase (sEH) and acetylcholinesterase (AChE), a multitarget profile to provide cumulative effects against neuroinflammation and memory impairment. Computational studies confirmed the gorge-wide occupancy of both enzymes, from the main site to a secondary site, including a so far non-described AChE cryptic pocket. The lead compound displayed in vitro dual nanomolar potencies, adequate brain permeability, aqueous solubility, human microsomal stability, lack of neurotoxicity, and it rescued memory, synaptic plasticity, and neuroinflammation in an AD mouse model, after low dose chronic oral administration.

## Introduction

The clinical development of drugs against complex multifactorial diseases is undermined by high attrition rates, mainly due to the lack of efficacy of candidates that were designed to hit with high potency and selectivity a specific biological target.^1^ Severe diseases, such as cancer and neurodegenerative diseases, have a multifactorial etiology as they result from the dysregulation of multiple signaling pathways, so they should be more efficiently tackled by the simultaneous modulation of multiple targets. This has made the development of multitarget compounds, i.e., single molecules that modulate multiple biological targets, one of the most intensive research areas.^2−5^ Not only are multitarget compounds more likely to be more effective than single-target drugs, but they also should benefit from a much simpler and less expensive development, fewer side effects, no risk of drug–drug interactions, and improved patient compliance, relative to the classical strategy for modulating multiple targets, based on drug combinations.^6^

Alzheimer’s disease (AD), the most prevalent neurodegenerative disease, is one of such multifactorial diseases that lack an effective treatment, which would greatly benefit from the development of multitarget drugs.^7−19^ A key step of multitarget drug design is the selection of the targets, whose modulation should result in additive or synergistic effects, preferably on key pathogenic mechanisms.^3^

Brain inflammation is one of the main mechanisms underlying AD progression.^20^ Neuroinflammation is triggered by high concentrations of pro-inflammatory cytokines released by activated microglia and astrocytes, eventually leading to neuronal damage. Some metabolites of arachidonic acid, namely, the epoxyeicosatrienoic acids (EETs), reduce inflammation and attenuate oxidative stress, among other effects.^21^ EETs are metabolized by epoxide ring opening to the corresponding diols by the soluble epoxide hydrolase (sEH), upregulated in AD patients,^22,23^ which terminates the beneficial effects of EETs. Brain sEH was recently validated as a novel target of interest for AD treatment.^22−25^ sEH inhibitors have shown beneficial effects in two different mouse models of AD, SAMP8 and 5×FAD mice, in which they rescued cognitive impairment and reduced neuroinflammation, and other key pathological hallmarks (tau hyperphosphorylation and amyloid burden).^22^

A marked cholinergic deficit in the central nervous system (CNS) of AD patients mainly accounts for cognitive impairment. Acetylcholinesterase (AChE) hydrolyzes the neurotransmitter acetylcholine (ACh), thereby terminating cholinergic signaling. Indeed, inhibition of brain AChE is a well-established mechanism of action of anti-AD drugs, with three out of the four currently marketed drugs being AChE inhibitors.^26^ Strikingly, the increased levels of ACh that result from AChE inhibition may promote arachidonic acid metabolism to EETs, upon activation of ACh muscarinic M1 receptors,^27^ thereby potentiating the anti-neuroinflammatory effects of EETs.

Thus, we inferred that the so far unexplored dual targeting of sEH and AChE might open a new avenue for AD treatment as it should result in cumulative effects for reducing neuroinflammation and preventing cognitive impairment, i.e., disease-modifying plus symptomatic effects. As a proof of concept, here, we report the design, synthesis, in vitro biological activities, neurotoxicity and DMPK properties, molecular modeling, and an in vivo efficacy study in a mouse model of AD of a class of dual inhibitors of sEH and AChE.

## Results and Discussion

### Design and Synthesis of the Dual sEH/AChE Inhibitors

**Figure 1:**
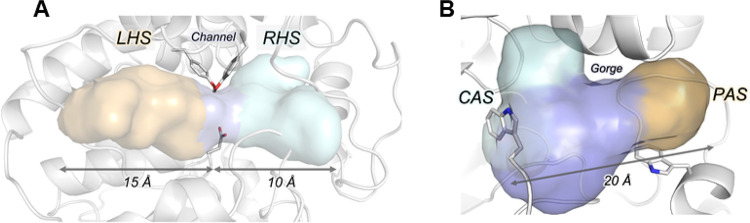
Active site cavities of sEH (A) and AChE (B).

Apart from conditioning therapeutic efficacy, the selected targets to be hit by a multitarget drug also drive its design, which is usually performed by joining with a linker, merging or overlapping in part two or more pharmacophores. To hit targets with binding regions that are buried deep inside the protein, the most useful design approach is the linked-pharmacophore strategy.^4^ Such proteins contain the main binding site at the end of a cavity and a secondary or peripheral site at the cavity entrance. In those cases, one scaffold is selected for each target to interact with the main binding site, whereas the second scaffold might interact with the peripheral site, provided that the linker that joins the two moieties affords the appropriate geometry and distance. The resulting dual-site binding usually leads to increased potencies as an added benefit apart from modulating two different targets. Both sEH and AChE belong to this type of protein. The active site of sEH is buried inside the protein core in an L-shaped pocket, with two branches of 15 and 10 Å, named left-hand side (LHS) and right-hand side (RHS), respectively, connected by a small bottleneck, so that the total length of the sEH active site is up to 25 Å (Figure 1A).^28,29^ The catalytic site (catalytic anionic site or CAS) of AChE is buried at the bottom of a narrow gorge of 20 Å depth, at whose entrance is located the peripheral anionic site (PAS) (Figure 1B).^30^

**Figure 2:**
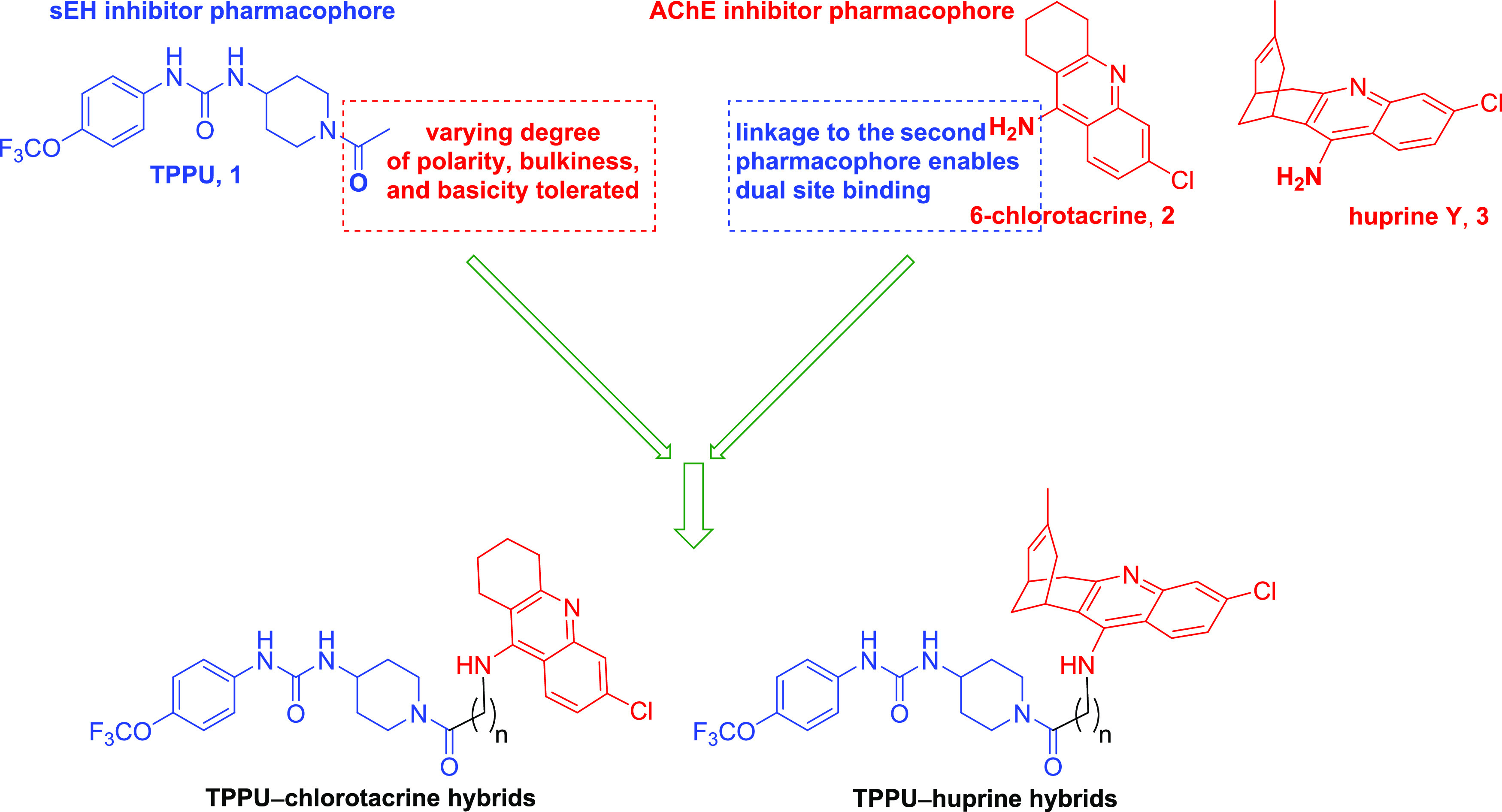
Design of the dual sEH/AChE inhibitors by the linked-pharmacophore strategy.

Thus, dual inhibitors were designed by linking the scaffolds of TPPU (**1**), a potent sEH inhibitor,^31^ and 6-chlorotacrine (**2**), a potent inhibitor of AChE,^32^ through a short oligomethylene tether (two to four methylenes), which was deemed adequate to enable the hybrids to span the active site cavities of both enzymes (Figure 2).

The linkage point at the TPPU moiety was the propanoyl group since it is known that away from that point fragments of varied polarity, bulkiness and basicity are tolerated within the LHS of sEH,^33^ which might enable a good fit of the alkylenechlorotacrine moiety of the hybrids. In the case of the 6-chlorotacrine scaffold, the primary amino group is known to be an appropriate point of attachment to enable the linker-second pharmacophore fragment to span the gorge up to the PAS,^34^ where the benzene ring of the TPPU moiety of the hybrids might establish π–π stacking interactions with the indol ring of the characteristic PAS Trp286 residue. The structure of the potent anticholinesterase compound huprine Y,^35^ closely related to 6-chlorotacrine but chiral, was also used in both enantiomeric forms, (−)-(7*S*,11*S*) and (+)-(7*R*,11*R*), as the AChE inhibitor pharmacophore in some hybrids to further explore potential enantioselective interactions at both targets.

**Scheme 1:**
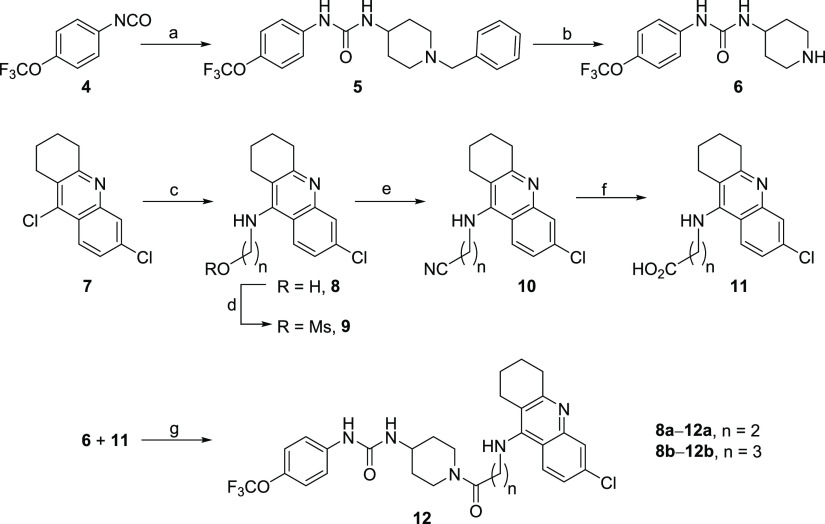
Synthesis of Hybrids **12a** and **12b** Reagents and conditions: (a) 4-amino-1-benzylpiperidine, DCM, RT, overnight, quantitative; (b) H_2_, 1 atm, 10% Pd/C, conc HCl, MeOH, 5 days, 82%; (c) 2-aminoethanol or 3-aminopropanol, 1-pentanol, reflux, overnight, 93% (**8a**), 71% (**8b**); (d) MsCl, Et_3_N, DCM, −10 °C, 30 min, quantitative; (e) NaCN, DMF, 35 °C, 2 h, 51% (**10a**), 91% (**10b**); (f) 5 N HCl, reflux, 3 h; (g) **11**, EDC·HCl, HOBt, Et_3_N, 10:1 EtOAc/DMF, 10 min, RT, then **6**·HCl, RT, overnight, 44% (**12a**, over two steps from **10a**), 61% (**12b**, over two steps from **10b**).

**Scheme 2:**
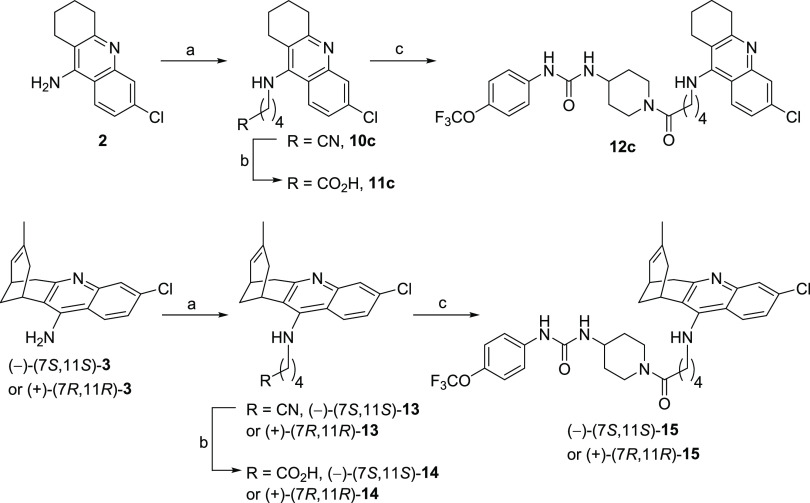
Synthesis of Hybrids **12c**, (−)-(7*S*,11*S*)-**15**, and (+)-(7*R*,11*R*)-**15** Reagents and conditions: (a) KOH, DMSO, 4 Å molecular sieves, heat gun every 10 min during 1 h, then RT, 1 h, then 5-bromovaleronitrile, DMSO, RT, overnight, 83% (**10c**), 54% [(−)-**13**], 62% [(+)-**13**]; (b) KOH, MeOH, reflux, 5 h, then H_2_O, reflux, overnight, then HCl/Et_2_O; (c) **11c**, (−)-**14** or (+)-**14**, EDC·HCl, HOBt, Et_3_N, 10:1 EtOAc/DMF, 10 min, RT, then **6**·HCl, RT, overnight, 79% (**12c**, over two steps from **10c**), 76% [(−)-**15**, over two steps from (−)-**13**], 40% [(+)-**15**, over two steps from (+)-**13**].

The synthesis of the target hybrids was carried out by EDC/HOBt-promoted amide coupling of the TPPU-derived piperidine **6** with a 6-chlorotacrine- or huprine-derived carboxylic acid (Schemes 1 and 2).

Piperidine **6**^31^ was obtained by reaction of 4-(trifluoromethoxy)phenyl isocyanate, **4**, with 4-amino-1-benzylpiperidine followed by debenzylation (Scheme 1). A convenient route to the carboxylic acids involved the base-catalyzed alkylation of 6-chlorotacrine or huprine Y with an ω-bromoalkanenitrile followed by hydrolysis as it was carried out for the synthesis of the tetramethylene-linked hybrids **12c**, (−)-(7*S*,11*S*)-**15**, and (+)-(7*R*,11*R*)-**15** (Scheme 2). However, this route was not applicable to the synthesis of the shorter homologues **12a** and **12b** due to the instability of the bromoalkanenitrile or lack of reactivity in the alkylation step. In these cases, the carboxylic acids were obtained by amination of the dichloroacridine **7**(36) with 2-aminoethanol or 3-aminopropanol followed by mesylation, nucleophilic substitution with NaCN, and hydrolysis (Scheme 1). All the target hybrids were converted into the hydrochloride salts, with which the chemical and biological characterizations were performed.

### In Vitro Evaluation of the Dual sEH/AChE Inhibitory Activity

In the development of multitarget compounds, some important challenges and risks must be faced up. Each pharmacophore should retain the ability to interact with its specific binding site on the biological target, while the second linked pharmacophore should at least not spoil the overall binding to that target or even, ideally, contribute with an additional binding at a secondary site. The same should happen in the second biological target to be hit, with reversed roles for both pharmacophores, so that the molecular hybridization would result in high potencies toward both targets, which is actually a very challenging task. Often, potencies of different orders of magnitude or balanced, albeit weak or moderate, are achieved toward the different targets, which would preclude the expected additive or synergistic effects in vivo. Very gratifyingly, most of the hybrids displayed well-balanced potencies, in the low nanomolar range, when tested in vitro toward the two recombinant human enzymes (hsEH and hAChE; Table 1).

**Table 1:** In Vitro Biological Activities of the Dual sEH/AChE Inhibitors toward Human and Murine Enzymes

compd	hsEH^a^ IC_50_ (nM)	hAChE^b^ IC_50_ (nM)	hBChE^b^ IC_50_ (nM)	msEH^a^ IC_50_ (nM)	mAChE^b^ IC_50_ (nM)
**12a**	0.4	14.5 ± 0.3	947 ± 6	12.1	35.4 ± 2.0
**12b**	1.0	2.71 ± 0.06	416 ± 35	15.0	4.01 ± 0.24
**12c**	4.6	12.9 ± 1.6	179 ± 90	22.5	4.12 ± 0.23
(−)-**15**	0.4	1.94 ± 0.67	615 ± 34	34.3	2.61 ± 0.16
(+)-**15**	3.1	1660 ± 450	179 ± 26	14.5	102 ± 18
**1**	3.7	115,000 ± 4000	n.a.^c^	2.8	-d
**2**	49,116	14.5 ± 0.9	505 ± 28	>50,000	19.8 ± 0.7
(−)-**3**	40,996	0.74 ± 0.06	222 ± 17	>50,000	0.62 ± 0.03
(+)-**3**	>50,000	321 ± 16	170 ± 17	>50,000	474 ± 22

a Data represent average IC_50_ values (nM) for inhibition of recombinant human and mouse sEH of three replicates. The fluorescent assay, as performed here, has a standard error between 10 and 20%, suggesting that the differences of 2-fold or greater are significant. Because of the limitations of the assay, it is difficult to distinguish among potencies <0.5 nM.^37^

b IC_50_ values (nM) for inhibition of recombinant human and mouse AChE and human serum BChE. Data represent mean values ± SEM of at least two experiments each performed in triplicate.

c Not active, i.e., % inhibition <10% at 100 μM.

d Not determined.

With IC_50_s in the subnanomolar to low nanomolar range (0.4–4.6 nM), all the hybrids retained the hsEH inhibitory activity of TPPU (IC_50_ 3.7 nM) or displayed an even higher potency (up to 9-fold in **12a** and (−)-**15**; Table 1). The sEH inhibitory activity increased with a shortened tether length (**12a** > **12b** > **12c**) and was found to be enantioselective for the huprine-based hybrids, with (−)-**15** being 8-fold more potent than its enantiomer. Regarding hAChE inhibition, hybrids **12a** and **12c** retained the high potency of 6-chlorotacrine (IC_50_ 14.5 nM), while **12b** was 5-fold more potent (Table 1). A marked enantioselectivity was found for the inhibition of hAChE by the huprine-based hybrids **15**, with (−)-(7*S*,11*S*)-**15** being 850-fold more potent than its enantiomer, in line with the eudismic ratio of huprine Y (430).^35^

Besides AChE, butyrylcholinesterase (BChE) undertakes a prominent role in ACh hydrolysis, and, hence, in cognitive impairment, when AD progresses.^38^ This makes inhibition of BChE another activity of interest for AD treatment.^39,40^ Like 6-chlorotacrine and huprine Y, all the hybrids showed submicromolar potencies toward human BChE (hBChE), with hybrid **12c** standing out by its potency (IC_50_ 179 nM) 3-fold higher than that of the parent 6-chlorotacrine (Table 1).

Even though this class of compounds is intended for human use, the preclinical development involves an in vivo proof of concept in an animal model, usually in mice. For that reason, we tested in vitro the inhibitory activity of the hybrids on mouse sEH and AChE (msEH and mAChE). This was deemed especially important in the case of sEH because the murine enzyme is known to be more sensitive to steric hindrance,^41^ which might be an issue taking into account that the dual sEH/AChE inhibitors are rather large molecules. Satisfactorily, all the compounds exhibited potent msEH inhibitory activity, in the 12–34 nM range, albeit not as potent as toward the human enzyme and not as potent as TPPU toward msEH (Table 1). Likewise, most hybrids were potent inhibitors of mAChE, with IC_50_s slightly higher than those for hAChE, except **12c** (IC_50_ 4.12 nM), which was 3-fold more potent toward the murine enzyme and a 5-fold more potent mAChE inhibitor than the parent 6-chlorotacrine (Table 1).

### Mechanistic Insights into the Dual Inhibition of hsEH and hAChE

**Figure 3:**
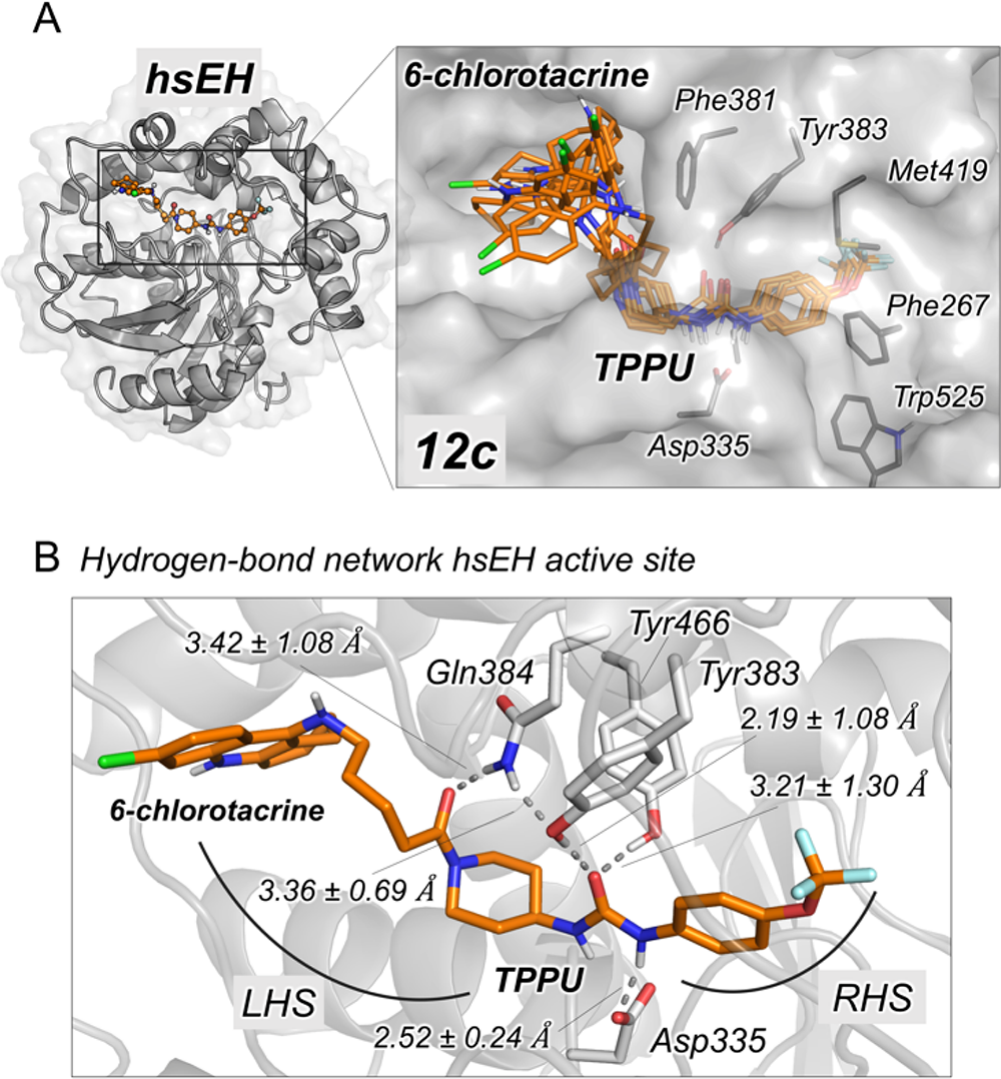
Binding of **12c** to the active site of hsEH. (A) Representation of the hsEH structure (PDB: 4OD0) with the binding mode of **12c** to the active site of hsEH as revealed by MD simulations. Overlay of the most representative clusters obtained from the MD simulations shows the flexibility of the 6-chlorotacrine moiety. (B) Representative structure of **12c** bound in the active site of hsEH obtained from the most visited conformations along the MD simulations. The 6-chlorotacrine moiety occupies the entrance of the LHS pocket. The most relevant hydrogen bond interactions between the TPPU scaffold of **12c** and the hsEH active site are highlighted. Average distances (in Å) obtained from the three replicas of 250 ns of MD are represented. Residues of hsEH are highlighted in gray, and **12c** is shown in orange sticks.

The mechanism of action of the dual inhibitor **12c**, selected based on its overall biological profile (see below), within hsEH and hAChE was explored by docking and molecular dynamics (MD) simulations. To elucidate how the incorporation of the 6-chlorotacrine moiety impacts the binding of the urea-based TPPU scaffold, the preferred binding mode of **12c** in the active site of hsEH was first explored. Molecular docking calculations indicate that the TPPU scaffold of **12c** is oriented with the urea moiety interacting with Asp335, Tyr383, and Tyr466 catalytic residues, the piperidine group occupying the LHS pocket, and the trifluoromethoxyphenyl group placed in the RHS pocket, as observed in the X-ray crystal structure of the hsEH–TPPU complex (PDB: 4OD0). The tetramethylene linker of **12c** spans the LHS pocket with the 6-chlorotacrine moiety being partially exposed to the solvent at the entrance of the LHS pocket (Figure 3). MD simulations (three replicas of 250 ns) starting from the orientation predicted by molecular docking showed that the TPPU scaffold remains stable in the hsEH active site through a network of hydrogen bonds and hydrophobic interactions, i.e., strong hydrogen bonds of the urea group with Asp335 and Tyr383 and transient interactions with Tyr466; a hydrogen bond between the oxygen of the carbonyl attached to the piperidine nitrogen and the amide group of Gln384; and hydrophobic interactions of the trifluoromethoxyphenyl group with Phe267, Met419, Phe497, and Trp525 at the RHS (Figure 3). The 6-chlorotacrine scaffold displays significant flexibility adopting different conformations in the entrance of the LHS pocket with the chlorine pointing toward the solvent (Figure 3A), interacting with different hydrophobic residues of α-helices Ser370–Glu389 and Val500–Ile511, with Phe381 being the one displaying more persistent interactions. Thus, computational simulations confirmed that the linker–chlorotacrine moiety, attached to the piperidine ring of TPPU, does not significantly interfere with the stability and potency of the TPPU scaffold in the hsEH–**12c** complex.

**Figure 4:**
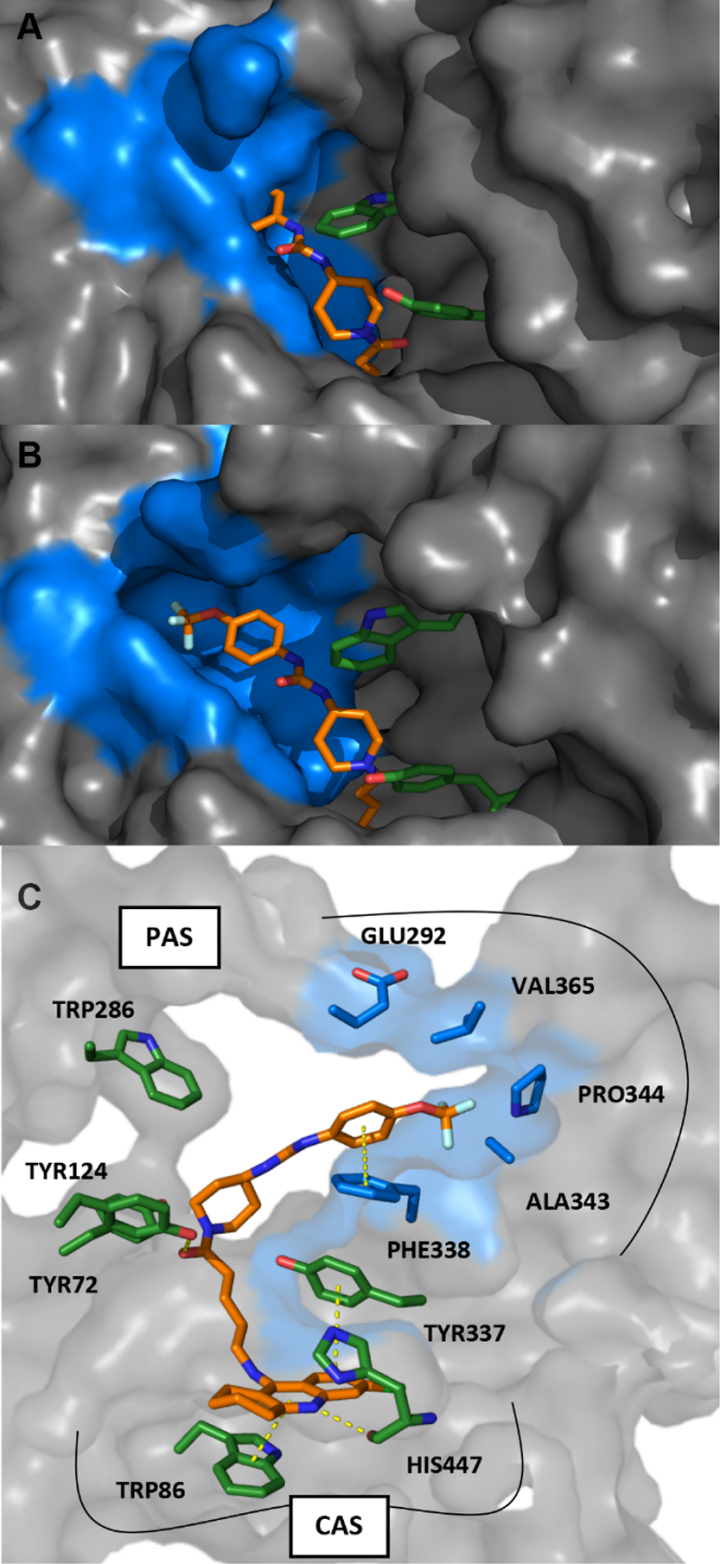
Binding of **12c** to the active site of hAChE. (A) Superposition of a representative structure of the binding of compound **12c** in the PAS with the crystal structure of AChE PDB: 6O4X. (B) Representative structure of the binding mode of **12c** to the PAS as revealed by MD simulations. (C) Proposed binding mode of **12c** to the active site of hAChE. Residues of the PAS are highlighted in blue, while other relevant residues of the enzyme are represented in green, and **12c** is shown as orange sticks.

The primary binding site of 6-chlorotacrine within AChE is the CAS, at the bottom of the gorge. Given the length of compound **12c**, we hypothesized that it could act as a dual binding site inhibitor, reaching both the CAS and the PAS. The TPPU moiety contains an aromatic ring that might establish π–π stacking interactions with aromatic residues at the PAS, notably Trp286.^42^ To account for the plasticity of the PAS during docking calculations, three different AChE models were generated, hereby referred to as AChE_6O4X_, AChE_1Q83_, and AChE_2CKM_, which retained the side-chain orientation of the AChE–9-aminoacridine complex (PDB: 6O4X) in the CAS but differed on the orientation of Trp286 at the PAS. After docking calculations, seven plausible binding modes with similar score and comparable patterns of intermolecular interactions were identified (Figure S1 of the Supporting Information). Further study of these binding modes by means of three independent replicates of 1 μs long MD simulations, amounting to 21 μs of cumulative sampling, surprisingly revealed that 12 out of the 21 systems retained the main binding features at the CAS (Figure S2 of the Supporting Information), with root mean square deviation (RMSD) values with respect to the initial structure for the 6-chlorotacrine moiety below 2 Å over the last 100 ns of each trajectory (Figure S3 of the Supporting Information), whereas interactions at the PAS were not stable, with all simulations losing the starting orientations and interaction patterns throughout the simulation. Indeed, visual inspection of the final structures revealed a substantial reorientation of the TPPU moiety in all of them. Specifically, in most final structures, the TPPU moiety shifted away from its initial position close to Trp286 and moved toward a previously collapsed cavity located between loops 337–344 and 292–294 (Figure 4A,B). The opening of this cryptic pocket resulted in a ca. 51% increase in the volume of the PAS (from 214 ± 83.6 Å^3^ during the first 100 ns up to 324 ± 73.1 Å^3^ over the last 100 ns) and led to a very hydrophobic cavity. In fact, the pocket is delineated by the side chains of residues Val294, Phe338, Leu339, Val365, and Val402 and also contains the backbone carbonyl atoms of residues Ser293 and Phe338, thus suggesting that proper functionalization of the moieties accommodated in this pocket could result in strong anchoring interactions.^43^ Out of the 21 independent MD simulations, 13, starting from different initial structures, converged to the same binding mode, reaching values below 4 Å of RMSD relative to the reference structure (Figure S4 of the Supporting Information). Further analysis confirmed that the rearrangement of the ligand happened gradually along the simulations for different systems and preceded the opening of the aforementioned pocket in the protein (Figure 4C and Movie S1 of the Supporting Information), which was not present in the starting crystal structure (Figure 4A) nor was identified when mdpocket and visual inspection were combined to analyze the structures of AChE from a human (*Homo sapiens*), common mouse (*Mus musculus*), pacific electric ray (*Torpedo californica*), and electric eel (*Electrophorus electricus*) available in the PDB (Table S1 of the Supporting Information). Furthermore, this pocket did not spontaneously open during control simulations of the AChE–donepezil complex, which, as expected, retained the characteristic arrangements and interaction patterns observed in crystallographic structures (Figure S5 of the Supporting Information). Taken together, these results support the existence of an inducible cryptic pocket in the PAS of AChE that can be used to accommodate small molecules, thus opening new avenues for the design of dual-site AChE inhibitors.

The interaction of **12c** at the PAS of AChE was experimentally demonstrated by assessing its ability to displace the PAS-selective ligand propidium. Indeed, **12c** was able to displace propidium from PAS, although with a significantly lower affinity (*K*_D_ = 3.95 ± 0.35 μM) than that of propidium (*K*_D_ = 0.7 μM).

Thus, these computational and experimental studies confirmed that the dual sEH/AChE inhibitor **12c** occupies the whole long cavities of both enzymes, with the original pharmacophore interacting at the main binding site, the linker helping to span the cavity, and the second pharmacophore contributing to additional interactions at a known or at a so far unknown secondary or peripheral site.

### Blood–Brain Barrier Penetration, Aqueous Solubility, and Neurotoxicity of the Dual sEH/AChE Inhibitors

After confirming the balanced nanomolar potencies at sEH and AChE, we addressed another potential issue of multitarget compounds, especially those designed by the linked-pharmacophore approach, namely, the potential negative impact that the relatively high molecular weight and lipophilicity of the resulting hybrids might have on their physicochemical and pharmacokinetic properties and on their cytotoxicity.^44−46^ To this end, the blood–brain barrier (BBB) permeability, aqueous solubility, and cytotoxicity of the four best hybrids were assessed.

Satisfactorily, the dual sEH/AChE inhibitors are able to cross the BBB, as measured by the well-established parallel artificial membrane permeation assay for the BBB (PAMPA-BBB), which models passive diffusion.^47^ Indeed, the hybrids have permeability (*P*_e_) values around 9 × 10^–6^ cm s^–1^, which is almost double the cutoff value that predicts a high brain permeability (CNS+: 5.2 × 10^–6^ cm s^–1^) (Table 2). Therefore, despite the molecular weight over 500 of the dual sEH/AChE inhibitors, brain penetration should not be an issue so that they should be able to reach their CNS targets.

**Table 2:** In Vitro BBB Permeability, Aqueous Solubility, and Cytotoxicity of the Dual sEH/AChE Inhibitors

compd	BBB permeability^a^*P*_e_ (10^–6^ cm s^–1^)	aqueous solubility^b^ (μM)	toxicity to SH-SY5Y cells^c^ LD_50_ (μM)
**12a**	9.2 ± 0.2 (CNS+)	42.6	92.8 ± 52.3
**12b**	9.1 ± 0.1 (CNS+)	13.7	>100
**12c**	8.4 ± 0.7 (CNS+)	7.5	>100
(−)-**15**	9.9 ± 0.3 (CNS+)	5.6	>100

a Data represent mean values ± SD of three experiments each performed in triplicate. High permeation, CNS+: *P*_e_ > 5.2; low permeation, CNS–: *P*_e_ < 2.0; uncertain permeation, CNS±: 5.2 > *P*_e_ > 2.0.

b Solubility in a 1% DMSO/99% phosphate-buffered saline (PBS) buffer after 2 h at 37 °C.

c Tested by propidium iodide staining after 24 h of incubation in SH-SY5Y cells. Data represent mean values ± SD of three experiments each performed in triplicate.

Aqueous solubility influences the ability of a drug to be absorbed, thereby affecting its bioavailability.^48^ The kinetic solubility of the hybrids was in the 5.6–42.6 μM range (Table 2). Expectably, the solubility clearly decreased with increased tether length, and, hence, with increased lipophilicity. Even though a higher solubility would be desirable for most hybrids, they are soluble at concentrations 3–5 orders of magnitude above the IC_50_s toward their biological targets, so solubility should not be an issue for the dual sEH/AChE inhibitors.

High lipophilicity is usually associated with cytotoxicity.^46^ Despite the higher than desirable lipophilicity (log *P* > 5) of the hybrids, they are essentially non-toxic to human neuroblastoma SH-SY5Y cells, displaying LD_50_ values around or above 100 μM (Table 2), i.e., more than 4–5 orders of magnitude higher than their IC_50_s for sEH and AChE inhibition.

### Microsomal Stability of the Dual sEH/AChE Inhibitors

To get further insight into the DMPK properties of the dual sEH/AChE inhibitors, their stability in human, mouse, and rat liver microsomes was determined. The stability parameters are shown in Table 3. The hybrid **12c** was clearly the most stable compound in human microsomes, with 82, 74, and 55% of the initial amount of the compound remaining non-metabolized after 10, 20, and 60 min of incubation at 37 °C, respectively, and a half-life (*t*_1/2_) of 91 min. It is the sole compound of the family that can be considered as a low clearance compound [intrinsic clearance (CL_int_) < 15 μL min^–1^ mg protein^–1^].^49^ Compound **12c** was also the most stable in rat microsomes, with 100, 79, 50, and 21% of the compound remaining unaltered after 10, 20, 40, and 60 min of incubation, respectively. In mouse microsomes, **12c** was slightly more stable than the rest of compounds, even though all of them were almost completely metabolized after 1 h of incubation. After 10 min of incubation with mouse liver microsomes, the amount of **12c** was 38% but it dropped to 4% after 20 min of incubation.

**Table 3:** Stability of the Dual sEH/AChE Inhibitors in Human, Mouse, and Rat Liver Microsomes at 37 °C^a^

	human microsomes				mouse microsomes				rat microsomes		
compd	%	*t*_1/2_	CL_int_		%	*t*_1/2_	CL_int_		%	*t*_1/2_	CL_int_
**12a**	4	12	57		1	6	109		4	12	56
**12b**	34	36	19		1	9	78		2	10	69
**12c**	55	91	8		2	4	155		21	26	26
(−)-**15**	1	5	144		0.4	4	172		4	13	54

a Data represent mean values of two experiments each performed in triplicate: % is the percentage of the remaining compound after 1 h of incubation with microsomes at 37 °C; *t*_1/2_ is the half-life in min; CL_int_ is the intrinsic clearance in μL min^–1^ mg protein^–1^.

### In Vivo Proof of Concept of a Dual sEH/AChE Inhibitor in a Mouse Model of Alzheimer’s Disease

**Figure 5:**
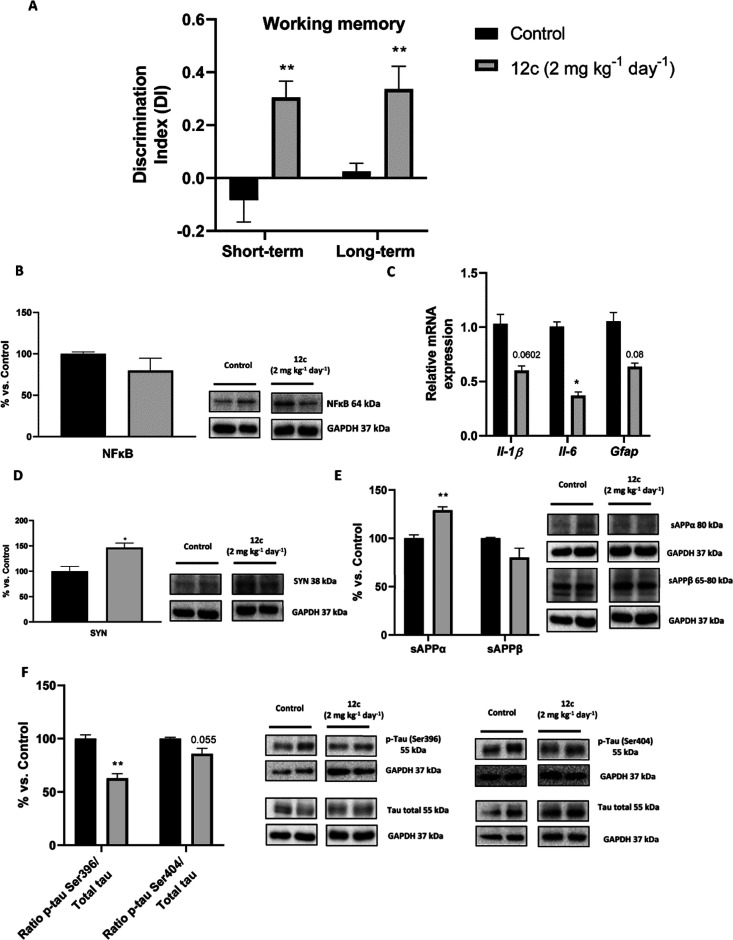
Effects of **12c** (2 mg kg^–1^ day^–1^) treatment in SAMP8 mice (control, black bars; treated, gray bars). (A) NORT results for short- and long-term memory. (B) Hippocampal tissue quantifications and representative western blot for NFkβ. (C) Gene expression of inflammatory markers Il1-β, Il-6, and *Gfap*. (D) Hippocampal tissue quantifications and representative western blot for synaptophysin. (E) Hippocampal sAPPα and sAPPβ protein levels. (F) Ratio p-Tau (Ser396 and Ser404). Values in bar graphs are adjusted to 100% for protein levels of the SAMP8 control group; gene expression determined by real-time PCR; values represented are mean ± SEM; *p < 0.05; **p < 0.01 vs control.

Based on the in vitro biological activity and DMPK property profile, compound **12c** was selected for further studies. This compound exhibited the desired balanced nanomolar potencies at sEH and AChE, it was the most potent at hBChE, and also displayed high brain permeability, moderate aqueous solubility, lack of neurotoxicity, and the highest microsomal stability of the series. To test our hypothesis that the dual inhibition of sEH and AChE is a new approach for the efficient treatment of AD, a chronic in vivo efficacy study with **12c** was performed using senescence-accelerated mouse-prone 8 (SAMP8), a well-established mouse model of late-onset AD (see the experimental timeline in Figure S6 of the Supporting Information). This model recapitulates some of the main AD hallmarks such as age-related cognitive impairment, neuroinflammation, abnormal amyloid precursor protein (APP) processing, tau pathology, and oxidative stress.^50,51^ We administered **12c** orally to SAMP8 mice daily during 4 weeks at a dose of 2 mg kg^–1^, a dosing regimen that had been successfully applied to other classes of multitarget agents developed in our group.^10,40^ After 4 weeks of oral administration of this low dose of **12c** (2 mg kg^–1^ day^–1^), it significantly ameliorated short-term and long-term working memory in treated SAMP8 mice compared with vehicle-treated mice (Figure 5A), as evidenced by a greatly increased discrimination index (DI), a measure of the ability of mice to differentiate between a known and a new object, when using the novel object recognition test (NORT). Because both **12c**-treated and control mice had spent the same time exploring the two objects during the familiarization phase of the NORT test (Figure S7 of the Supporting Information), the observed increase in the DI in treated mice must unambiguously result from a cognition enhancing effect by **12c**.

Apart from the beneficial effects on memory, a further primary endpoint pursued with the dual sEH/AChE inhibition approach was reducing neuroinflammation. Indeed, treatment with **12c** diminished protein levels or gene expression of different markers of neuroinflammation in the hippocampus of SAMP8 mice. Thus, protein levels of nuclear factor kβ (NFkβ) were reduced in mice treated with **12c** relative to untreated mice but without reaching statistical significance (Figure 5B). In addition, gene expression analysis showed that **12c** led to a significant reduction of interleukin 6 (Il-6) gene expression in comparison with control mice (Figure 5C). Also, a tendency toward reduced gene expression of other inflammatory markers, such as interleukin 1β (Il-1β) and glial fibrillary acidic protein (*Gfap*), was found in **12c**-treated mice (Figure 5C).

Analysis of other molecular markers showed that **12c** significantly increased hippocampal levels of the synaptic protein synaptophysin (SYN) (Figure 5D), which is indicative of a beneficial effect on synaptic plasticity. We also investigated changes in other key AD hallmarks, namely, APP processing and tau phosphorylation. Treatment with **12c** led to a significant increase in the hippocampal protein levels of sAPPα, a neuroprotective secreted ectodomain of APP arising from non-amyloidogenic processing (Figure 5E), and to a tendency toward reduced levels of sAPPβ, a neurotoxic peptide that results from the amyloidogenic cleavage of APP (Figure 5E). SAMP8 mice treated with **12c** also showed a significant site-specific reduction of tau phosphorylation at Ser396 and to a slightly decreased phosphorylation at Ser404, in that case without reaching significance (Figure 5F).

## Conclusions

Multitarget drugs are attracting increasing research interest. Their therapeutic potential is great. Their design may seem simple, but it is not devoid of very important challenges and risks, prominently the need for potent and balanced activity at the different targets and favorable DMPK properties, which are plaguing the successful translation of many multitarget compounds to the clinic. Several sEH inhibitor-based multitarget compounds have been recently designed to treat inflammation and pain,^3,52−54^ but their potential use against AD and the dual targeting of sEH and AChE have not been explored so far, to the best of our knowledge. Here, we disclose a class of dual sEH/AChE inhibitors, with potential to derive both disease-modifying and symptomatic effects for AD treatment. The lead compound showed balanced nanomolar potencies at both targets and favorable DMPK properties, including high brain permeability, and the absence of toxicity toward neuronal cells at concentrations more than 4 orders of magnitude higher than those required for dual molecular target inhibition, thereby overcoming the main limitations of many multitarget compounds. Gratifyingly, this favorable profile translated to favorable effects in an in vivo proof of concept. Overall, not only the pursued primary endpoints, i.e., cognition enhancement and reduction of neuroinflammation, were reached upon chronic oral treatment of SAMP8 mice with **12c**, but it also elicited other beneficial effects on key pathological hallmarks of AD, such as amyloid, tau, and synaptic dysfunction. These results validate sEH/AChE dual inhibition as a promising strategy to alleviate the symptomatology while addressing key underlying mechanisms of AD and make compound **12c** a promising candidate with this innovative mechanism of action.^55^

## Experimental Section

### Chemistry

#### General Information

All reagents and solvents were purchased from commercial suppliers (Merck, Acros, Cymit) unless otherwise stated and used without further purification. The progress of the reactions was monitored by thin-layer chromatography using aluminum-backed sheets with silica gel 60 F254 (Merck, ref 1.05554), with CH_2_Cl_2_/MeOH/50% aq NH_4_OH, hexane/EtOAc/50% aq NH_4_OH, or hexane/CH_2_Cl_2_/50% aq NH_4_OH mixtures as the solvent system. The spots were visualized with UV light and/or 1% aq KMnO_4_ followed by charring with a heat gun. Column chromatography was performed on silica gel 60 AC.C (35–70 mesh, Carlo Erba, ref 2000027). Melting points were determined in open capillary tubes with an MFB 595010 M Gallenkamp melting point apparatus. IR spectra were run on a PerkinElmer Spectrum RX I spectrophotometer: Absorption values were expressed as wavenumbers (cm^–1^), and only significant absorption bands were given. ^1^H NMR spectra (400 MHz)/^13^C NMR spectra (100.6MHz) were recorded on a Varian Mercury 400 spectrometer at the Centres Científics i Tecnològics of the University of Barcelona (CCiTUB): The chemical shifts were reported in ppm (δ scale) relative to solvent signals (CD_3_OD at 3.31 and 49.0 ppm and CDCl_3_ at 7.26 and 77.16 ppm, in the ^1^H and ^13^C NMR spectra, respectively); coupling constants were reported in hertz (Hz); assignments given for the NMR spectra of the new compounds have been carried out on the basis of COSY ^1^H/^1^H (standard procedures) and COSY ^1^H/^13^C (gHSQC) experiments; the *syn* (*anti*) notation of the protons at position 13 of the huprine moiety of compounds **13** and **15** denotes that they are on the same (different) side of the quinoline moiety with respect to the cyclohexene ring. High-resolution mass spectra (HRMS) were carried out at the CCiTUB with an LC/MSD TOF Agilent Technologies spectrometer. Optical rotations were measured on a PerkinElmer model 241 polarimeter. Analytical RP-HPLC was performed with an HPLC Agilent 1260 Infinity II LC/MSD coupled to a photodiode array and mass spectrometer. Samples (5 μL, 0.5 mg/mL) in a 1:1 mixture of water with 0.05% formic acid (A) and acetonitrile with 0.05% formic acid (B) were injected using an Agilent Poroshell 120 EC-C18 (2.7 μm, 50 mm × 4.6 mm) column at 40 °C. The mobile phase was a mixture of A and B, with a flow 0.6 mL/min, using the following gradients: from 95%A–5%B to 100%B in 3 min; 100%B for 3 min; from 100%B to 95%A–5%B in 1 min; and 95%A–5%B for 3 min. Purity is given as % of absorbance at 254 nm. All compounds that were subjected to pharmacological evaluation are >95% pure by HPLC. Intermediates **5**,^56^**6**,^31^**8a**,**b**,^57^**9a**,**b**,^57^**10b**,**c**,^58^ and **11b**(58) were prepared following the described procedures (see the Supporting Information).

#### 3-[(6-Chloro-1,2,3,4-tetrahydroacridin-9-yl)amino]propanenitrile (**10a**)

A solution of mesylate **9a** (383 mg, 1.08 mmol) and NaCN (53 mg, 1.08 mmol) in dry DMF (3 mL) was heated at 35 °C for 2 h, quenched with water (4 mL) and 1 N NaOH (6 mL), and extracted with EtOAc (3 × 5 mL). The combined organic extracts were washed with water (3 × 5 mL) and brine (5 mL), dried over anhydrous Na_2_SO_4_, and evaporated to dryness. The resulting residue was purified through column chromatography (35–70 μm silica gel, hexane/EtOAc/Et_3_N mixtures, gradient elution). On elution with hexane/EtOAc/Et_3_N 86:14:0.2 to 75:25:0.5, nitrile **10a** (157 mg, 51% yield) was obtained as a yellow oil: *R*_f_ 0.3 (hexane/EtOAc/50% aq NH_4_OH 6:4:0.02).

The analytical sample of **10a**·HCl was prepared as follows. A solution of **10a** (28 mg) in CH_2_Cl_2_ (2 mL) was filtered through a 0.2 μm PTFE filter, treated with a solution of HCl in Et_2_O (1.17 M, 1 mL), and evaporated to dryness. After washing of the resulting solid with hexane (2 × 2 mL) and pentane (2 × 2 mL) and drying at 45 °C/2 Torr for 3 days, **10a**·HCl (29 mg) was obtained as a pale toasted solid: mp 278 °C (dec); ^1^H NMR (400 MHz, CD_3_OD) δ (ppm): 1.95–2.03 (complex signal, 4H, 2′-H_2_, 3′-H_2_), 2.78 (m, 2H, 1′-H_2_), 3.00 (t, *J* = 6.4 Hz, 2H, 2-H_2_), 3.06 (m, 2H, 4′-H_2_), 4.24 (t, *J* = 6.4 Hz, 2H, 3-H_2_), 4.85 (s, NH, ^+^NH), 7.63 (dd, *J* = 9.2 Hz, *J*′ = 2.0 Hz, 1H, 7′-H), 7.83 (d, *J* = 2.0 Hz, 1H, 5′-H), 8.37 (d, *J* = 9.2 Hz, 1H, 8′-H); ^13^C NMR (100.6 MHz, CD_3_OD) δ (ppm): 19.6 (CH_2_, C2), 21.7 (CH_2_, C3′), 22.8 (CH_2_, C2′), 25.2 (CH_2_, C1′), 29.5 (CH_2_, C4′), 44.8 (CH_2_, C3), 114.9 (C, C9a′), 116.1 (C, C8a′), 118.8 (C, C1), 119.4 (CH, C5′), 127.5 (CH, C7′), 128.2 (CH, C8′), 140.2 (C, C6′), 140.4 (C, C10a′), 153.6 (C, C4a′), 158.2 (C, C9′); IR (ATR) ν (cm^–1^): 3300–2500 (max at 3226, 2943, 2727, N–H, ^+^N–H, C–H st), 2246 (C ≡ N st); HRMS (ESI): *m*/*z* calcd for C_16_H_16_^35^ClN_3_ + H^+^: 286.1106 [M + H]^+^; found: 286.1101.

#### (−)-(7*S*,11*S*)-5-[(3-Chloro-6,7,10,11-tetrahydro-9-methyl-7,11-methanocycloocta[*b*]quinolin-12-yl)amino]pentanenenitrile [(−)-(7*S*,11*S*)-**13**]

A mixture of (−)-huprine Y, (−)-(7*S*,11*S*)-**3** (297 mg, 1.04 mmol), finely powdered KOH (85% purity reagent, 279 mg, 4.23 mmol), and 4 Å molecular sieves in dry DMSO (5 mL) was stirred, heating every 10 min with a heat gun for 1 h and at room temperature for an additional 1 h, and then treated with a solution of 5-bromovaleronitrile (0.16 mL, 222 mg, 1.37 mmol) in dry DMSO (1 mL). The reaction mixture was stirred at room temperature overnight, then diluted with 5 N NaOH (30 mL), and extracted with EtOAc (3 × 20 mL). The combined organic layers were washed with water (3 × 30 mL) and brine (30 mL), dried over anhydrous Na_2_SO_4_, and evaporated to dryness to provide a brown oil (349 mg), which was subjected to column chromatography purification (35–70 μm silica gel, hexane/CH_2_Cl_2_/Et_3_N mixtures, gradient elution). On elution with hexane/CH_2_Cl_2_/Et_3_N 86:14:0.4 to 75:25:1, impure (−)-**13** (250 mg) was isolated. Recrystallization from EtOAc (2 mL) afforded a white solid consisting of unreacted (−)-huprine Y, with the mother liquors being enriched in the desired nitrile. After evaporation of the mother liquors at reduced pressure, the recrystallization process was repeated (EtOAc, 1 mL). Evaporation of the final mother liquors afforded pure (−)-**13** (206 mg, 54% yield) as a yellowish oil: *R*_f_ 0.62 (hexane/CH_2_Cl_2_/50% aq NH_4_OH 6:4:0.04); [α]^20^_D_ = −95 (*c* 0.48 in CH_2_Cl_2_); ^1^H NMR (400 MHz, CDCl_3_) δ (ppm): 1.52 (s, 3H, 9′-CH_3_), 1.75–1.96 (m, 6H, 3-H_2_, 4-H_2_, 10′-H_*endo*_, 13′-H_*syn*_), 2.06 (dm, *J* = 12.4 Hz, 1H, 13′-H_*anti*_), 2.44 (t, *J* = 6.8 Hz, 2H, 2-H_2_), 2.56 (dd, *J* = 16.8 Hz, *J*′ = 5.6 Hz, 1H, 10′-H_*exo*_), 2.75 (m, 1H, 7′-H), 3.01 (ddd, *J* = 17.6 Hz, *J*′ = *J*″ = 2.0 Hz, 1H, 6′-H_*endo*_), 3.16 (dd, *J* = 17.6 Hz, *J*′ = 5.6 Hz, 1H, 6′-H_*exo*_), 3.31 (m, 1H, 11′-H), 3.48 (dt, *J* = *J*′ = 7.2 Hz, 2H, 5-H_2_), 3.90 (br s, 1H, NH), 5.54 (br d, *J* = 5.6 Hz, 1H, 8′-H), 7.30 (dd, *J* = 8.8 Hz, *J*′ = 2.4 Hz, 1H, 2′-H), 7.88 (d, *J* = 8.8 Hz, 1H, 1′-H), 7.89 (d, *J* = 2.4 Hz, 1H, 4′-H); IR (ATR) ν (cm^–1^): 3377 (N–H st), 2242 (C ≡ N st); HRMS (ESI): *m*/*z* calcd for C_22_H_24_^35^ClN_3_ + H^+^: 366.1732 [M + H]^+^; found: 366.1734.

#### (+)-(7*R*,11*R*)-5-[(3-Chloro-6,7,10,11-tetrahydro-9-methyl-7,11-methanocycloocta[*b*]quinolin-12-yl)amino]pentanenenitrile [(+)-(7*R*,11*R*)-**13**]

This compound was prepared as described for (−)-**13**. From (+)-huprine Y, (+)-(7*R*,11*R*)-**3** (250 mg, 0.88 mmol), finely powdered KOH (85% purity reagent, 191 mg, 2.89 mmol), and 5-bromovaleronitrile (0.11 mL, 153 mg, 0.94 mmol), a brown oily residue (309 mg) was obtained and subjected to column chromatography purification (35–70 μm silica gel, hexane/CH_2_Cl_2_/Et_3_N mixtures, gradient elution). On elution with hexane/CH_2_Cl_2_/Et_3_N 96:4:0.4, (+)-**13** (97 mg) was isolated. An additional fraction of impure (+)-**13** (145 mg) was also obtained. The latter product was taken up in 5 N HCl (15 mL) and washed with Et_2_O (3 × 10 mL). The acidic aqueous phase was alkalinized with NaOH pellets (until pH = 10) and extracted with CH_2_Cl_2_ (3 × 10 mL). The combined organic extracts were dried over anhydrous Na_2_SO_4_ and evaporated at reduced pressure to afford an additional crop of (+)-**13** (102 mg, 62% total yield) as a yellowish oil: *R*_f_ 0.62 (hexane/CH_2_Cl_2_/50% aq NH_4_OH 6:4:0.04); [α]^20^_D_ = +95 (*c* 0.13 in CH_2_Cl_2_); the ^1^H NMR spectrum of (+)-**13** coincided with that of its enantiomer (−)-**13**; IR (ATR) ν (cm^–1^): 3377 (N–H st), 2243 (C ≡ N st); HRMS (ESI): *m*/*z* calcd for C_22_H_24_^35^ClN_3_ + H^+^: 366.1732 [M + H]^+^; found: 366.1731.

#### 3-[(6-Chloro-1,2,3,4-tetrahydroacridin-9-yl)amino]propanoic Acid (**11a**)

A suspension of nitrile **10a** (150 mg, 0.52 mmol) in 5 N HCl (13 mL) was heated under reflux for 3.5 h. The resulting yellow solution was evaporated to dryness to provide crude **11a** (171 mg), as a pale yellow solid, in the form of the quinoline hydrochloride salt that was used in the following step without further purification: ^1^H NMR (400 MHz, CD_3_OD) δ (ppm): 1.90–2.00 (complex signal, 4H, 2′-H_2_, 3′-H_2_), 2.68 (m, 2H, 1′-H_2_), 2.85 (t, *J* = 6.4 Hz, 2H, 2-H_2_), 3.02 (m, 2H, 4′-H_2_), 4.22 (t, *J* = 6.4 Hz, 2H, 3-H_2_), 4.85 (s, NH, ^+^NH), 7.57 (dd, *J* = 9.2 Hz, *J*′ = 2.0 Hz, 1H, 7′-H), 7.81 (d, *J* = 2.0 Hz, 1H, 5′-H), 8.39 (d, *J* = 9.2 Hz, 1H, 8′-H); HRMS (ESI): *m*/*z* calcd for C_16_H_17_^35^ClN_2_O_2_ + H^+^: 305.1051 [M + H]^+^; found: 305.1048.

#### 5-[(6-Chloro-1,2,3,4-tetrahydroacridin-9-yl)amino]pentanoic Acid (**11c**)

A solution of nitrile **10c** (289 mg, 0.92 mmol) in MeOH (1.5 mL) was treated with a 40% KOH solution in MeOH (2.5 mL). The mixture was stirred under reflux for 5 h, treated with water (4 mL), and stirred at reflux overnight. The resulting yellow solution was cooled down to room temperature, evaporated to dryness, then treated with a solution of HCl in Et_2_O (1.17 M, 6 mL), and evaporated to dryness to afford crude **11c** (789 mg), in the form of quinoline hydrochloride salt, as a yellow solid that was used in the following step without further purification: ^1^H NMR (400 MHz, CD_3_OD) δ (ppm): 1.72 (tt, *J* = *J*′ = 7.2 Hz, 2H, 3-H_2_), 1.88 (tt, *J* = *J*′ = 7.2 Hz, 2H, 4-H_2_), 1.92–2.02 (m, 4H, 2′-H_2_, 3′-H_2_), 2.38 (t, *J* = 7.2 Hz, 2H, 2-H_2_), 2.69 (m, 2H, 1′-H_2_), 3.01 (m, 2H, 4′-H_2_), 3.96 (t, *J* = 7.2 Hz, 2H, 5-H_2_), 4.85 (s, OH, NH, ^+^NH), 7.56 (dd, *J* = 9.2 Hz, *J*′ = 2.4 Hz, 1H, 7′-H), 7.80 (d, *J* = 2.4 Hz, 1H, 5′-H), 8.39 (d, *J* = 9.2 Hz, 1H, 8′-H); HRMS (ESI): *m*/*z* calcd for C_18_H_21_^35^ClN_2_O_2_ + H^+^: 333.1364 [M + H]^+^; found: 333.1372.

#### (−)-(7*S*,11*S*)-5-[(3-Chloro-6,7,10,11-tetrahydro-9-methyl-7,11-methanocycloocta[*b*]quinolin-12-yl)amino]pentanoic Acid [(−)-(7*S*,11*S*)-**14**]

This compound was prepared as described for **11c**. From nitrile (−)-**13** (206 mg, 0.56 mmol), crude (−)-**14** (1.55 g), in the form of quinoline hydrochloride salt, was obtained as a yellow solid that was used in the following step without further purification: ^1^H NMR (400 MHz, CD_3_OD) δ (ppm): 1.58 (s, 3H, 9′-CH_3_), 1.73 (tt, *J* = *J*′ = 7.2 Hz, 2H, 3-H_2_), 1.85–2.00 (m, 4H, 4-H_2_, 10′-H_*endo*_, 13′-H_*syn*_), 2.07 (dm, *J* = 11.6 Hz, 1H, 13′-H_*anti*_), 2.34 (t, *J* = 7.2 Hz, 2H, 2-H_2_), 2.56 (dd, *J* = 17.6 Hz, *J*′ = 5.2 Hz, 1H, 10′-H_*exo*_), 2.76 (m, 1H, 7′-H), 2.87 (br d, *J* = 17.6 Hz, 1H, 6′-H_*endo*_), 3.19 (dd, *J* = 17.6 Hz, *J*′ = 5.6 Hz, 1H, 6′-H_*exo*_), 3.47 (m, 1H, 11′-H), 3.97 (td, *J* = 7.2 Hz, *J*′ = 2.4 Hz, 2H, 5-H_2_), 4.85 (s, OH, NH, ^+^NH), 5.57 (br d, *J* = 5.6 Hz, 1H, 8′-H), 7.53 (dd, *J* = 9.2 Hz, *J*′ = 2.4 Hz, 1H, 2′-H), 7.76 (d, *J* = 2.4 Hz, 1H, 4′-H), 8.39 (d, *J* = 9.2 Hz, 1H, 1′-H); HRMS (ESI): *m*/*z* calcd for C_22_H_25_^35^ClN_2_O_2_ + H^+^: 385.1677 [M + H]^+^; found: 385.1679.

#### (+)-(7*R*,11*R*)-5-[(3-Chloro-6,7,10,11-tetrahydro-9-methyl-7,11-methanocycloocta[*b*]quinolin-12-yl)amino]pentanoic Acid [(+)-(7*R*,11*R*)-**14**]

This compound was prepared as described for **11c**. From nitrile (+)-**13** (102 mg, 0.28 mmol), crude (+)-**14** (575 mg), in the form of quinoline hydrochloride salt, was obtained as a yellow solid that was used in the following step without further purification: The ^1^H NMR spectrum of (+)-**14** coincided with that of its enantiomer (−)-**14**.

#### 1-{1-{3-[(6-Chloro-1,2,3,4-tetrahydroacridin-9-yl)amino]propanoyl}piperidin-4-yl}-3-[4-(trifluoromethoxy)phenyl]urea (**12a**)

A suspension of crude **11a** (144 mg) in a mixture of EtOAc/DMF (7.7 mL, 10:1) was treated with EDC·HCl (121 mg, 0.63 mmol), Et_3_N (0.27 mL, 196 mg, 1.94 mmol), and HOBt (86 mg, 0.63 mmol), and the mixture was stirred at room temperature for 10 min. A solution of amine **6**·HCl (158 mg, 0.47 mmol) in EtOAc/DMF (8.8 mL, 10:1) was then added, and the reaction mixture was stirred at room temperature overnight and then evaporated to dryness to give a brown oil (708 mg), which was purified through column chromatography (35–70 μm silica gel, CH_2_Cl_2_/MeOH/50% aq NH_4_OH mixtures, gradient elution). On elution with CH_2_Cl_2_/MeOH/50% aq NH_4_OH 99:1:0.4, compound **12a** (113 mg, 44% overall yield from **10a**) was isolated as a yellowish solid: *R*_f_ 0.7 (CH_2_Cl_2_/MeOH/50% aq NH_4_OH 9.5:0.5:0.02).

The analytical sample of **12a**·HCl was obtained as follows. A solution of **12a** (69 mg) in CH_2_Cl_2_ (2 mL) was filtered through a 0.2 μm PTFE filter, treated with a solution of HCl in Et_2_O (1.17 M, 1 mL), and evaporated to dryness. The resulting solid was washed with EtOAc (2 × 2 mL), hexane (2 × 2 mL), and pentane (2 × 2 mL) and dried at 45 °C/2 Torr for 5 days to provide **12a**·HCl (65 mg) as a yellowish solid: mp 191–192 °C; ^1^H NMR (400 MHz, CD_3_OD) δ (ppm): 1.38 (m, 1H, piperidine 3-H_A_), 1.47 (m, 1H, piperidine 5-H_A_), 1.90–2.00 (m, 5H, 2′-H_2_, 3′-H_2_, piperidine 3-H_B_), 2.03 (dm, *J* = 14.0 Hz, 1H, piperidine 5-H_B_), 2.67 (m, 2H, 1′-H_2_), overimposed in part 2.92 (m, 1H, piperidine 2-H_A_), 2.99 (m, 2H, 4′-H_2_), 3.00 (t, *J* = 6.0 Hz, 2H, 2-H_2_), 3.27 (ddd, *J* = 14.0 Hz, *J*′ = 11.2 Hz, *J*″ = 2.8 Hz, 1H, piperidine 6-H_A_), 3.83 (dddd, *J* = *J*′ = 10.4 Hz, *J*″ = *J*‴ = 4.0 Hz, 1H, piperidine 4-H), 3.93 (dm, *J* = 14.0 Hz, 1H, piperidine 6-H_B_), 4.23 (t, *J* = 6.0 Hz, 2H, 3-H_2_), 4.37 (dm, *J* = 14.0 Hz, 1H, piperidine 2-H_B_), 4.85 (s, NH, ^+^NH), 7.15 [br d, *J* = 9.2 Hz, 2H, phenyl 2(6)-H], 7.44 [dm, *J* = 9.2 Hz, 2H, phenyl 3(5)-H], 7.57 (dd, *J* = 9.2 Hz, *J*′ = 2.0 Hz, 1H, 7′-H), 7.78 (d, *J* = 2.0 Hz, 1H, 5′-H), 8.39 (d, *J* = 9.2 Hz, 1H, 8′-H); ^13^C NMR (100.6 MHz, CD_3_OD) δ (ppm): 21.8 (CH_2_, C3′), 22.8 (CH_2_, C2′), 24.6 (CH_2_, C1′), 29.3 (CH_2_, C4′), 33.0 (CH_2_), 33.6 (CH_2_) (piperidine C3 and C5), 33.7 (CH_2_, C2), 41.7 (CH_2_, piperidine C2), 45.5 (CH_2_, piperidine C6), 45.7 (CH_2_, C3), 48.0 (CH, piperidine C4), 113.7 (C, C9a′), 115.6 (C, C8a′), 119.2 (CH, C5′), 120.9 [2CH, phenyl C3(5)], 122.0 (C, q, *J*_C–F_ = 255 Hz, CF_3_O), 122.6 [2CH, phenyl C2(6)], 126.8 (CH, C7′), 128.8 (CH, C8′), 140.11 (C), 140.15 (C) (C6′, phenyl C1), 140.5 (C, C10a′), 145.0 (C, q, *J*_C–F_ = 1.9 Hz, phenyl C4), 152.3 (C, C4a′), 157.2 (C, NHCONH), 158.0 (C, C9′), 171.1 (C, C1); IR (ATR) ν (cm^–1^): 3500–2500 (max at 3279, 3056, 2939, N–H, ^+^N–H, C–H st), 1683, 1634 (C=O st); HRMS (ESI): *m*/*z* calcd for C_29_H_31_^35^ClF_3_N_5_O_3_ + H^+^: 590.2140 [M + H]^+^; found: 590.2137; HPLC purity = 98.6%.

#### 1-{1-{4-[(6-Chloro-1,2,3,4-tetrahydroacridin-9-yl)amino]butanoyl}piperidin-4-yl}-3-[4-(trifluoromethoxy)phenyl]urea (**12b**)

This compound was prepared as described for **12a**. From crude **11b** (844 mg), EDC·HCl (140 mg, 0.73 mmol), Et_3_N (0.34 mL, 247 mg, 2.44 mmol), HOBt (99 mg, 0.73 mmol), and amine **6**·HCl (182 mg, 0.54 mmol), a brown oily residue (1.38 g) was obtained and subjected to column chromatography purification (35–70 μm silica gel, CH_2_Cl_2_/MeOH/50% aq NH_4_OH mixtures, gradient elution). On elution with CH_2_Cl_2_/MeOH/50% aq NH_4_OH 98:2:0.4, compound **12b** (132 mg, 61% overall yield from **10b**) was isolated as a yellowish solid: *R*_f_ 0.5 (CH_2_Cl_2_/MeOH/50% aq NH_4_OH 9.5:0.5:0.02).

The analytical sample of **12b**·HCl was prepared as described for **12a**·HCl. From **12b** (132 mg) and a solution of HCl in Et_2_O (1.17 M, 1 mL), **12b**·HCl (72 mg) was obtained as a yellowish solid: mp 195–197 °C; ^1^H NMR (400 MHz, CD_3_OD) δ (ppm): 1.35 (m, 1H, piperidine 3-H_A_), 1.41 (m, 1H, piperidine 5-H_A_), 1.90–2.00 (m, 5H, 2′-H_2_, 3′-H_2_, piperidine 3-H_B_), 2.02 (dm, *J* = 14.0 Hz, 1H, piperidine 5-H_B_), 2.15 (tt, *J* = 6.4 Hz, *J*′ = 6.0 Hz, 2H, 3-H_2_), 2.67 (t, *J* = 6.0 Hz, 2H, 2-H_2_), 2.71 (m, 2H, 1′-H_2_), overimposed in part 2.93 (ddd, *J* = 14.0 Hz, *J*′ = 12.4 Hz, *J*″ = 3.2 Hz, 1H, piperidine 2-H_A_), 2.99 (m, 2H, 4′-H_2_), 3.24 (ddd, *J* = 14.0 Hz, *J*′ = 11.2 Hz, *J*″ = 2.8 Hz, 1H, piperidine 6-H_A_), 3.82 (dddd, *J* = *J*′ = 10.4 Hz, *J*″ = *J*‴ = 4.4 Hz, 1H, piperidine 4-H), 3.92 (dm, *J* = 14.0 Hz, 1H, piperidine 6-H_B_), 4.02 (t, *J* = 6.4 Hz, 2H, 4-H_2_), 4.41 (dm, *J* = 14.0 Hz, 1H, piperidine 2-H_B_), 4.85 (s, NH, ^+^NH), 7.15 [br d, *J* = 8.8 Hz, 2H, phenyl 2(6)-H], 7.43 [dm, *J* = 8.8 Hz, 2H, phenyl 3(5)-H], 7.56 (dd, *J* = 9.2 Hz, *J*′ = 2.0 Hz, 1H, 7′-H), 7.75 (d, *J* = 2.0 Hz, 1H, 5′-H), 8.53 (d, *J* = 9.2 Hz, 1H, 8′-H); ^13^C NMR (100.6 MHz, CD_3_OD) δ (ppm): 21.8 (CH_2_, C3′), 22.9 (CH_2_, C2′), 24.9 (CH_2_, C1′), 26.1 (CH_2_, C3), 29.3 (CH_2_, C4′), 31.8 (CH_2_), 33.0 (CH_2_) (piperidine C3 and C5), 33.7 (CH_2_, C2), 41.9 (CH_2_, piperidine C2), 45.4 (CH_2_, piperidine C6), 48.0 (CH, piperidine C4), overimposed with solvent signal 49.0 (CH_2_, C4), 113.3 (C, C9a′), 115.4 (C, C8a′), 119.0 (CH, C5′), 120.9 [2CH, phenyl C3(5)], 122.0 (C, q, *J*_C–F_ = 254 Hz, CF_3_O), 122.6 [2CH, phenyl C2(6)], 126.7 (CH, C7′), 129.1 (CH, C8′), 140.06 (C), 140.09 (C) (C6′, phenyl C1), 140.6 (C, C10a′), 145.0 (C, q, *J*_C–F_ = 2.0 Hz, phenyl C4), 151.8 (C, C4a′), 157.2 (C, NHCONH), 157.8 (C, C9′), 173.1 (C, C1); IR (ATR) ν (cm^–1^): 3500–2500 (max at 3268, 3055, 2938, N–H, ^+^N–H, C–H st), 1683, 1635 (C=O st); HRMS (ESI): *m*/*z* calcd for C_30_H_33_^35^ClF_3_N_5_O_3_ + H^+^: 604.2297 [M + H]^+^; found: 604.2287; HPLC purity = 97.8%.

#### 1-{1-{5-[(6-Chloro-1,2,3,4-tetrahydroacridin-9-yl)amino]pentanoyl}piperidin-4-yl}-3-[4-(trifluoromethoxy)phenyl]urea (**12c**)

This compound was prepared as described for **12a**. From crude **11c** (789 mg), EDC·HCl (265 mg, 1.38 mmol), Et_3_N (0.64 mL, 465 mg, 4.59 mmol), HOBt (188 mg, 1.39 mmol), and amine **6**·HCl (313 mg, 0.92 mmol), a brown oily residue (2.09 g) was obtained and subjected to column chromatography purification (35–70 μm silica gel, CH_2_Cl_2_/MeOH/50% aq NH_4_OH mixtures, gradient elution). On elution with CH_2_Cl_2_/MeOH/50% aq NH_4_OH 98.5:1.5:0.4, compound **12c** (452 mg, 79% overall yield from **10c**) was isolated as an ochre solid: *R*_f_ 0.22 (CH_2_Cl_2_/MeOH/50% aq NH_4_OH 9.6:0.4:0.04).

The analytical sample of **12c**·HCl was prepared as described for **12a**·HCl. From **12c** (452 mg) and a solution of HCl in Et_2_O (1.17 M, 2 mL), **12c**·HCl (411 mg) was obtained as a light brown solid: mp 154–157 °C; ^1^H NMR (400 MHz, CD_3_OD) δ (ppm): 1.30–1.47 (m, 2H, piperidine 3-H_A_ and 5-H_A_), 1.73 (tt, *J* = *J*′ = 7.2 Hz, 2H, 3-H_2_), 1.87 (tt, *J* = *J*′ = 7.2 Hz, 2H, 4-H_2_), 1.90–2.05 (m, 6H, 2′-H_2_, 3′-H_2_, piperidine 3-H_B_ and 5-H_B_), 2.49 (td, *J* = 7.2 Hz, *J*′ = 4.0 Hz, 2H, 2-H_2_), 2.71 (m, 2H, 1′-H_2_), 2.90 (ddd, *J* = 14.0 Hz, *J*′ = 12.4 Hz, *J*″ = 2.8 Hz, 1H, piperidine 2-H_A_), 3.00 (m, 2H, 4′-H_2_), 3.22 (ddd, *J* = 14.0 Hz, *J*′ = 12.4 Hz, *J*″ = 2.8 Hz, 1H, piperidine 6-H_A_), 3.81 (dddd, *J* = *J*′ = 10.8 Hz, *J*″ = *J*‴ = 4.4 Hz, 1H, piperidine 4-H), 3.91 (dm, *J* = 14.0 Hz, 1H, piperidine 6-H_B_), 3.98 (t, *J* = 7.2 Hz, 2H, 5-H_2_), 4.37 (dm, *J* = 14.0 Hz, 1H, piperidine 2-H_B_), 4.85 (s, NH, ^+^NH), 7.15 [br d, *J* = 9.2 Hz, 2H, phenyl 2(6)-H], 7.43 [dm, *J* = 9.2 Hz, 2H, phenyl 3(5)-H], 7.56 (dd, *J* = 9.2 Hz, *J*′ = 2.0 Hz, 1H, 7′-H), 7.76 (d, *J* = 2.0 Hz, 1H, 5′-H), 8.43 (d, *J* = 9.2 Hz, 1H, 8′-H); ^13^C NMR (100.6 MHz, CD_3_OD) δ (ppm): 21.8 (CH_2_, C3′), 22.9 (CH_2_, C2′), 23.2 (CH_2_, C3), 24.8 (CH_2_, C1′), 29.3 (CH_2_, C4′), 30.8 (CH_2_, C4), 33.0 (CH_2_, piperidine C3), 33.1 (CH_2_, C2), 33.9 (CH_2_, piperidine C5), 41.7 (CH_2_, piperidine C2), 45.5 (CH_2_, piperidine C6), 48.1 (CH, piperidine C4), 48.9 (CH_2_, C5), 113.4 (C, C9a′), 115.5 (C, C8a′), 119.1 (CH, C5′), 120.8 [2CH, phenyl C3(5)], 122.0 (C, q, *J*_C–F_ = 254 Hz, CF_3_O), 122.6 [2CH, phenyl C2(6)], 126.8 (CH, C7′), 128.8 (CH, C8′), 140.10 (C), 140.15 (C) (C6′, phenyl C1), 140.5 (C, C10a′), 145.0 (C, q, *J*_C–F_ = 2.2 Hz, phenyl C4), 152.1 (C, C4a′), 157.2 (C, NHCONH), 157.9 (C, C9′), 173.3 (C, C1); IR (ATR) ν (cm^–1^): 3500–2500 (max at 3278, 3062, 2936, N–H, ^+^N–H, C–H st), 1688, 1631 (C=O st); HRMS (ESI): *m*/*z* calcd for C_31_H_35_^35^ClF_3_N_5_O_3_ + H^+^: 618.2453 [M + H]^+^; found: 618.2443; HPLC purity = 98.2%.

#### (−)-(7*S*,11*S*)-1-{1-{5-[(3-Chloro-6,7,10,11-tetrahydro-9-methyl-7,11-methanocycloocta[*b*]quinolin-12-yl)amino]pentanoyl}piperidin-4-yl}-3-[4-(trifluoromethoxy)phenyl]urea [(−)-(7*S*,11*S*)-**15**]

This compound was prepared as described for **12a**. From crude (−)-(7*S*,11*S*)-**14** (1.55 g), EDC·HCl (161 mg, 0.84 mmol), Et_3_N (0.39 mL, 283 mg, 2.80 mmol), HOBt (114 mg, 0.84 mmol), and amine **6**·HCl (190 mg, 0.56 mmol), a brown oily residue (2.28 g) was obtained and subjected to column chromatography purification (35–70 μm silica gel, CH_2_Cl_2_/MeOH/50% aq NH_4_OH mixtures, gradient elution). On elution with CH_2_Cl_2_/MeOH/50% aq NH_4_OH 98.5:1.5:0.4, compound (−)-**15** [286 mg, 76% overall yield from (−)-**13**] was isolated as a light brown solid: *R*_f_ 0.67 (CH_2_Cl_2_/MeOH/50% aq NH_4_OH 9.5:0.5:0.04).

The analytical sample of (−)-**15**·HCl was prepared as described for **12a**·HCl. From (−)-**15** (286 mg) and a solution of HCl in Et_2_O (1.17 M, 2 mL), (−)-**15**·HCl (234 mg) was obtained as a gray solid: mp 177–180 °C; [α]^20^_D_ = −145 (*c* 0.60 in MeOH); ^1^H NMR (400 MHz, CD_3_OD) δ (ppm): 1.28–1.48 (m, 2H, piperidine 3-H_A_ and 5-H_A_), 1.58 (s, 3H, 9′-CH_3_), 1.75 (tt, *J* = *J*′ = 7.2 Hz, 2H, 3-H_2_), 1.85–2.10 (m, 7H, 4-H_2_, 10′-H_*endo*_, 13′-H_*syn*_, 13′-H_*anti*_, piperidine 3-H_B_ and 5-H_B_), 2.51 (t, *J* = 7.2 Hz, 2H, 2-H_2_), 2.57 (dd, *J* = 18.0 Hz, *J*′ = 5.2 Hz, 1H, 10′-H_*exo*_), 2.77 (m, 1H, 7′-H), 2.86 (br d, *J* = 18.0 Hz, 1H, 6′-H_*endo*_), overimposed in part 2.94 (ddd, *J* = 14.0 Hz, *J*′ = 11.2 Hz, *J*″ = 2.8 Hz, 1H, piperidine 2-H_A_), 3.20 (dd, *J* = 18.0 Hz, *J*′ = 5.6 Hz, 1H, 6′-H_*exo*_), overimposed in part 3.23 (ddd, *J* = 14.0 Hz, *J*′ = 11.2 Hz, *J*″ = 2.4 Hz, 1H, piperidine 6-H_A_), 3.51 (m, 1H, 11′-H), 3.82 (dddd, *J* = *J*′ = 10.4 Hz, *J*″ = *J*‴ = 4.0 Hz, 1H, piperidine 4-H), 3.92 (dm, *J* = 14.0 Hz, 1H, piperidine 6-H_B_), 4.01 (t, *J* = 6.8 Hz, 2H, 5-H_2_), 4.36 (dm, *J* = 14.0 Hz, 1H, piperidine 2-H_B_), 4.85 (s, NH, ^+^NH), 5.58 (br d, *J* = 5.2 Hz, 1H, 8′-H), 7.14 [br d, *J* = 9.2 Hz, 2H, phenyl 2(6)-H], 7.44 [dm, *J* = 9.2 Hz, 2H, phenyl 3(5)-H], 7.56 (dd, *J* = 9.2 Hz, *J*′ = 2.0 Hz, 1H, 2′-H), 7.76 (d, *J* = 2.0 Hz, 1H, 4′-H), 8.43 (d, *J* = 9.2 Hz, 1H, 1′-H); ^13^C NMR (100.6 MHz, CD_3_OD) δ (ppm): 23.2 (CH_2_, C3), 23.5 (CH_3_, 9′-CH_3_), 27.3 (CH, C11′), 27.9 (CH, C7′), 29.3 (CH_2_, C13′), 30.8 (CH_2_, C4), 33.06 (CH_2_, piperidine C3), 33.10 (CH_2_, C2), 33.9 (CH_2_, piperidine C5), 36.0 (CH_2_, C6′), 36.2 (CH_2_, C10′), 41.8 (CH_2_, piperidine C2), 45.4 (CH_2_, piperidine C6), 48.1 (CH, piperidine C4), 49.3 (CH_2_, C5), 115.7 (C, C12a′), 117.7 (C, C11a′), 119.1 (CH, C4′), 120.9 [2CH, phenyl C3(5)], 122.0 (C, q, *J*_C–F_ = 254 Hz, CF_3_O), 122.6 [2CH, phenyl C2(6)], 125.1 (CH, C8′), 126.7 (CH, C2′), 129.6 (CH, C1′), 134.6 (C, C9′), 140.1 (C), 140.2 (C) (C3′, phenyl C1), 141.0 (C, C4a′), 145.0 (C, q, *J*_C–F_ = 2.1 Hz, phenyl C4), 151.2 (C, C5a′), 157.0 (C, C12′), 157.2 (C, NHCONH), 173.2 (C, C1); IR (ATR) ν (cm^–1^): 3500–2500 (max at 3258, 3060, 2933, N–H, ^+^N–H, C–H st), 1682, 1633 (C=O st); HRMS (ESI): *m*/*z* calcd for C_35_H_39_^35^ClF_3_N_5_O_3_ + H^+^: 670.2766 [M + H]^+^; found: 670.2762; HPLC purity = 98.5%.

#### (+)-(7*R*,11*R*)-1-{1-{5-[(3-Chloro-6,7,10,11-tetrahydro-9-methyl-7,11-methanocycloocta[*b*]quinolin-12-yl)amino]pentanoyl}piperidin-4-yl}-3-[4-(trifluoromethoxy)phenyl]urea [(+)-(7*R*,11*R*)-**15**]

This compound was prepared as described for **12a**. From crude (+)-(7*R*,11*R*)-**14** (575 mg), EDC·HCl (80 mg, 0.42 mmol), Et_3_N (0.18 mL, 131 mg, 1.29 mmol), HOBt (57 mg, 0.42 mmol), and amine **6**·HCl (104 mg, 0.31 mmol), a brown oily residue (983 mg) was obtained and subjected to column chromatography purification (35–70 μm silica gel, CH_2_Cl_2_/MeOH/50% aq NH_4_OH mixtures, gradient elution). On elution with CH_2_Cl_2_/MeOH/50% aq NH_4_OH 99:1:0.4 to 98:2:0.4, compound (+)-**15** [74 mg, 40% overall yield from (+)-**13**] was isolated as a light brown solid: *R*_f_ 0.68 (CH_2_Cl_2_/MeOH/50% aq NH_4_OH 9.5:0.5:0.04).

The analytical sample of (+)-**15**·HCl was prepared as described for **12a**·HCl. From (+)-**15** (74 mg) and a solution of HCl in Et_2_O (1.17 M, 1 mL), (+)-**15**·HCl (58 mg) was obtained as a light brown solid: mp 178–180 °C; [α]^20^_D_ = +138 (*c* 0.54 in MeOH); ^1^H NMR (400 MHz, CD_3_OD) δ (ppm): 1.28–1.48 (m, 2H, piperidine 3-H_A_ and 5-H_A_), 1.58 (s, 3H, 9′-CH_3_), 1.75 (tt, *J* = *J*′ = 7.2 Hz, 2H, 3-H_2_), 1.85–2.10 (m, 7H, 4-H_2_, 10′-H_*endo*_, 13′-H_*syn*_, 13′-H_*anti*_, piperidine 3-H_B_ and 5-H_B_), 2.51 (t, *J* = 7.2 Hz, 2H, 2-H_2_), 2.57 (dd, *J* = 18.0 Hz, *J*′ = 5.6 Hz, 1H, 10′-H_*exo*_), 2.77 (m, 1H, 7′-H), 2.86 (br d, *J* = 18.0 Hz, 1H, 6′-H_*endo*_), overimposed in part 2.91 (m, 1H, piperidine 2-H_A_), 3.20 (dd, *J* = 18.0 Hz, *J*′ = 5.6 Hz, 1H, 6′-H_*exo*_), overimposed in part 3.23 (ddd, *J* = 14.0 Hz, *J*′ = 10.8 Hz, *J*″ = 2.8 Hz, 1H, piperidine 6-H_A_), 3.50 (m, 1H, 11′-H), 3.82 (dddd, *J* = *J*′ = 10.4 Hz, *J*″ = *J*‴ = 3.6 Hz, 1H, piperidine 4-H), 3.91 (dm, *J* = 14.0 Hz, 1H, piperidine 6-H_B_), 4.01 (t, *J* = 7.2 Hz, 2H, 5-H_2_), 4.36 (dm, *J* = 13.6 Hz, 1H, piperidine 2-H_B_), 4.85 (s, NH, ^+^NH), 5.58 (br d, *J* = 5.6 Hz, 1H, 8′-H), 7.14 [br d, *J* = 9.2 Hz, 2H, phenyl 2(6)-H], 7.44 [dm, *J* = 9.2 Hz, 2H, phenyl 3(5)-H], 7.56 (dd, *J* = 9.2 Hz, *J*′ = 2.0 Hz, 1H, 2′-H), 7.75 (d, *J* = 2.0 Hz, 1H, 4′-H), 8.43 (d, *J* = 9.2 Hz, 1H, 1′-H); ^13^C NMR (100.6 MHz, CD_3_OD) δ (ppm): 23.2 (CH_2_, C3), 23.5 (CH_3_, 9′-CH_3_), 27.3 (CH, C11′), 27.9 (CH, C7′), 29.3 (CH_2_, C13′), 30.8 (CH_2_, C4), 33.06 (CH_2_, piperidine C3), 33.10 (CH_2_, C2), 33.9 (CH_2_, piperidine C5), 36.0 (CH_2_, C6′), 36.2 (CH_2_, C10′), 41.8 (CH_2_, piperidine C2), 45.4 (CH_2_, piperidine C6), 48.1 (CH, piperidine C4), 49.3 (CH_2_, C5), 115.7 (C, C12a′), 117.7 (C, C11a′), 119.1 (CH, C4′), 120.9 [2CH, phenyl C3(5)], 122.0 (C, q, *J*_C–F_ = 255 Hz, CF_3_O), 122.6 [2CH, phenyl C2(6)], 125.1 (CH, C8′), 126.7 (CH, C2′), 129.6 (CH, C1′), 134.6 (C, C9′), 140.1 (C), 140.2 (C) (C3′, phenyl C1), 141.0 (C, C4a′), 145.0 (C, phenyl C4), 151.2 (C, C5a′), 157.0 (C, C12′), 157.2 (C, NHCONH), 173.2 (C, C1); IR (ATR) ν (cm^–1^): 3500–2500 (max at 3272, 3073, 2935, N–H, ^+^N–H, C–H st), 1689, 1632 (C=O st); HRMS (ESI): *m*/*z* calcd for C_35_H_39_^35^ClF_3_N_5_O_3_ + H^+^: 670.2766 [M + H]^+^; found: 670.2762; HPLC purity = 98.2%.

### Biological Methods

The assays for the in vitro determination of the inhibitory activities toward human and mouse soluble epoxide hydrolase,^37,59^ human and mouse acetylcholinesterase,^60^ and human butyrylcholinesterase,^60^ the propidium displacement studies,^61−63^ the assays for determination of the PAMPA-BBB permeability,^47^ aqueous solubility, cytotoxicity in SH-SY5Y cells, and microsomal stability, and the in vivo efficacy studies in SAMP8 mice^64−66^ were carried out following described methodologies previously used in our group (see the Supporting Information for complete details and Tables S2–S6).^10,67,68^

Mice were treated according to European Community Council Directive 86/609/EEC and the studies were approved by the Institutional Animal Care and Use Committee of the University of Barcelona (670/14/8102) and by Generalitat de Catalunya, Spain (10291). All studies and procedures for the behavioral tests, brain dissection, and extractions followed the ARRIVE. Every effort was made to minimize animal suffering and to reduce the number of animals.

### Computational Methods

#### Molecular Modeling General Setup

Compound **12c** and donepezil, as a reference ligand, were built using the Molecular Operating Environment software package.^69^ Considering a physiological pH of 7.4, the pyridine nitrogen of the tetrahydroacridine ring of **12c** and the piperidine nitrogen of donepezil were modeled positively charged. Models for AChE were built starting from the crystallographic structures, downloaded from the Protein Data Bank,^70^ of hAChE^71^ in complex with 9-aminoacridine (PDB ID: 6O4X) and donepezil (PDB ID: 6O4W). Standard protein preparation protocols were followed, including the removal of co-crystallized ligands and crystallization buffer compounds and salts. As in previous works,^72−74^ hAChE was modeled in the physiological form. Specifically, His447 (according to the FASTA numbering of the protein excluding the N-terminal signal peptide) at the catalytic site was modeled as the δ-tautomer, while Glu450 was modeled in the protonated neutral state. The remaining residues were modeled as predicted by PROPKA^75^ for a pH of 7.4. Three disulfide bonds were included between residues 69–96, 257–272, and 409–529. The rotamer of Trp286 was adjusted manually to reproduce different orientations observed in different crystallographic complexes with different AChE inhibitors (vide infra).

Models for sEH were built using the crystallographic structure of sEH in complex with TPPU (PDB ID: 4OD0). Co-crystallized ligands and ions were removed from the structure. Amino acid protonation states were predicted using the H++ server (http://biophysics.cs.vt.edu/H++). As used in previous works, the sEH initial structure was prepared with the following protonation of histidine residues: HIE146, HIE239, HIP251, HID265, HIP334, HIE420, HIE506, HIE513, HIE518, and HIP524.^68^

#### Docking Calculations

Docking calculations were carried out with rDock.^76,77^ Superimposition of crystal structures of AChE in complex with different ligands (PDB IDs 6O4X,^71^1EVE,^78^1Q83,^79^ and 2CKM(80)) revealed that seven water molecules were conserved in the CAS region, with four establishing relevant interactions with aminoacridine-like molecules. Therefore, these four water molecules (with 6O4X numbering 724, 751, 756, and 784) were retained during docking calculations. To account for the plasticity of the PAS region, three different receptor structures were set up, differing in the rotamer of Trp286: The first model, hereafter referred to as AChE_6O4X_, was modeled with Trp286 in the c_1_ −60° and c_2_ −80° rotamer. In the second model, AChE_1Q83_, Trp286 was modeled in the c_1_ −120° and c_2_ +50° rotamer, and in the last model, AChE_2CKM_, Trp286 was modeled in the c_1_ −160° and c_2_ +120° rotamer (additional details are provided in Table S7 of the Supporting Information). The cavity was defined using the reference ligand method as implemented in rDock using an artificial ligand that combined the molecular features of donepezil (6O4W),^71^ bis(7)-tacrine (2CKM),^80^ and syn-TZ2PA6 (1Q83).^79^ The tolerance from the reference was set to a radius of 9 Å, while the small sphere probe was set to 1.5 Å to maintain the cavity compactness. Each cavity had a volume of approximately 3500 Å^3^. The genetic algorithm of rDock was run 100 times, and the results were ranked according to the desolvation scoring function as implemented in the software. Through docking calculations, the ligand had full rotational and translational freedom, while the protein was kept rigid (with the exception of hydroxyl groups that were allowed to rotate). Water molecules were allowed full rotational freedom, while translational movements were constrained to a sphere of 1.75 Å, setting the occupancy parameter at 0.8.

For the docking calculations of **12c** in the sEH active site, three water molecules that occupy the sEH active site were retained (with 4OD0 numbering 702, 710, and 712). The cavity was defined using the reference ligand method as implemented in rDock, using TPPU as a reference ligand (4OD0).^81^ The cavity had a volume of approximately 1400 Å^3^. The same protocols as described above for AChE were used for the docking calculations of **12c** in the sEH active site. The binding pose presenting a better overlay with the TPPU unit of PDB: 4OD0 was selected for MD simulations.

#### Molecular Dynamics Simulations

The putative binding modes identified by means of docking calculations were further refined using molecular dynamics (MD) simulations carried out with the AMBER molecular simulation package.^82^ The ff14SB^83^ and gaff2^84^ force fields were used to assign atom types for the protein and the inhibitors, respectively. Partial charges for compound **12c** and donepezil were derived using the AM1-bcc^85,86^ approach as implemented in the antechamber. Each system, consisting of the protein, the inhibitor, and the structural waters present in the crystallographic structure 6O4X (after deleting those clashing with the inhibitor on the active site of the enzyme), was solvated on a truncated octahedral box of TIP3P^87^ water molecules, and seven Na^+^ counterions were added to achieve charge neutrality, accounting for simulation systems of approximately 50,000 atoms. Each system was then minimized in three stages: First, the position of water molecules was minimized combining 3500 steps of steepest descent and 6500 steps of conjugate gradient, while the position of the protein and ligand atoms was restrained using a harmonic potential with a force constant of 5.0 kcal mol^–1^ Å^–2^. Next, side chains and water molecules were minimized using 4500 steps of steepest descent followed by 7500 steps of conjugate gradient while the atoms of the ligand and the peptidic backbone were restrained with a harmonic potential using the same force constant. During the last minimization stage, all restraints were removed and the whole system was minimized for additional 4500 steps of steepest descent followed by 7500 steps of conjugate gradient.

At this point, three independent replicates were set up for all the systems, for a total of 24 independent MD complexes (7 × AChE–**12c** and 3 × AChE–donepezil complexes). Prior to the production runs, each of these complexes was first heated in three stages of 125 ps (50–150, 150–250, and 250–298 K) in the NVT ensemble, and subsequently, its density equilibrated at 1 bar for 250 ps in the NPT ensemble. Production runs consisted of 1 μs trajectories in the NPT ensemble at 298 K and 1 bar. Throughout the MD stages, temperature control was achieved using a Langevin thermostat (with a collision frequency of 3 ps^–1^) and a Monte Carlo barostat. SHAKE^88,89^ was applied to all atoms involving hydrogen to allow for a time step of 2 fs. All simulations were performed with the CUDA accelerated version of PMEMD.^90,91^

For sEH, MD simulations starting from the docking predicted pose were used to explore the conformational plasticity of sEH in the presence of **12c**. The same force fields used for simulating the AChE–**12c** complex were used for sEH–**12c**. Each system was immersed in a pre-equilibrated truncated octahedral box of water molecules with an internal offset distance of 10 Å. All systems were neutralized with explicit counterions (Na^+^ or Cl^–^). A two-stage geometry optimization approach was performed. First, a short minimization of the positions of water molecules with positional restraints on the solute by a harmonic potential with a force constant of 500 kcal mol^–1^ Å^–2^ was done. The second stage was an unrestrained minimization of all the atoms in the simulation cell. Then, the systems were gently heated in six 50 ps steps, increasing the temperature by 50 K each step (0–300 K) under constant-volume, periodic-boundary conditions, and the particle-mesh Ewald approach^92^ to introduce long-range electrostatic effects. For these steps, a 10 Å cutoff was applied to Lennard-Jones and electrostatic interactions. Bonds involving hydrogen were constrained with the SHAKE algorithm.^93^ Harmonic restraints of 10 kcal mol^–1^ were applied to the solute, and the Langevin equilibration scheme was used to control and equalize the temperature.^94^ The time step was kept at 2 fs during the heating stages, allowing potential inhomogeneities to self-adjust. Each system was then equilibrated for 2 ns with a 2 fs time step at a constant pressure of 1 atm (NPT ensemble). Finally, conventional MD trajectories at a constant volume and temperature (300 K) were collected. In total, there were three replicas of 250 ns MD simulations for sEH in the presence of compound **12c** (i.e., an accumulated MD simulation time of 750 ns). All MD simulations of **12c** were clusterized based on active site residues (considering all heavy atoms) using the clustering function of the cpptraj MD analysis program.^95^ The orientation of the 6-chlorotacrine moiety was explored for the 10 clusters obtained. The most populated cluster was selected for the molecular interaction analysis. Relevant average distances (in Å) were calculated considering the three replicas of 250 ns MD simulations.

## Supporting Information Available

Synthetic procedures for the preparation of the intermediates **5**, **6**, **8a**,**b**, **9a**,**b**, **10b**,**c**, and **11b**, complete details of in vitro and in vivo biological methods, best scoring poses, interaction patterns and RMSD values in simulations of **12c** in complex with AChE, binding mode of donepezil in control simulations, experimental timeline for in vivo studies, PDB AChE structures used in the analysis of prior knowledge of the cryptic pocket in the PAS, PAMPA-BBB permeabilities of commercial drugs used for assay validation, quantities of reagents, microsomes, and test compounds and UPLC-MS/MS analysis conditions used in the microsomal stability assays, antibodies and primers used in western blot and qPCR studies, additional data on AChE Trp286 rotamers, and copies of ^1^H and ^13^C NMR spectra and HPLC traces of the target compounds (PDF)
PDB coordinate files for computational models of complexes of **12c** with AChE and sEH (PDB)
Molecular formula strings (CSV)
Movie on the opening of the cryptic pocket in the **12c**–AChE complex (MPG)

## Supplementary Material

jm1c02150_si_003.csv

jm1c02150_si_004.mpg

## Dedication

In memoriam of Prof. Dr. Antonio Delgado.

## Abbreviations

ACh: acetylcholine
AChE: acetylcholinesterase
AD: Alzheimer’s disease
APP: amyloid precursor protein
BBB: blood–brain barrier
BChE: butyrylcholinesterase
CAS: catalytic anionic site
CL_int_: intrinsic clearance
CNS: central nervous system
DI: discrimination index
EETs: epoxyeicosatrienoic acids
GADPH: glyceraldehyde-3-phosphate dehydrogenase
*Gfap*: glial fibrillary acidic protein
hAChE: human acetylcholinesterase
hBChE: human butyrylcholinesterase
hsEH: human soluble epoxide hydrolase
Il-1β: interleukin 1β
Il-6: interleukin 6
LHS: left-hand side
mAChE: mouse acetylcholinesterase
MD: molecular dynamics
msEH: mouse soluble epoxide hydrolase
NFkβ: nuclear factor kβ
NORT: novel object recognition test
PAMPA-BBB: parallel artificial membrane permeation assay for the blood–brain barrier
PAS: peripheral anionic site
PBS: phosphate-buffered saline
*P*_e_: permeability
PI: propidium iodide
qPCR: real-time quantitative polymerase chain reaction
RHS: right-hand side
RMSD: root mean square deviation
RT-PCR: reverse transcription-polymerase chain reaction
SAMP8: senescence-accelerated mouse-prone 8
SDS-PAGE: sodium dodecyl sulfate-polyacrylamide gel electrophoresis
sEH: soluble epoxide hydrolase
SYN: synaptophysin
*t*_1/2_: half-life
TBS: TRIS-buffered saline
TBS-T: TRIS-buffered saline containing 0.1% Tween 20
