# Supplementary material for: Discovery and In Vivo Proof of Concept of a Highly Potent Dual Inhibitor of Soluble Epoxide Hydrolase and Acetylcholinesterase for the Treatment of Alzheimer’s Disease
Source: J Med Chem. 2022 Mar 10;65(6):4909–25. doi: 10.1021/acs.jmedchem.1c02150 (PMC8958510; doi:10.1021/acs.jmedchem.1c02150)
Supplement: Supplementary file 1 — jm1c02150_si_001.pdf [file jm1c02150_si_001.pdf]

SUPPORTING INFORMATION

Discovery and in Vivo Proof-of-Concept of a Highly Potent Dual Inhibitor of Soluble Epoxide Hydrolase and Acetylcholinesterase for the Treatment of Alzheimer's Disease

Sandra Codony,[1,‡] Caterina Pont,[1,‡] Christian Griñán-Ferré,[2] Ania Di Pede-Mattatelli,[3] Carla Calvó-Tusell,[4] Ferran Feixas,[4] Sílvia Osuna,[4,5] Júlia Jarné-Ferrer,[2] Marina Naldi,[6] Manuela Bartolini,[6] María Isabel Loza,[7] José Brea,[7] Belén Pérez,[8] Clara Bartra,[9] Coral Sanfeliu,[9] Jordi Juárez-Jiménez,[3] Christophe Morisseau,[10] Bruce D. Hammock,[10] Mercè Pallàs,[2] Santiago Vázquez,[1] Diego Muñoz-Torrero*[1]*

[1] Laboratory of Medicinal Chemistry (CSIC Associated Unit), Faculty of Pharmacy and Food Sciences, and Institute of Biomedicine (IBUB), University of Barcelona (UB), Av. Joan XXIII 27-31, E-08028, Barcelona, Spain

[2] Pharmacology Section, Department of Pharmacology, Toxicology and Therapeutic Chemistry, Faculty of Pharmacy and Food Sciences, and Institute of Neurosciences, UB, Av. Joan XXIII 27-31, E-08028, Barcelona, Spain

[3] Departament of Pharmacy and Pharmaceutical Technology and Physical Chemistry, Faculty of Pharmacy and Food Sciences, and Institute of Theoretical and Computational Chemistry (IQTCUB), UB, Av. Joan XXIII 27-31, E-08028, Barcelona, Spain

[4] CompBioLab Group, Departament de Química and Institut de Química Computacional i Catàlisi (IQCC), Universitat de Girona, C/ Maria Aurèlia Capmany 69, E-17003 Girona, Spain

[5] Institució Catalana de Recerca i Estudis Avançats (ICREA), E-08010 Barcelona, Spain

[6] Department of Pharmacy and Biotechnology, University of Bologna, Via Belmeloro, 6, I-40126 Bologna, Italy

[7] BioFarma Research Group, Centro Singular de Investigación en Medicina Molecular y Enfermedades Crónicas (CIMUS), Universidade de Santiago de Compostela, Av. de Barcelona s/n, E-15782, Santiago de Compostela, Spain

[8] Department of Pharmacology, Therapeutics and Toxicology, Autonomous University of Barcelona, E-08193 Bellaterra, Spain

[9] Institute of Biomedical Research of Barcelona, CSIC and Institut d'Investigacions Biomèdiques August Pi i Sunyer (IDIBAPS), Rosselló, 149, E-08036 Barcelona, Spain

[10] Department of Entomology and Nematology and Comprehensive Cancer Center, University of California, One Shields Avenue, Davis, CA 95616, USA

Corresponding authors' E-mail: svazquez@ub.edu (S.V.); dmunoztorrero@ub.edu (D.M-T.)

TABLE OF CONTENTS

Synthesis of intermediates **5**, **6**, **8a**,**b**, **9a**,**b**, **10b**,**c**, and **11b** S4

In vitro and in vivo biological methods S8

Figure S1. Best scoring poses for **12c** in each of the AChE models evaluated S17

Figure S2. Interactions of the 6-chlorotacrine moiety of **12c** in the CAS of AChE S17

Figure S3. RMSD values of the 6-chlorotacrine moiety of **12c** over the last 100 ns of each simulation S18

Figure S4. RMSD values of the TPPU moiety of **12c** over the last 100 ns of each simulation S18

Figure S5. Binding mode of donepezil in control simulations S19

Figure S6. In vivo treatment and experimental timeline S19

Figure S7. Familiarization phase of the NORT test S19

Table S1. Summary of the AChE structures available in the PDB that were analyzed to identify prior knowledge of the cryptic pocket in the PAS S20

Table S2. Reported and experimental PAMPA-BBB permeability of the commercial drugs used for assay validation S21

Table S3. Quantities of reagents, microsomes, and test compounds used in the microsomal stability assays S22

Table S4. Gradient used in the UPLC-MS/MS analysis for microsomal stability studies S22

Table S5. Antibodies used in Western Blot (WB) S23

Table S6. Primers and probes used in qPCR studies S23

Table S7. Reference c_1 and c_2 values for Trp286 rotamers in AChE S24

References S25

Copies of ^{1}H and ^{13}C NMR spectra and HPLC traces of target compounds S28

1-(1-Benzylpiperidin-4-yl)-3-[4-(trifluoromethoxy)phenyl]urea (5).[1] To a solution of 4-(trifluoromethoxy)phenyl isocyanate, **4** (800 mg, 3.94 mmol), in CH_2Cl_2 (4 mL), 1-benzylpiperidin-4-amine (900 mg, 4.73 mmol) was added. The reaction mixture was stirred at room temperature overnight. Then, the solvent was evaporated under vacuum to obtain a yellowish residue (2.52 g). After column chromatography purification (35-70 μm silica gel, CH_2Cl_2 / MeOH mixtures), urea **5** was isolated as a white solid (1.50 g, 97%): ^{1}H NMR (400 MHz, $CDCl_3$) δ (ppm): 1.45 [dddd, J = 12.8 Hz, J' = J'' = 11.2 Hz, J''' = 4.0 Hz, 2H, piperidine 3(5)-H_A], 1.93 [dm, J = 12.8 Hz, 2H, piperidine 3(5)-H_B], 2.10 [ddd, J = J' = 11.6 Hz, J'' = 2.8 Hz, 2H, piperidine 2(6)-H_A], 2.80 [br d, J = 11.6 Hz, 2H, piperidine 2(6)-H_B], 3.49 (s, 2H, benzyl CH_2), 3.68 (tdt, J = 11.2 Hz, J' = 8.0 Hz, J'' = 4.0 Hz, 1H, piperidine 4-H), 4.80 (d, J = 8.0 Hz, 1H, piperidine 4-NH), 6.70 (s, 1H, phenyl 1-NH), 7.12 [br d, J = 9.2 Hz, 2H, phenyl 2(6)-H], 7.22-7.34 [m, 7H, phenyl 3(5)-H, benzyl aromatic H].

1-(Piperidin-4-yl)-3-[4-(trifluoromethoxy)phenyl]urea (6).[2] To a solution of *N*-benzyl piperidine **5** (1.50 g, 3.81 mmol) in MeOH (60 mL), 10% Pd/C (150 mg) and concd. HCl (2 mL) were added, and the mixture was hydrogenated at atmospheric pressure for 5 days. The suspension was filtered and the resulting colorless solution was evaporated at reduced pressure. The residue was dissolved in CH_2Cl_2 (20 mL) and treated with 2 N NaOH until basic pH. The organic phase was separated and dried over anhydrous Na_2SO_4 and evaporated under vacuum, to afford piperidine **6** (953 mg, 82% yield) as a white solid: ^{1}H NMR (400 MHz, $CDCl_3$) δ (ppm): 1.31 [dddd, J = 12.4 Hz, J' = J'' = 11.2 Hz, J''' = 4.0 Hz, 2H, piperidine 3(5)-H_A], 1.97 [dm, J = 12.4 Hz, 2H, piperidine 3(5)-H_B], 2.68 [ddd, J = 12.4 Hz, J' = 11.6 Hz, J'' = 2.8 Hz, 2H, piperidine 2(6)-H_A], 3.06 [ddd, J = 12.4 Hz, J' = J'' = 3.6 Hz, 2H, piperidine 2(6)-H_B], 3.76 (tdt, J = 11.2 Hz, J' = 7.6 Hz, J'' = 4.0 Hz, 1H, piperidine 4-H), 4.76 (d, J = 7.6 Hz, 1H, piperidine 4-NH), 6.61 (s, 1H, phenyl 1-NH), 7.13 [dm, J = 9.2 Hz, 2H, phenyl 2(6)-H], 7.33 [dm, J = 9.2 Hz, 2H, phenyl 3(5)-H].

2-[(6-Chloro-1,2,3,4-tetrahydroacridin-9-yl)amino]ethanol (8a).[3] A solution of 6,9-dichloro-1,2,3,4-tetrahydroacridine, **7**[4] (1.00 g, 3.97 mmol) and 2-aminoethanol (0.72 mL, 729 mg, 11.9 mmol) in 1-pentanol (4 mL) was heated under reflux overnight and the resulting dark brown solution was cooled down to room temperature, was diluted with EtOAc (25 mL) and was extracted with 1 N HCl (4 × 15 mL). The combined aqueous layers were washed with EtOAc (3 × 20 mL), alkalinized with NaOH pellets (until pH = 12), and extracted with CH_2Cl_2 (3 × 25 mL). The combined organic extracts were dried over anhydrous Na_2SO_4 and evaporated to dryness to provide **8a** (1.03 g, 94% yield) as an ochre solid: ^{1}H NMR (400 MHz, CD_3OD) δ (ppm): 1.90-1.98 (complex signal, 4H, 2'-H_2, 3'-H_2), 2.78 (m, 2H, 1'-H_2), 2.98 (m, 2H, 4'-H_2), 3.64 (t, J = 5.2 Hz, 2H, 2-H_2), 3.72 (t, J = 5.2 Hz, 2H, 1-H_2), 4.85 (s, NH, OH), 7.34 (dd, J = 9.2 Hz, J' = 2.4 Hz, 1H, 7'-H), 7.75 (d, J = 2.4 Hz, 1H, 5'-H), 8.13 (d, J = 9.2 Hz, 1H, 8'-H).

3-[(6-Chloro-1,2,3,4-tetrahydroacridin-9-yl)amino]propanol (8b).[3] This compound was prepared as described for **8a**. From **7** (1.00 g, 3.97 mmol) and 3-amino-1-propanol (0.91 mL, 894 mg, 11.9 mmol), alcohol **8b** (817 mg, 71% yield) was obtained as a pale yellow solid: ^{1}H NMR (400 MHz, $CDCl_3$) δ (ppm): 1.83-1.88 (complex signal, 4H, 2'-H_2, 3'-H_2), 1.92 (tt, J = J' = 6.0 Hz, 2H, 2-H_2), 2.65 (m, 2H, 1'-H_2), 2.96 (m, 2H, 4'-H_2), 3.67 (dt, J = J' = 6.0 Hz, 2H, 3-H_2), 3.89 (t, J = 6.0 Hz, 2H, 1-H_2), 4.64 (br t, J = 6.0 Hz, 1H, NH), 7.21 (dd, J = 8.8 Hz, J' = 2.4 Hz, 1H, 7'-H), 7.83 (d, J = 2.4 Hz, 1H, 5'-H), 7.90 (d, J = 8.8 Hz, 1H, 8'-H).

2-[(6-Chloro-1,2,3,4-tetrahydroacridin-9-yl)amino]ethyl methanesulfonate (9a).[3] A suspension of alcohol **8a** (300 mg, 1.08 mmol) and anhydrous Et_3N (0.26 mL, 189 mg, 1.87 mmol) in dry CH_2Cl_2 (6 mL) was cooled to –10 °C in an ice / salt bath and methanesulfonyl chloride (0.13 mL, 192 mg, 1.68 mmol) was added dropwise. The reaction mixture was stirred at –10 °C for 30 min, concentrated in vacuo, taken up in CH_2Cl_2 (6 mL), and washed with 2 N NaOH (2 × 5 mL). The organic layer was dried over anhydrous Na_2SO_4 and

evaporated to dryness to provide mesylate **9a** (422 mg, quantitative yield), as a dark brown oil: ^{1}H NMR (400 MHz, $CDCl_3$) δ (ppm): 1.90-1.96 (complex signal, 4H, 2'-H_2, 3'-H_2), 2.76 (m, 2H, 1'-H_2), 3.00 (s, 3H, CH_3SO_3), 3.06 (m, 2H, 4'-H_2), 3.77 (dt, *J* = *J'* = 5.6 Hz, 2H, 2-H_2), 4.33 (t, *J* = 5.2 Hz, 2H, 1-H_2), 7.34 (dd, *J* = 8.8 Hz, *J'* = 2.0 Hz, 1H, 7'-H), 7.84 (d, *J* = 8.8 Hz, 1H, 8'-H), 7.92 (d, *J* = 2.0 Hz, 1H, 5'-H).

3-[(6-Chloro-1,2,3,4-tetrahydroacridin-9-yl)amino]propyl methanesulfonate (9b).[3] This compound was prepared as described for **9a**. From alcohol **8b** (426 mg, 1.46 mmol), anhydrous Et_3N (0.35 mL, 254 mg, 2.51 mmol) and methanesulfonyl chloride (0.17 mL, 252 mg, 2.20 mmol), mesylate **9b** (582 mg, quantitative yield) was obtained as a dark brown oil: ^{1}H NMR (400 MHz, $CDCl_3$) δ (ppm): 1.90-1.98 (complex signal, 4H, 2'-H_2, 3'-H_2), 2.11 (m, 2H, 2-H_2), 2.71 (m, 2H, 1'-H_2), 3.01 (s, 3H, CH_3SO_3), 3.05 (m, 2H, 4'-H_2), 3.61 (m, 1H, NH), 3.66 (t, *J* = 6.0 Hz, 2H, 3-H_2), 4.38 (t, *J* = 6.0 Hz, 2H, 1-H_2), 7.31 (dd, *J* = 8.8 Hz, *J'* = 2.4 Hz, 1H, 7'-H), 7.86 (d, *J* = 8.8 Hz, 1H, 8'-H), 7.90 (d, *J* = 2.4 Hz, 1H, 5'-H).

4-[(6-Chloro-1,2,3,4-tetrahydroacridin-9-yl)amino]butanenitrile (10b).[5] This compound was prepared as described for **10a**. From mesylate **9b** (539 mg, 1.46 mmol) and NaCN (358 mg, 7.30 mmol), nitrile **10b** (400 mg, 91% yield) was obtained, as a dark brown oil, without the need of column chromatography purification: R_f 0.3 (hexane / EtOAc / 50% aq. NH_4OH 6:4:0.02).

The analytical sample of **10b**·HCl was obtained as described for **10a**·HCl. From **10b** (39 mg) and a solution of HCl in Et_2O (1.17 M, 1 mL), **10b**·HCl (41 mg) was obtained as a foamy yellow solid: mp 201 °C (dec); ^{1}H NMR (400 MHz, CD_3OD) δ (ppm): 1.94-2.00 (complex signal, 4H, 2'-H_2, 3'-H_2), 2.18 (tt, *J* = *J'* = 7.2 Hz, 2H, 3-H_2), 2.63 (t, *J* = 7.2 Hz, 2H, 2-H_2), 2.72 (m, 2H, 1'-H_2), 3.03 (m, 2H, 4'-H_2), 4.08 (t, *J* = 7.2 Hz, 2H, 4-H_2), 4.85 (s, NH, $^+$NH), 7.57 (dd, *J* = 9.2 Hz, *J'* = 2.4 Hz, 1H, 7'-H), 7.81 (d, *J* = 2.4 Hz, 1H, 5'-H), 8.38 (d, *J* = 9.2 Hz, 1H, 8'-H); ^{13}C NMR (100.6 MHz, CD_3OD) δ (ppm): 15.0 (CH_2, C2), 21.7 (CH_2, C3'),

22.8 (CH_2, C2'), 24.9 (CH_2, C1'), 27.0 (CH_2, C3), 29.4 (CH_2, C4'), 47.7 (CH_2, C4), 113.9 (C, C9a'), 115.5 (C, C8a'), 119.2 (CH, C5'), 120.5 (C, C1), 127.0 (CH, C7'), 128.5 (CH, C8'), 140.1 (C, C6'), 140.4 (C, C10a'), 152.6 (C, C4a'), 157.9 (C, C9'); IR (ATR) ν (cm^{-1}): 3500-2400 (max at 3344, 3245, 2945, 2743, 2705, N–H, $^{+}$N–H, C–H st), 2247 (C≡N st); HRMS (ESI): *m/z* calcd for $C_{17}H_{18}{}^{35}ClN_3+H^+$: 300.1262 $[M+H]^+$; found: 300.1260.

5-[(6-Chloro-1,2,3,4-tetrahydroacridin-9-yl)amino]pentanenitrile (10c).[5] A mixture of 6-chlorotacrine hydrochloride, **2**·HCl (300 mg, 1.11 mmol), finely powdered KOH (85% purity reagent, 281 mg, 4.26 mmol) and 4Å molecular sieves in dry DMSO (4 mL) was stirred, heating every 10 min with a heat gun for 1 h, and at room temperature for an additional 1 h and then treated with a solution of 5-bromovaleronitrile (0.17 mL, 236 mg, 1.46 mmol) in dry DMSO (1 mL). The reaction mixture was stirred at room temperature overnight, then diluted with 5 N NaOH (30 mL) and extracted with EtOAc (3 × 20 mL). The combined organic layers were washed with water (3 × 30 mL) and brine (30 mL), dried over anhydrous Na_2SO_4 and evaporated to dryness to provide a brown oil (419 mg). Recrystallization from EtOAc (2.5 mL) afforded a white solid consisting of unreacted 6-chlorotacrine, with the mother liquors being enriched in the desired nitrile. After evaporation of the mother liquors at reduced pressure, the recrystallization process was repeated twice (EtOAc, 2 × 1 mL). Evaporation of the final mother liquors afforded pure **10c** (289 mg, 83% yield), as a dark yellow oil: R_f 0.73 (CH_2Cl_2 / MeOH / 50% aq. NH_4OH 9.5:0.5:0.02); ^{1}H NMR (400 MHz, $CDCl_3$) δ (ppm): 1.74-1.85 (m, 4H, 3-H_2, 4-H_2), 1.90-1.95 (m, 4H, 2'-H_2, 3'-H_2), 2.40 (t, J = 7.2 Hz, 2H, 2-H_2), 2.69 (m, 2H, 1'-H_2), 3.04 (m, 2H, 4'-H_2), 3.49 (t, J = 7.2 Hz, 2H, 5-H_2), 7.29 (dd, J = 9.2 Hz, J' = 2.4 Hz, 1H, 7'-H), 7.84 (d, J = 9.2 Hz, 1H, 8'-H), 7.90 (d, J = 2.4 Hz, 1H, 5'-H).

4-[(6-Chloro-1,2,3,4-tetrahydroacridin-9-yl)amino]butanoic acid (11b).[5] A suspension of nitrile **10b** (109 mg, 0.36 mmol) in 5 N HCl (9 mL) was heated under reflux for 3 h. The

resulting yellow solution was evaporated to dryness and the residue was taken up in water (15 mL), alkalinized with 10 N NaOH (until pH = 12), and washed with EtOAc (3 × 10 mL). The alkaline aqueous layer was evaporated to dryness, to afford crude carboxylic acid **11b** (844 mg), in the form of sodium carboxylate salt, as a pale yellow solid, which was used in the following step without further purification: ^{1}H NMR (400 MHz, CD_3OD) δ (ppm): 1.90-2.00 (complex signal, 4H, 2'-H_2, 3'-H_2), 2.12 (tt, $J = J'$ = 6.8 Hz, 2H, 3-H_2), 2.52 (t, J = 6.8 Hz, 2H, 2-H_2), 2.69 (m, 2H, 1'-H_2), 3.02 (m, 2H, 4'-H_2), 4.01 (dt, $J = J'$ = 6.8 Hz, 2H, 4-H_2), 4.85 (s, OH, NH, $^+$NH), 7.54 (dd, J = 9.2 Hz, J' = 2.4 Hz, 1H, 7'-H), 7.84 (d, J = 2.4 Hz, 1H, 5'-H), 8.49 (d, J = 9.2 Hz, 1H, 8'-H).

Human and mouse soluble epoxide hydrolase inhibition assay. The inhibitory activity of the target compounds toward human and mouse sEH was determined by a fluorescence-based assay, using purified recombinant human or mouse sEH proteins and CMNPC as a fluorescent substrate.[6] The enzymes were incubated at 30 °C with the inhibitors ($[I]_{final}$ = 0.4-100,000 nM) for 5 min in 100 mM sodium phosphate buffer (200 µL, pH 7.4) containing 0.1 mg mL^{-1} of BSA and 1% of DMSO. The substrate (CMNPC) was then added ($[S]_{final}$ = 5 µM). Activity was assessed by measuring the appearance of the fluorescent 6-methoxynaphthaldehyde product (λ_{ex} = 330 nm, λ_{em} = 465 nm) every 30 s for 10 min at 30 °C on a SpectraMax M2 (Molecular Devices). Results were obtained by regression analysis from a linear region of the curve and are given as IC_{50} values, i.e., the concentration of the compound that reduces the sEH activity by 50%. All measurements were performed in triplicate and the mean is reported. t-TUCB, a classic sEH inhibitor, was run in parallel and the obtained IC_{50}s were corroborated with reported literature values,[7] to validate the experimental results.

Human and mouse acetylcholinesterase and human butyrylcholinesterase inhibition assay. The inhibitory activity of the target compounds toward recombinant human AChE

(Sigma-Aldrich, Milan, Italy), recombinant mouse AChE (Bio-Techne, Milan, Italy) and human serum BChE (Sigma-Aldrich, Milan, Italy) was assessed by Ellman's method,[8] using **2**, (–)-**3**, and (+)-**3** as reference compounds. Stock solutions of the AChEs were prepared by dissolving enzyme lyophilized powder (hAChE) or diluting the enzyme commercial solution (mAChE) in 0.1 M potassium phosphate (pH 8.0) containing 0.1% Triton X-100, whereas the stock solution of hBChE was prepared in 0.1% aq. gelatin and the stock solutions of the compounds to be tested were made in MeOH (2-4 mM). The assay solution consisted of 0.02 unit mL^{-1} of the enzyme, 340 μM 5,5'-dithiobis(2-nitrobenzoic acid), and 550 μM of the substrate acetylthiocholine iodide (for AChE assay) or butyrylthiocholine iodide (for BChE assay) in 0.1 M potassium phosphate (pH 8.0). Blank solutions containing all components except the enzymes were prepared to account for the non-enzymatic hydrolysis of the substrate.

Prior the addition of the substrate, the assay solutions were incubated at 37 °C for 20 min. After the addition of the substrate, cuvettes were rapidly transferred into a Jasco V-530 double beam spectrophotometer equipped with thermostated cuvette holders (37 °C), in order to keep the assay temperature constant, and initial hydrolysis rates were monitored at 412 nm for 240 s. For each tested compound, five increasing concentrations were assayed in order to achieve inhibition percentages of 20–80%. Each concentration was assayed in triplicate. IC_{50} values were calculated from the inhibition plot (% inhibition vs log[inhibitor]) and are expressed as mean ± SEM. Each IC_{50} value is the average of at least two experiments, each performed in triplicate.

Propidium Displacement Studies. The affinity of **12c** for the PAS of *Electrophorus electricus* AChE (EeAChE) (type VI-S, Sigma-Aldrich) was determined using the PAS-specific ligand propidium iodide (P) (Sigma-Aldrich), following a described procedure.[9] Complexation of propidium iodide and AChE leads to a shift in the excitation wavelength.[9]

Fluorescence intensity was monitored with a Jasco 6200 spectrofluorometer (Jasco Europe, Italy) using a 0.5 mL quartz cuvette at rt. EeAChE (2 μM) was first incubated with 8 mM propidium iodide in 1 mM Tris-HCl, pH 8.0 at rt. The stock solution (8 mM) of **12c** was prepared in MeOH. In the back titration experiments of the propidium–AChE complex by **12c**, aliquots of the tested inhibitor (4–64 μM, final concentration) were added successively, and fluorescence emission was monitored at 635 nm upon excitation at 535 nm. Blanks containing propidium alone, **12c** plus propidium and EeAChE alone were prepared and fluorescence emission was determined. K_D values were obtained after processing the raw data following the method of Taylor and Lappi,[10] assuming a K_D = 0.7 μM for propidium.[11] K_D values are expressed as mean ± standard deviation (SD) of two independent experiments.

PAMPA-BBB assay for brain permeability. The brain permeability (P_e) of the target compounds was assessed by an in vitro parallel artificial membrane permeability assay for blood–brain barrier penetration (PAMPA-BBB assay),[12] which uses a lipid extract of porcine brain membrane as a BBB model. The assay was validated by comparing experimental and reported P_e values of a set of fourteen commercial drugs (Table S2 of the Supporting Information), and the following correlation was obtained: P_e (exp) = 1.6370 P_e (lit) – 1.3134 (R^2 = 0.9348). On the basis of this equation and the limits established by Di et al. for BBB permeation,[12] the threshold for high BBB permeation (CNS+) was set at P_e (10^{-6} cm s^{-1}) > 5.23; whereas the range for low BBB permeation (CNS–) was set at P_e (10^{-6} cm s^{-1}) < 1.96, and that for uncertain BBB permeation (CNS±) at 5.23 > P_e (10^{-6} cm s^{-1}) > 1.96. Three independent experiments, each performed in triplicate, were carried out for each compound.

Aqueous solubility screen. The kinetic aqueous solubility of the target compounds was determined at the Innopharma Drug Screening and Pharmacogenomics Platform (Santiago de Compostela, Spain). Ten mM stock solutions of the tested compounds were serially diluted, from 300 μM to 0.1 μM, in a 384-well UV-transparent plate (Greiner 781801) with 1%

DMSO : 99% PBS, incubated at 37 ºC for 2 h, and the light scattering was measured in a NEPHELOstar Plus reader (BMG LABTECH). The results were adjusted to a segmented linear regression to obtain the maximum concentration in which the compounds were soluble.

Cytotoxicity in SH-SY5Y cells. The cytotoxicity of the target compounds was evaluated in human neuroblastoma SH-SY5Y cells (ATCC Number: CRL-2266). Cells were cultured in Minimum Essential Medium / Ham's-F12 (1:1, v/v) medium (Biowest), supplemented with non-essential amino acids, 10% fetal bovine serum, 1 mM glutamine and 50 µg mL^{-1} gentamycin (all reagents from Gibco, Thermo Fisher Scientific). For the experiments, cells were seeded at 3×10^5 cells mL^{-1} (100 µL per well) in 96-well plates (Nunc). After 24 h, the test compounds were added concentrated from stock solutions in DMSO and culture medium to triplicate wells to obtain the final different concentrations up to 100 µM in 0.1% DMSO. Compounds were incubated for further 24 h. At termination, cytotoxicity was analyzed by the propidium iodide (PI) fluorescence stain assay. All compounds were tested in three independent experiments using different cell passages. The PI assay measures cell death. PI enters into the cells with damaged membranes and greatly increases the fluorescence upon binding to DNA. PI reagent (Molecular Probes) at the final concentration of 7.5 µg mL^{-1} was added to the cells and incubated for 1 h. The resulting fluorescence was measured by a Flx-Xenius microplate reader (SAFAS) at λ_{ex} = 530 nm, λ_{em} = 645 nm. The percentage of cell death induced by the compounds was calculated from to the fluorescence of treated cells (Ft) relative to that of control cells (Fmin) and cells incubated with Triton X100 (Fmax) as the 0% and 100% cell death, respectively [% = ((Ft-Fmin)/(Fmax-Fmin)) × 100]. All compounds were tested in three independent experiments using different cell passages. LC_{50} values were calculated for each experiment and the results are expressed as mean ± SD.

Microsomal stability screen. The microsomal stability of the target compounds was determined at the Innopharma Drug Screening and Pharmacogenomics Platform (Santiago de

Compostela, Spain). The human, rat and mice pooled microsomes, with a protein content of 20 mg mL^{-1}, were purchased from Tebu-Xenotech. The compounds were incubated in a 96-well microplate at 37 ºC with the microsomes in 50 mM phosphate buffer (pH = 7.4) containing as cofactors 30 mM $MgCl_2$, 10 mM NADP, 100 mM glucose-6-phosphate and 40 U mL^{-1} glucose-6-phosphate dehydrogenase, using the volumes indicated in Table S3. Samples (75 µL) were taken from each well at 0, 10, 20, 40 and 60 min and transferred to a microplate. 4 °C Acetonitrile (75 µL) and internal standard (rolipram) were then added for inactivating the microsomes, and water with 0.5% formic acid (30 µL) was subsequently added for improving the chromatographic conditions, keeping the mixtures at 4 °C. The plate was centrifuged at 46,000 g for 30 min at 15 ºC and supernatants were taken and analyzed in a UPLC-MS/MS system (UPLC QSM Waters Acquity) using reverse phase Acquity UPLC® HSST3 1.7 µm (2.1 mm × 50 mm, Waters) as the stationary phase, 0.1% formic acid in water (A) / 0.1% formic acid in acetonitrile (B) as the mobile phase, a flow of 0.8 mL min^{-1}, and the gradient indicated in Table S4. The metabolic stability of the compounds was calculated from the logarithm of the remaining compound at each of the time points studied.

In Vivo Proof-of-Concept of the Dual sEH / AChE Inhibitor 12b in SAMP8 Mice

Animals and treatment. The senescence-accelerated mouse-prone 8 (SAMP8) is an accelerated aging model established through phenotypic selection from AKR/J mice by Takeda in early 1979.[13] This murine model shows histopathological features of AD, such as abnormal APP and β-amyloid processing and tau pathology, among others. Likewise, SAMP8 exhibits cognitive and emotional abnormalities from young ages.[13,14] Moreover, inflammatory and oxidative stress markers are present at early ages and during adultness.[15] In this work, 5-month-old male SAMP8 mice were used to perform behavioral and molecular analyses. Animals were randomly divided into SAMP8 control (n=8) and SAMP8 treated with **12c**

(n=8, 2 mg kg^{-1} day^{-1}). The animals had free access to food and water and were kept under standard temperature conditions (22 ± 2 ºC) and 12-h/12-h light/dark cycles (300 lux/0 lux). Compound **12c** was dissolved in 1.8% (2-hydroxypropyl)-β-cyclodextrin (Sigma-Aldrich, St. Louis, MO) and administered through drinking water for 4 weeks (Figure S6 of the Supporting Information). Control groups received water plus 1.8% (2-hydroxypropyl)-β-cyclodextrin during the treatment period. After 4 weeks of treatment, behavioral and cognitive tests were performed to study the effects of treatment on working memory. Drug administration was continued until euthanasia by cervical dislocation, three days after the cognitive tests were completed. The brains were immediately removed from the skulls and the hippocampi were dissected, frozen and maintained at –80 ºC.

Weight and water consumption were controlled each week, and compound concentration was adjusted accordingly to reach the optimal dose. Mice were treated according to European Community Council Directive 86/609/EEC and were approved by the Institutional Animal Care and Use Committee of the University of Barcelona (670/14/8102) and by Generalitat de Catalunya, Spain (10291). All studies and procedures for the behavioral tests, brain dissection and extractions followed the ARRIVE. Every effort was made to minimize animal suffering and to reduce the number of animals.

Behavioural tests. Novel Object Recognition Test (NORT). The NORT protocol was performed.[16] Briefly, mice were placed in a 90º two-arm (25 × 20 × 5 cm) black maze, with removable walls for easy cleaning and light intensity in mid-field was 30 lux. Before the memory trials, mice were habituated to the apparatus for 10 min for 3 days. On day 4, the animals were subjected to a 10 min acquisition trial, in which they were allowed to freely explore two identical objects located at the end of each arm (First trial-Familiarization). After 2 h (short-term memory determination) and 24 h (long-term memory determination) from the first trial, mice were subjected to a 10 min retention trial, in which one of the two old objects

had been replaced by a novel one. The behavior was recorded, and the time that the mice spent exploring the new object (TN) and the old one (TO) were measured manually. Exploration was defined as sniffing or touching the objects with the nose and/or forepaws. The discrimination index (DI) was calculated as (TN–TO)/(TN+TO). To avoid object preference biases, objects were alternated. 70% EtOH was used to clean the arms and objects after each trial for the elimination of olfactory cues.

Molecular analyses. Immunodetection experiments by western blot. The hippocampal tissue from each animal was homogenized in lysis buffer (Tris HCl pH 7.4 mM, NaCl 150 mM, EDTA 5 mM and 1X-Triton X-100) containing phosphatase and protease inhibitors (Cocktail II, Sigma-Aldrich, St. Louis, MO, USA) to obtain total protein homogenates. The Bradford technique was used to determine total protein concentration. Aliquots of 15 μg of total hippocampal protein were used and separated by sodium dodecyl sulfate-polyacrylamide gel electrolysis (SDS-PAGE) (8–20%) and transferred into polyvinylidene difluoride (PVDF) membranes (Merck-Millipore, Burlington, MA, USA) for 2 h. The membranes were blocked in 5% non-fat milk in TRIS-buffered saline (TBS) containing 0.1% Tween 20 (TBS-T, Sigma-Aldrich, St. Louis, MO, USA) for 1 h at room temperature, and then, overnight incubation at 4 ºC with the primary antibodies listed in Table S5 was carried out. The membranes were washed with TBS-T 3 times for 5 min and incubated with secondary antibodies for 1 h at room temperature. Immunoreactive protein was viewed with a Chemiluminescence-based detection kit, following the manufacturer’s protocol (ECL Kit; Merck-Millipore), and digital images were acquired using a ChemiDoc XRS+ System (BioRad lab, Hercules, CA, USA). Semi-quantitative analyses were done using ImageLab Software (BioRad Lab), and results were expressed in Arbitrary Units (AU) considering control protein levels as 100%. Protein loading was routinely monitored by immunodetection of glyceraldehyde-3-phosphate dehydrogenase (GADPH).

RNA extraction and gene expression determination by q-PCR. Total RNA isolation from hippocampal tissue was carried out using TRIsure™ reagent following the manufacturer's instructions (Bioline Reagents, London, UK). The yield, purity and quality of RNA were determined spectrophotometrically with a NanoDrop™ ND-1000 (ThermoFisher Scientific, Waltham, MA, USA) apparatus and an Agilent 2100B Bioanalyzer (Agilent Technologies, Santa Clara, CA, USA). RNAs with 260/280 ratios and RIN higher than 7.5, respectively, were selected. Reverse transcription-polymerase chain reaction (RT-PCR) was performed as follows: 2 µg of messenger RNA (mRNA) was reverse-transcribed using the high-capacity cDNA reverse transcription Kit (Applied Biosystems, Foster City, CA, USA). Real-time quantitative PCR (qPCR) was used to quantify mRNA expression genes listed in Table S6. Real-time PCR was performed by using Step One Plus Detection System (Applied-Biosystems, Waltham, MA, USA) employing SYBR® Green PCR Master Mix (Applied-Biosystems). Each reaction mixture contained 6.75 µL of complementary DNA (cDNA) (2 µg μL^{-1}), 0.75 µL of each primer (100 nM), and 6.75 µL of SYBR® Green PCR Master Mix (2X) (Applied Biosystems). Data were analyzed utilizing the comparative Cycle threshold (Ct) method (ΔΔCt), where the housekeeping gene level was used to normalize differences in sample loading and preparation. Normalization of expression levels was performed with β-actin for SYBR® Green-based real-time PCR results. Each sample was analyzed in triplicate and the results represent the n-fold difference of the transcript levels among groups.

Statistical analysis in in vivo efficacy studies. All data are expressed as the mean ± standard error of the mean (SEM). Statistical analysis was conducted using GraphPad Prism version 8 statistical software and statistical significance was considered when p values were <0.05. All data were tested for normal distribution and equal variance. Group size may vary according to power analysis and expertise of the authors regarding the behavioral tests.[15]

Blind analysis was performed for behavioral tests. Statistical outliers were determined with Grubbs' test and when necessary were removed.

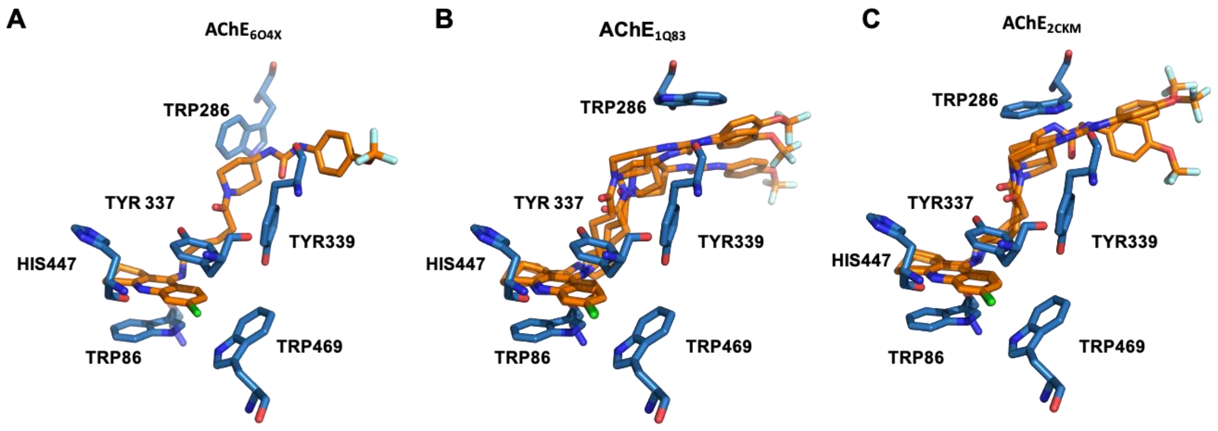

Figure S1. Best scoring poses for **12c** in each of the AChE models evaluated. Residues of the catalytic anionic site were modelled as in the crystallographic structure with PDB ID 6O4X, while the rotamer of Trp286 was adjusted to reproduce its arrangement in PDB ID 6O4X (A), PDB ID 1Q83 (B) and PDB ID 2CKM (C).

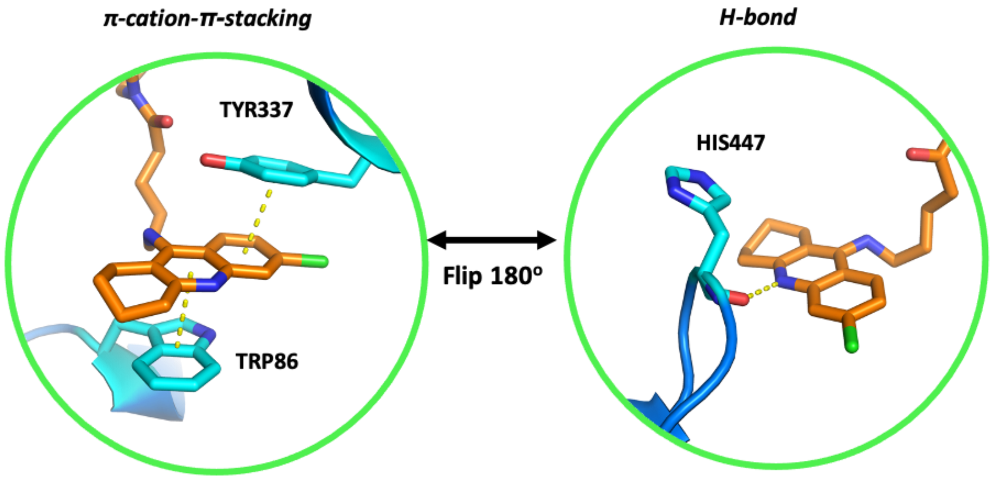

Figure S2. Interaction patterns of the 6-chlorotacrine moiety of **12c** in the CAS of AChE.

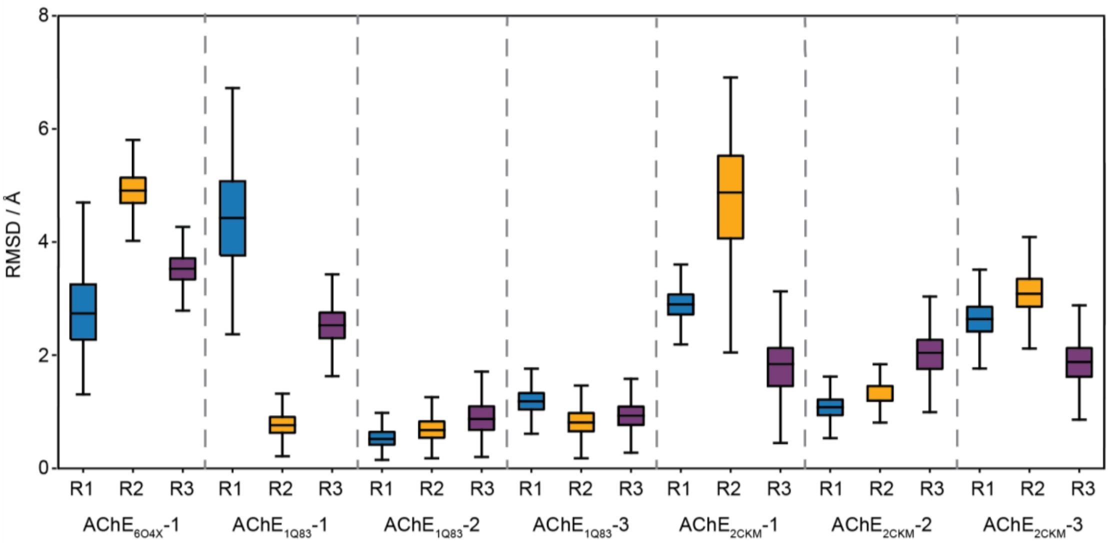

Figure S3. RMSD value distribution of the 6-chlorotacrine moiety of **12c** over the last 100 ns of each simulation. The starting structure of each system was used as reference to calculate the RMSD.

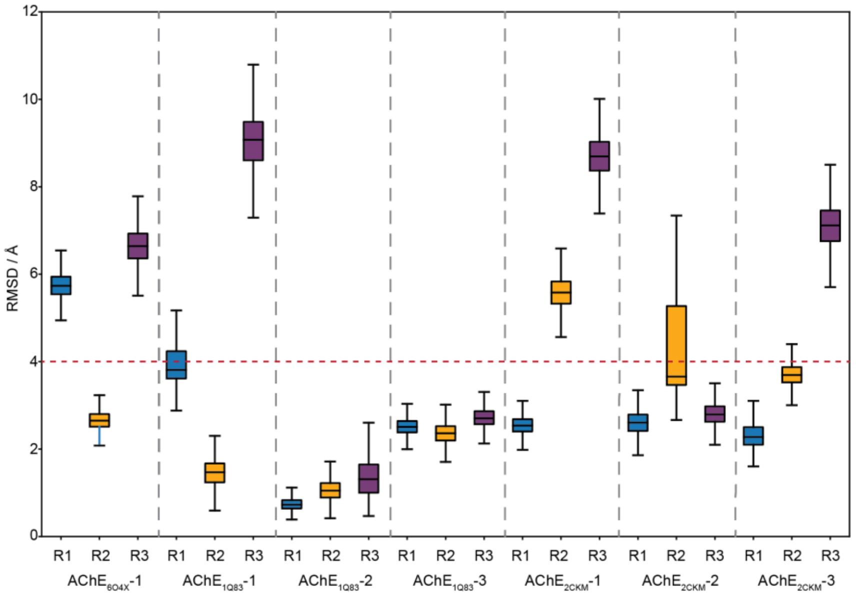

Figure S4. RMSD values of the TPPU moiety of **12c** over the last 100 ns of each simulation. The reference structure was taken as the average structure over the last 100 ns of the $AChE_{1Q83}$-2 system (Supplementary Structure S1). The red discontinuous line demarks the threshold to consider whether simulations converged to the same binding mode (4 Å / 0.2 Å per heavy atom on the TPPU moiety).

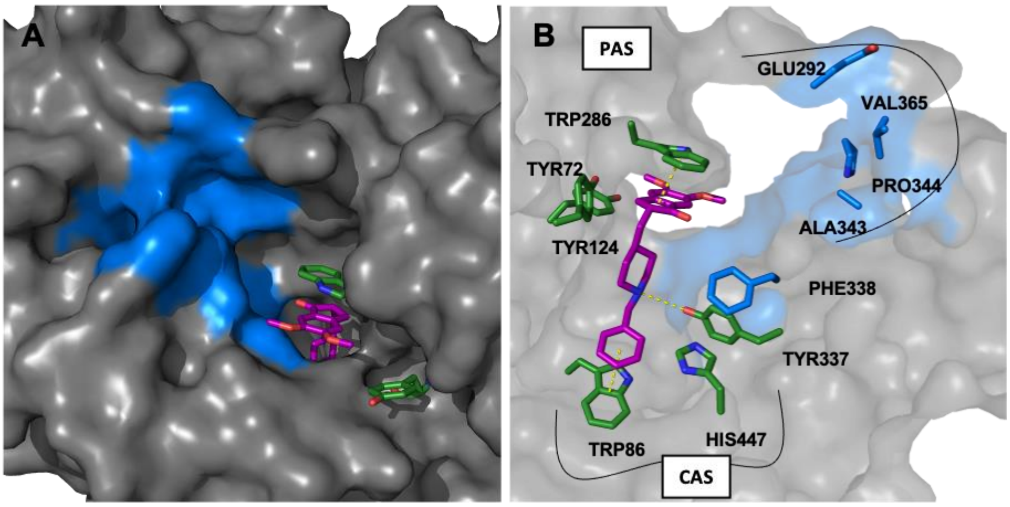

Figure S5. Binding mode of donepezil in control simulations. The cryptic pocket in the PAS did not open in none of the trajectories of the AChE–donepezil complex (A) and the ligand retained the pattern of interactions observed in crystallographic structures (B).

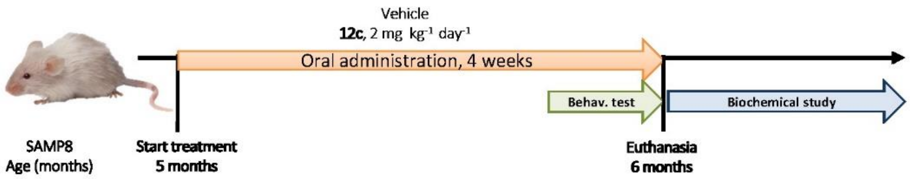

Figure S6. *In vivo* treatment and experimental timeline.

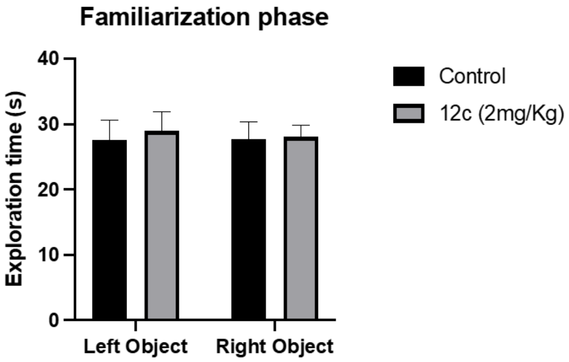

Figure S7. Familiarization phase of the NORT test.

Table S1. Summary of the AChE Structures Available in the PDB that Were Analyzed to Identify Prior Knowledge of the Cryptic Pocket in the PAS.

**Human *(Homo sapiens)***							
1b41	4ey6	5hf5	6cqu	6nea	6nto	6o5v	6wuy
1f8u	4ey7	5hf6	6cqv	6ntg	6o4w	6o66	6wuz
2x8b	4ey8	5hf8	6cqw	6nth	6o4x	6o69	6wv1
3lii	4m0e	5hf9	6cqz	6ntk	6o50	6u34	6wvc
4bdt	4m0f	5hfa	6cqy	6ntl	6o52	6u37	6wvo
4ey4	4pqe	5hq3	6cqz	6ntm	6o5r	6u3p	6wvp
4ey5	5fpq	6cqt	6f25	6ntn	6o5s	6wuv	6wvq
**Murine (*Mus musculus*)**							
1j06	2gyu	2jey	2whq	2xuk	4a16	4bc0	5eih
1j07	2gyv	2jez	2whr	2xuo	4a23	4bc1	5fkj
1ku6	2gyw	2jf0	2wls	2xup	4ab7z	4btl	5foq
1maa	2h9y	2jge	2wu3	2xuq	4ara	4qww	5fpp
1mah	2ha0	2jgf	2wu4	2y2u	4arb	5dti	5fum
1n5m	2ha2	2jgi	2xud	2y2v	4b80	5dtj	5hcu
1n5r	2ha3	2jgj	2xuf	3dl4	4b81	5ehn	5ov9
1q83	2ha4	2jgk	2xug	3dl7	4b82	5ehq	6fsd
1q84	2ha5	2jgl	2xuh	3zlt	4b83	5ehz	6fse
2c0p	2ha6	2jgm	2xui	3zlu	4b84	5eia	6td2
2c0q	2ha7	2whp	2xuj	3zlv	4b85	5eie	
**Pacific electric ray (*Torpedo californica)***							
1acj	1gqs	1qim	2ack	2vja	3i6z	5nap	6g4n
1acl	1h22	1qti	2bag	2vjb	3m3	5nau	6g4o
1amn	1hbj	1som	2c4h	2vjc	3zv7	5nuu	6g4p
1ax9	1jjb	1ut6	2c58	2vjd	4tvk	6euc	6h12
1cfj	1oce	1vot	2c5g	2vq6	4w63	6eue	6h13
1dx6	1odc	1vxo	2cek	2vt7	4x3c	6ewk	6h14
1e3q	1qid	1vxr	2ckm	2w6	5bwc	6ezg	6tt0
1e66	1qie	1w4l	2cmf	2wfz	5dlp	6fld	7b2w
1ea5	1qif	1w6r	2dfp	2wg0	5e2i	6fqn	7b8e
1eve	1qig	1w75	2j3d	2wg1	5e4j	6g17	
1fss	1qih	1w76	2j3q	2wg2	5e4t	6g1u	
1gpk	1qii	1zgb	2j4f	2xi4	5ehx	6g1v	
1gpn	1qij	1zgc	2v96	3gel	5ei5	6g1w	
1gqr	1qik	2ace	2va9	3i6m	5ih7	6g4m	
**Electric eel (*Electrophorus electricus*)**							
1c2b	1c2o	1eea					

Table S2. Reported and Experimental Permeability (*Pe* 10^{-6} cm s^{-1}) Values in the PAMPA-BBB Assay of the Commercial Drugs Used for Assay Validation.

Compound	Bibliography value[12]	Experimental value (n=3) ± S.D.
Cimetidine	0.0	0.7 ± 0.1
Lomefloxacin	1.1	0.8 ± 0.1
Norfloxacin	0.1	0.9 ± 0.1
Ofloxacin	0.8	1.2 ± 0.1
Hydrocortisone	1.9	1.4 ± 0.1
Piroxicam	2.5	2.3 ± 0.1
Clonidine	5.3	6.5 ± 0.1
Corticosterone	5.1	6.7 ± 0.1
Imipramine	13.0	12.3 ± 0.1
Promazine	8.8	13.8 ± 0.3
Progesterone	9.3	16.8 ± 0.3
Desipramine	12.0	17.8 ± 0.1
Testosterone	17.0	26.9 ± 0.6
Verapamil	16.0	28.5 ± 0.8

Table S3. Quantities of Reagents, Microsomes, and Test Compounds Used in the Microsomal Stability Assays.

		Blank (µL)	Human (µL)	Mouse (µL)	Rat (µL)
Phosphate buffer Na/K 50 mM pH 7.4		333	296	310	301
$MgCl_2$ 30 mM	cofactors	162.5	162.5	162.5	162.5
NADP 10 mM					
Glucose 6-P 100 mM					
Glucose 6-P DH 20 U mL^{-1}					
Human microsomes			36.5		
Mouse microsomes				22.4	
Rat microsomes					31.4
Test compounds 5 µM		5	5	5	5

Table S4. Gradient Used in the UPLC-MS/MS Analysis for Microsomal Stability Studies.

Time (min)	A (%)	B (%)
0	95	5
0.1	95	5
0.5	2	98
0.75	2	98
0.9	95	5
1	95	5

Table S5. Antibodies Used in Western Blot Studies.

Antibody (WB)	Host	Source/Catalog/RRID	WB dilution
NFkβ	Rabbit	Cell signaling (Danvers, MA, USA) D14E12	1:1000
p-Tau (Ser396)	Rabbit	Invitrogen (Waltham, MA, USA) 44752G	1:1000
p-Tau (Ser404)	Rabbit	Invitrogen/44758G	1:1000
sAPPα	Rabbit	Covance (Princeton, NJ, USA) SIG39139	1:500
sAPPβ	Rabbit	Covance/SIG-39138-050	1:500
SYN	Mouse	Merck-Millipore/MAB5258	1:1000
Total Tau	Mouse	Invitrogen/AHB0042	1:1000
GAPDH	Mouse	Millipore/MAB374	1:2500
Goat-anti-mouse HRP conjugated		Biorad Lab/170-5047	1:5000
Goat-anti-rabbit HRP conjugated		Biorad Lab/170-6515	1:5000

Table S6. Primers and Probes Used in qPCR Studies.

Target	Product size (bp)	Forward primer (5'-3')	Reverse primer (5'-3')
Il-1β	179	ACAGAATATCAACCAACAAGTGATATTCTC	GATTCTTTCCTTTGAGGCCCA
Gfap	125	CCTTCTGACACGGATTTGGT	ACATCGAGATCGCCACCTAC
Il-6	189	ATCCAGTTGCCTTCTTGGGACTGA	TAAGCCTCCGACTTGTGAAGTGGT
Actin	190	CAACGAGCGGTTCCGAT	GCCACAGGTTCCATACCCA

Table S7. Reference c_1 and c_2 values for Trp286 rotamers in AChE.

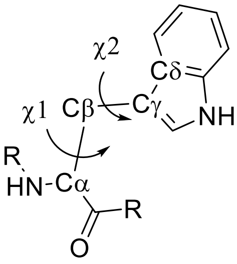

Reference PDB ID	6O4X	1Q83	2CKM
c_1(°)	–60	–120	–160
c_2(°)	–80	50	–120

References

(1) Shipps, G. W. Jr.; Cheng, C. C.; Herr, R. J.; Yang, J. Aliphatic Amines Based Heterocycles Useful as HIV Entry Blockers. WO2011/060396 A1, May, 19, 2011.

(2) Rose, T. E.; Morisseau, C.; Liu, J.-Y.; Inceoglu, B.; Jones, P. D.; Sanborn, J. R.; Hammock, B. D. 1-Aryl-3-(1-acylpiperidin-4-yl)urea Inhibitors of Human and Murine Soluble Epoxide Hydrolase: Structure−Activity Relationships, Pharmacokinetics, and Reduction of Inflammatory Pain. *J. Med. Chem.* **2010**, *53*, 7067–7075.

(3) Camps, P.; Formosa, X.; Galdeano, C.; Gómez, T.; Muñoz-Torrero, D.; Scarpellini, M.; Viayna, E.; Badia, A.; Clos, M. V.; Camins, A.; Pallàs, M.; Bartolini, M.; Mancini, F.; Andrisano, V.; Estelrich, J.; Lizondo, M.; Bidon-Chanal, A.; Luque, F. J. Novel Donepezil-Based Inhibitors of Acetyl- and Butyrylcholinesterase and Acetylcholinesterase-Induced β-Amyloid Aggregation. *J. Med. Chem.* **2008**, *51*, 3588–3598.

(4) Hu, M.-K.; Lu, C.-F. A Facile Synthesis of Bis-Tacrine Isosteres. *Tetrahedron Lett.* **2000**, *41*, 1815–1818.

(5) Pérez-Areales, F. J.; Turcu, A. L.; Barniol-Xicota, M.; Pont, C.; Pivetta, D.; Espargaró, A.; Bartolini, M.; De Simone, A.; Andrisano, V.; Pérez, B.; Sabate, R.; Sureda, F. X.; Vázquez, S.; Muñoz-Torrero, D. A Novel Class of Multitarget Anti-Alzheimer Benzohomoadamantane–chlorotacrine Hybrids Modulating Cholinesterases and Glutamate NMDA Receptors. *Eur. J. Med. Chem.* **2019**, *180*, 613–626.

(6) Wagner, K.; Inceoglu, B.; Dong, H.; Yang, J.; Hwang, S. H.; Jones, P.; Morisseau, C.; Hammock, B. D. Comparative Efficacy of 3 Soluble Epoxide Hydrolase Inhibitors in Rat Neuropathic and Inflammatory Pain Models. *Eur. J. Pharmacol.* **2013**, *700*, 93–101.

(7) Morisseau, C.; Hammock, B. D. Measurement of Soluble Epoxide Hydrolase (SEH) Activity. *Curr. Protoc. Toxicol.* **2007**, *33*, 4.23.1–4.23.18.

(8) Ellman, G. L.; Courtney, K. D.; Andres, V.; Featherstone, R. M. A New and Rapid Colorimetric Determination of Acetylcholinesterase Activity. *Biochem. Pharmacol.* **1961**, *7*, 88–95.

(9) Taylor, P.; Lwebuga-Mukasa, J.; Lappi, S.; Rademacher, J. Propidium – a Fluorescent Probe for a Peripheral Anionic Site on Acetylcholinesterase, *Mol. Pharmacol.* **1974**, *10*, 703–708.

(10) Taylor, P.; Lappi, S. Interaction of Fluorescence Probes with Acetylcholinesterase, the Site and Specificity of Propidium Binding, *Biochemistry* **1975**, *14*, 1989–1997.

(11) Nunes-Tavares, N.; Nery da Matta, A.; Batista e Silva, C.M.; Araújo, G.M.N.; Louro, S.R.W.; Hassón-Voloch, A. Inhibition of Acetylcholinesterase from *Electrophorus electricus* (L.) by Tricyclic Antidepressants, *Int. J. Biochem. Cell Biol.* **2002**, *34*, 1071–1079.

(12) Di, L.; Kerns, E. H.; Fan, K.; McConnell, O. J.; Carter, G. T. High Throughput Artificial Membrane Permeability Assay for Blood–Brain Barrier. *Eur. J. Med. Chem.* **2003**, *38*, 223–232.

(13) Takeda, T. Senescence-Accelerated Mouse (SAM) with Special References to Neurodegeneration Models, SAMP8 and SAMP10 Mice. *Neurochem. Res.* **2009**, *34*, 639–659.

(14) Morley, J. E.; Armbrecht, H. J.; Farr, S. A.; Kumar, V. B. The Senescence Accelerated Mouse (SAMP8) as a Model for Oxidative Stress and Alzheimer's Disease. *Biochim. Biophys. Acta* **2012**, *1822*, 650–656.

(15) Griñán-Ferré, C.; Corpas, R.; Puigoriol-Illamola, D.; Palomera-Ávalos, V.; Sanfeliu, C.; Pallàs, M. Understanding Epigenetics in the Neurodegeneration of Alzheimer's Disease: SAMP8 Mouse Model. *J. Alzheimers Dis.* **2018**, *62*, 943–963.

(16) Companys-Alemany, J.; Turcu, A. L.; Bellver-Sanchis, A.; Loza, M. I.; Brea, J. M.; Canudas, A. M.; Leiva, R.; Vázquez, S.; Pallàs, M.; Griñán-Ferré, C. A Novel NMDA Receptor Antagonist Protects against Cognitive Decline Presented by Senescent Mice. *Pharmaceutics* **2020**, *12*, 284.

Copies of ^{1}H NMR (400 MHz, CD_3OD) and ^{13}C NMR (100.6 MHz, CD_3OD) spectra and HPLC traces of the target compounds

1-{1-{3-[(6-Chloro-1,2,3,4-tetrahydroacridin-9-yl)amino]propanoyl}piperidin-4-yl}-3-[4-(trifluoromethoxy)phenyl]urea (12a)

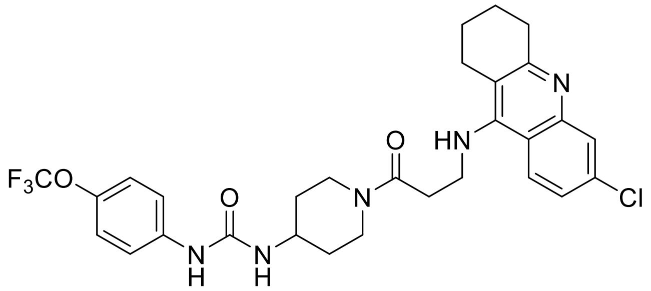

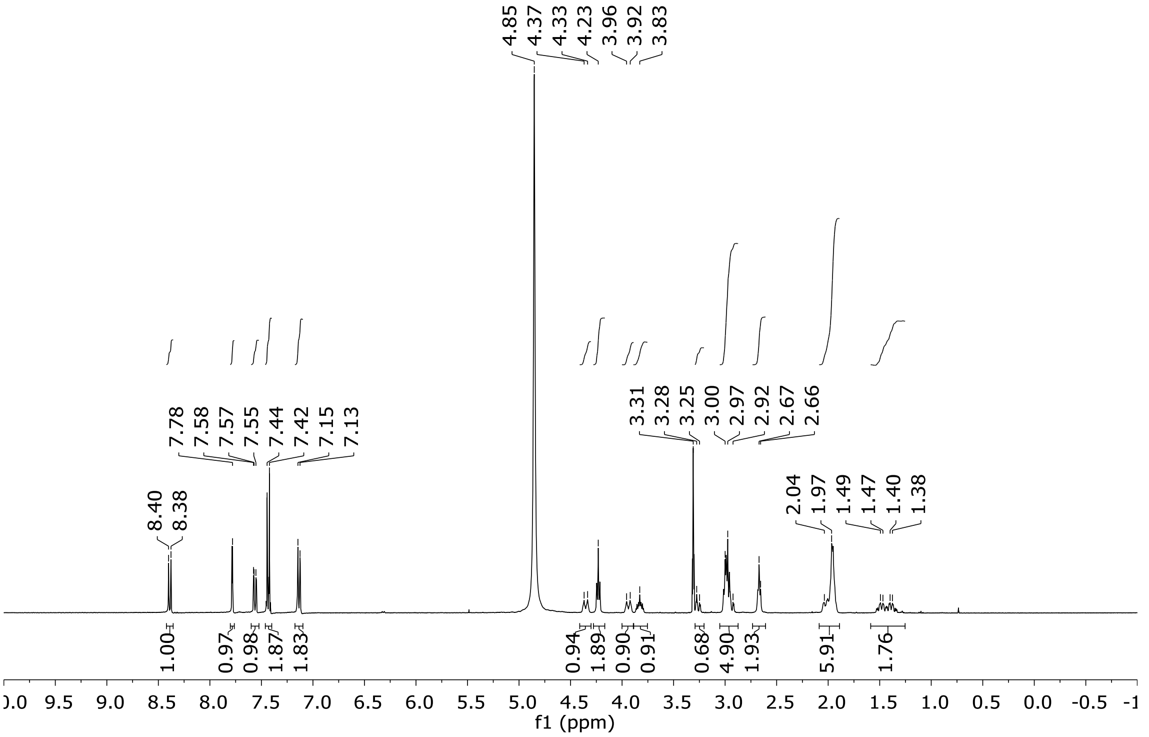

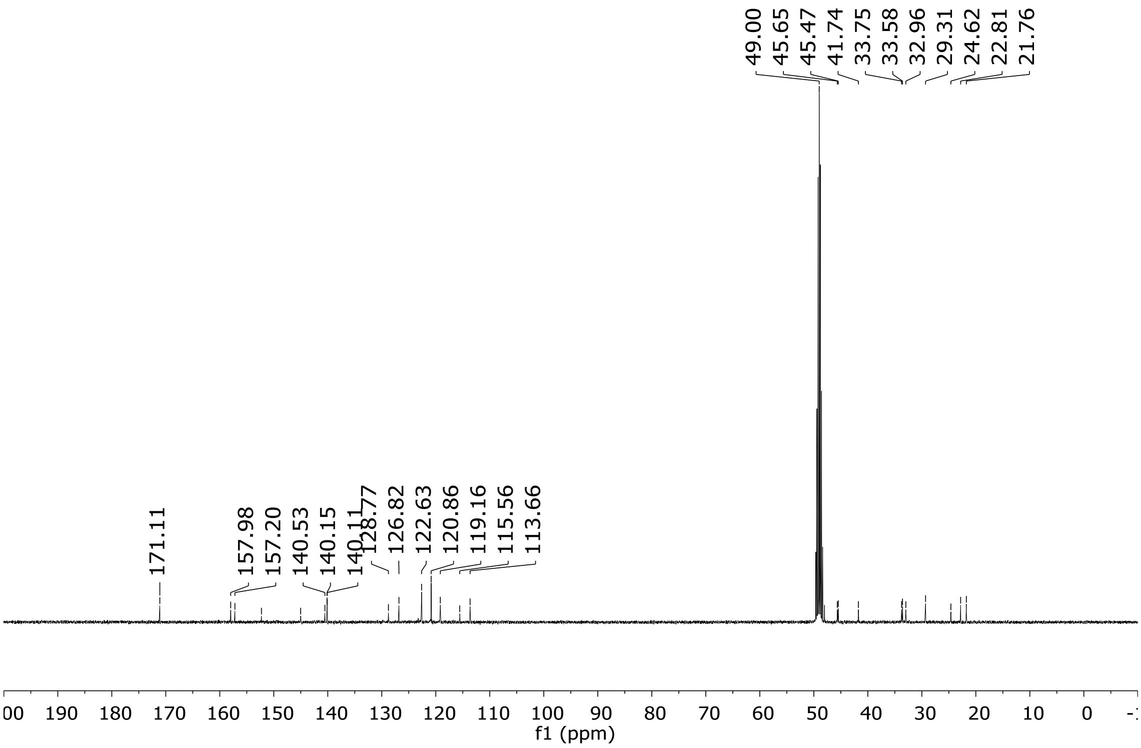
171.11
157.98
157.20
140.53
140.15
140.11
128.77
126.82
122.63
120.86
119.16
115.56
113.66
49.00
45.65
45.47
41.74
33.75
33.58
32.96
29.31
24.62
22.81
21.76
f1 (ppm)

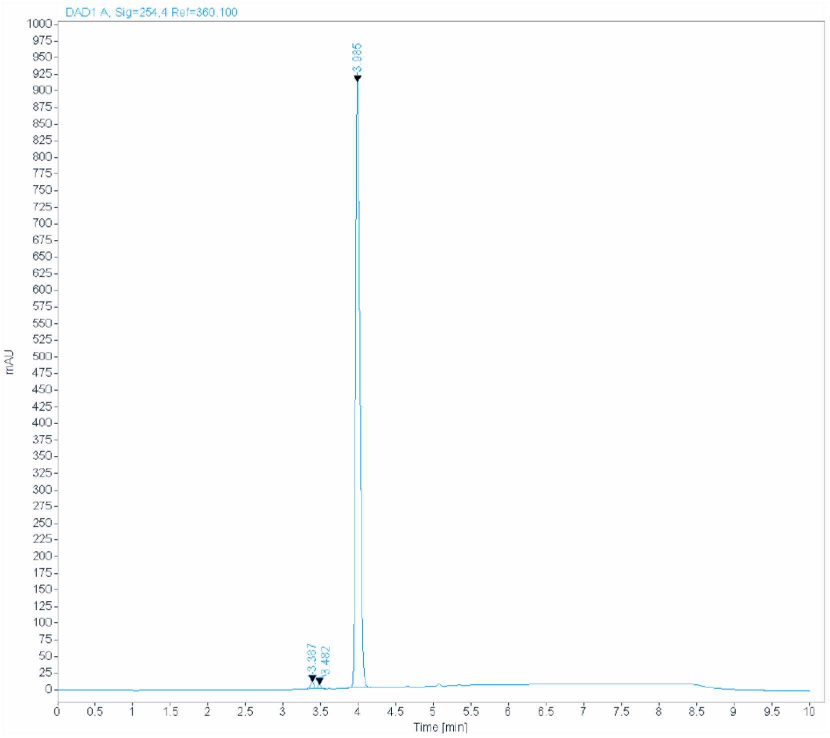
DAD1 A, Sig=254,4 Ref=360,100
3.985
3.387
3.482
mAU
Time [min]

1-{1-{4-[(6-Chloro-1,2,3,4-tetrahydroacridin-9-yl)amino]butanoyl}piperidin-4-yl}-3-[4-(trifluoromethoxy)phenyl]urea (12b)

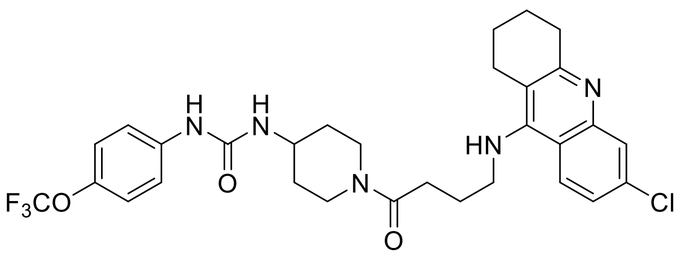

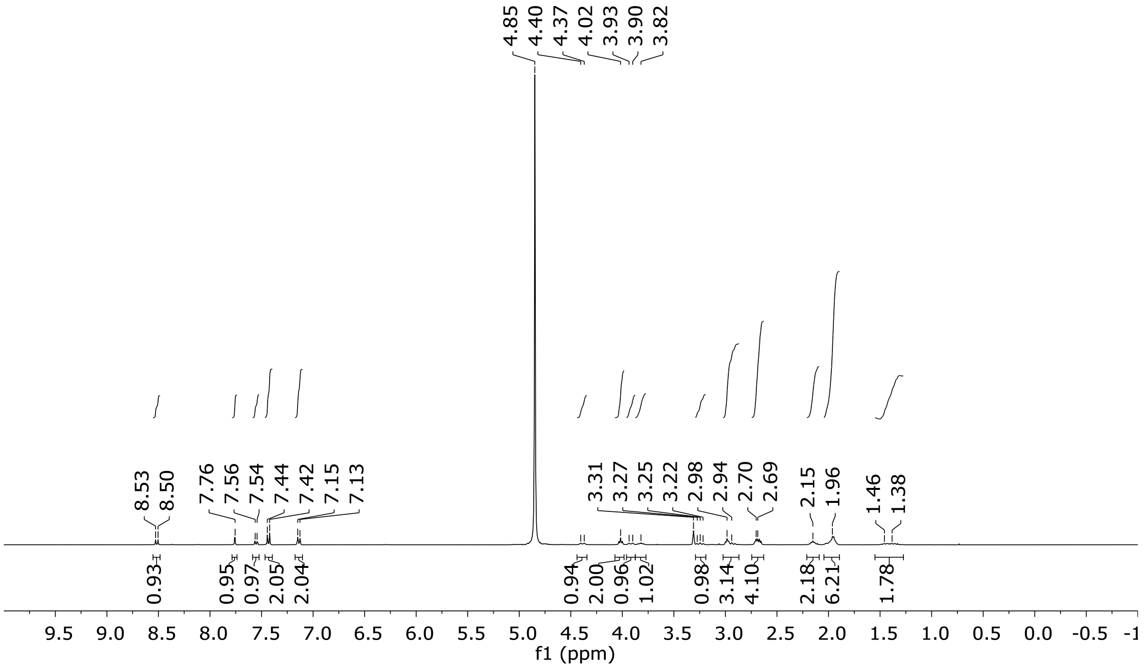

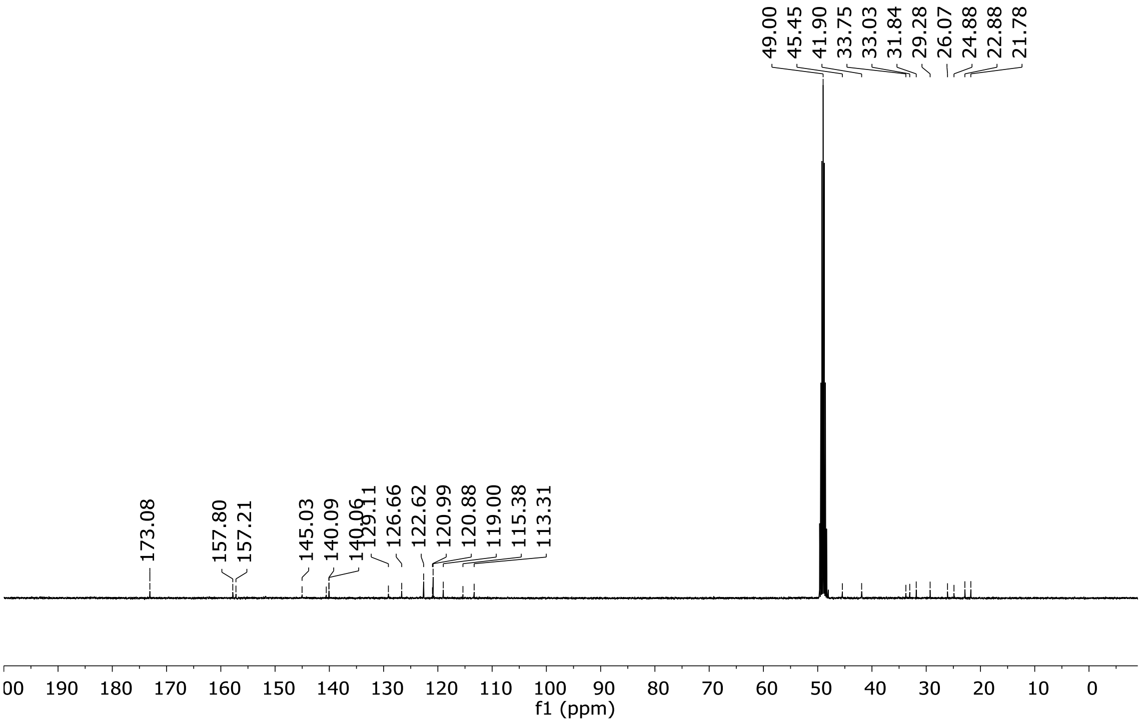
173.08
157.80
157.21
145.03
140.09
140.06
129.11
126.66
122.62
120.99
120.88
119.00
115.38
113.31
49.00
45.45
41.90
33.75
33.03
31.84
29.28
26.07
24.88
22.88
21.78
f1 (ppm)

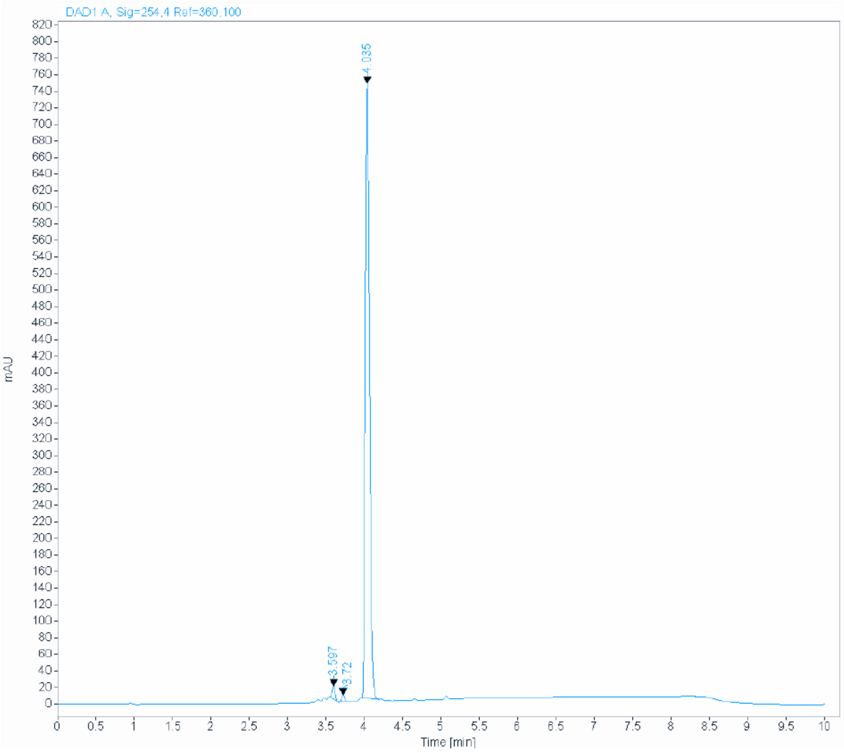
DAD1 A, Sig=254,4 Ref=360,100
mAU
4.035
3.597
3.72
Time [min]

1-{1-{5-[(6-Chloro-1,2,3,4-tetrahydroacridin-9-yl)amino]pentanoyl}piperidin-4-yl}-3-[4-(trifluoromethoxy)phenyl]urea (12c)

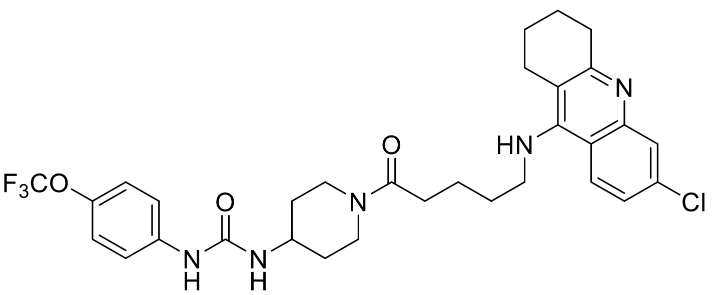

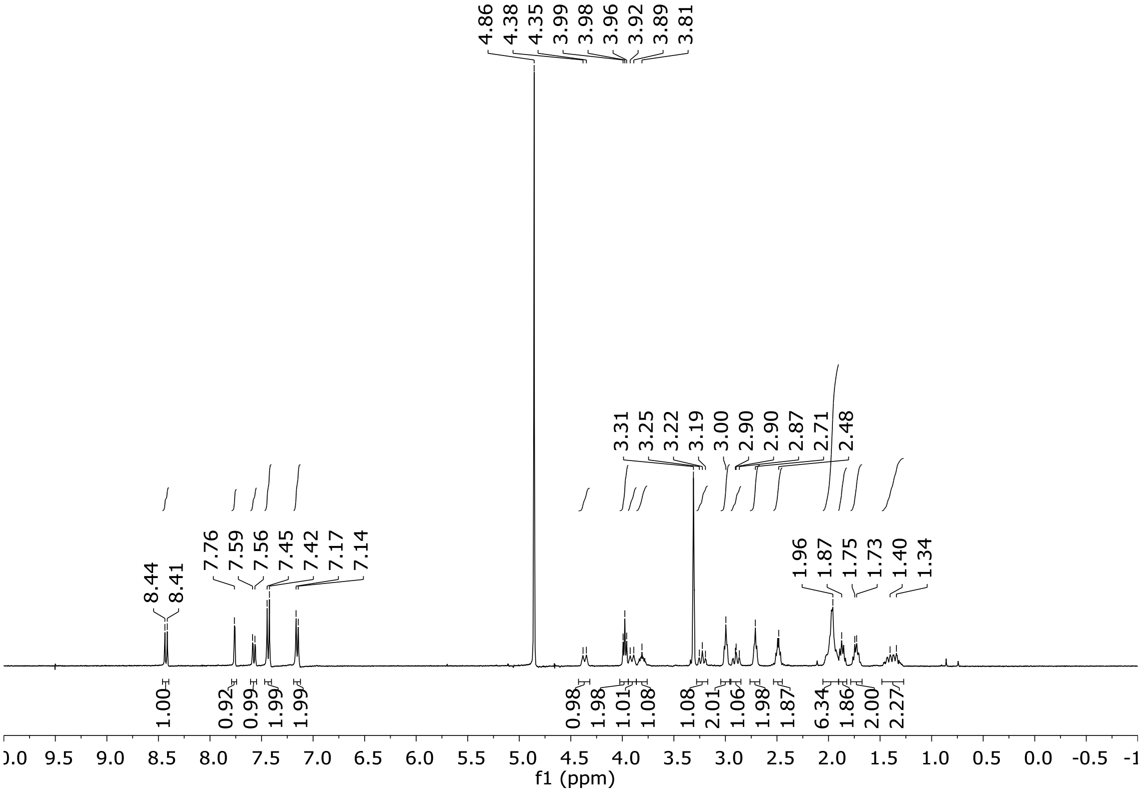

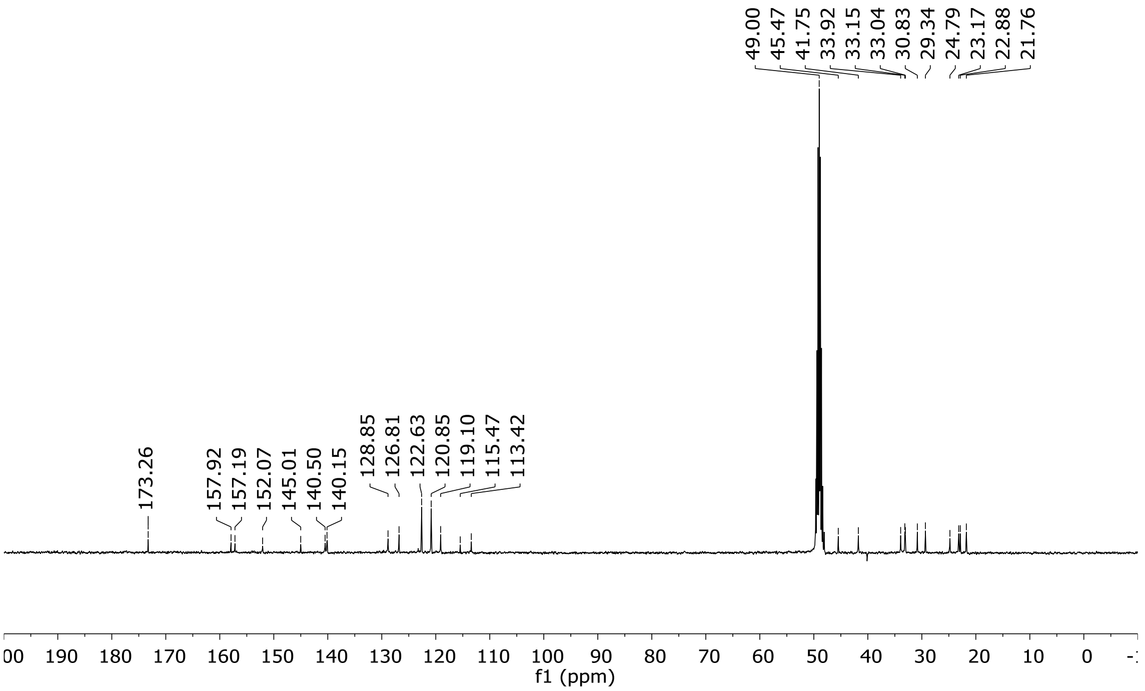

173.26
157.92
157.19
152.07
145.01
140.50
140.15
128.85
126.81
122.63
120.85
119.10
115.47
113.42
49.00
45.47
41.75
33.92
33.15
33.04
30.83
29.34
24.79
23.17
22.88
21.76
f1 (ppm)

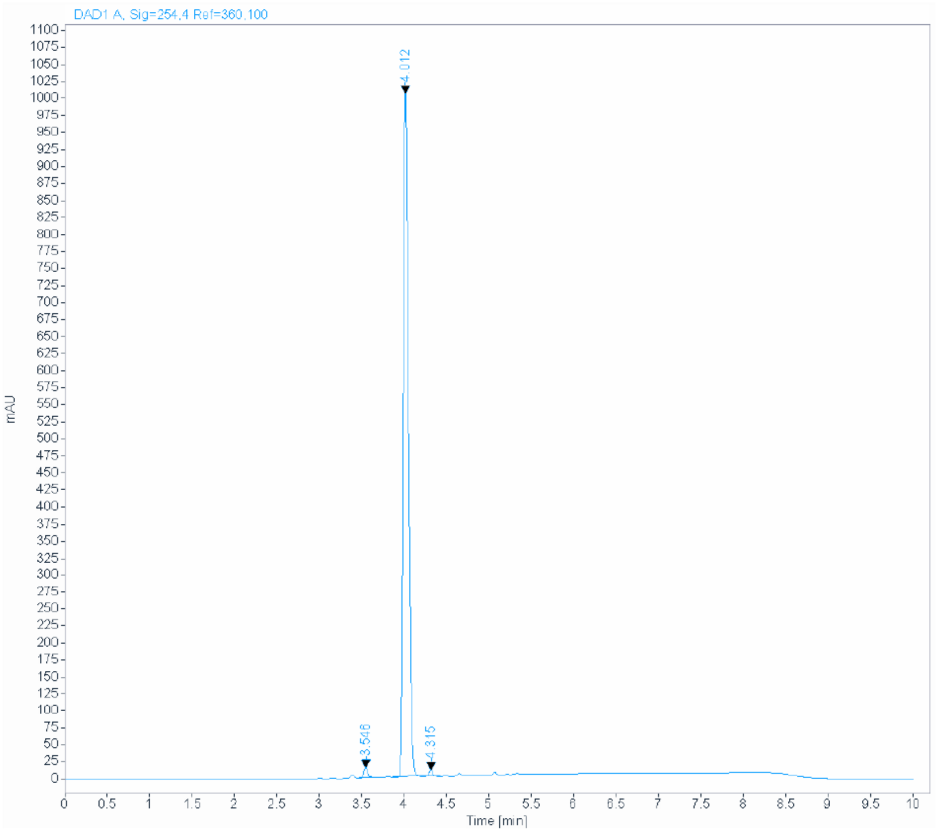

DAD1 A, Sig=254,4 Ref=360,100
4.012
3.546
4.315
mAU
Time [min]

(–)-(7*S*,11*S*)-1-{1-{5-[(3-Chloro-6,7,10,11-tetrahydro-9-methyl-7,11-methanocycloocta[*b*]quinolin-12-yl)amino]pentanoyl}piperidin-4-yl}-3-[4-(trifluoromethoxy)phenyl]urea [(–)-(7*S*,11*S*)-15]

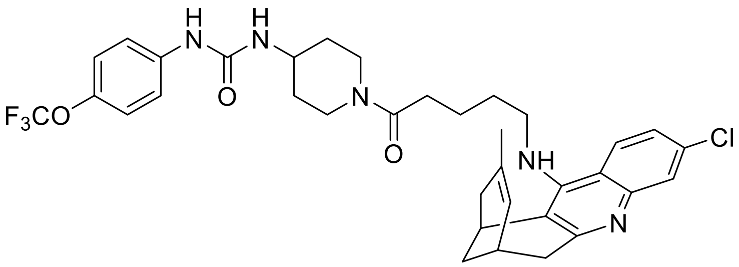

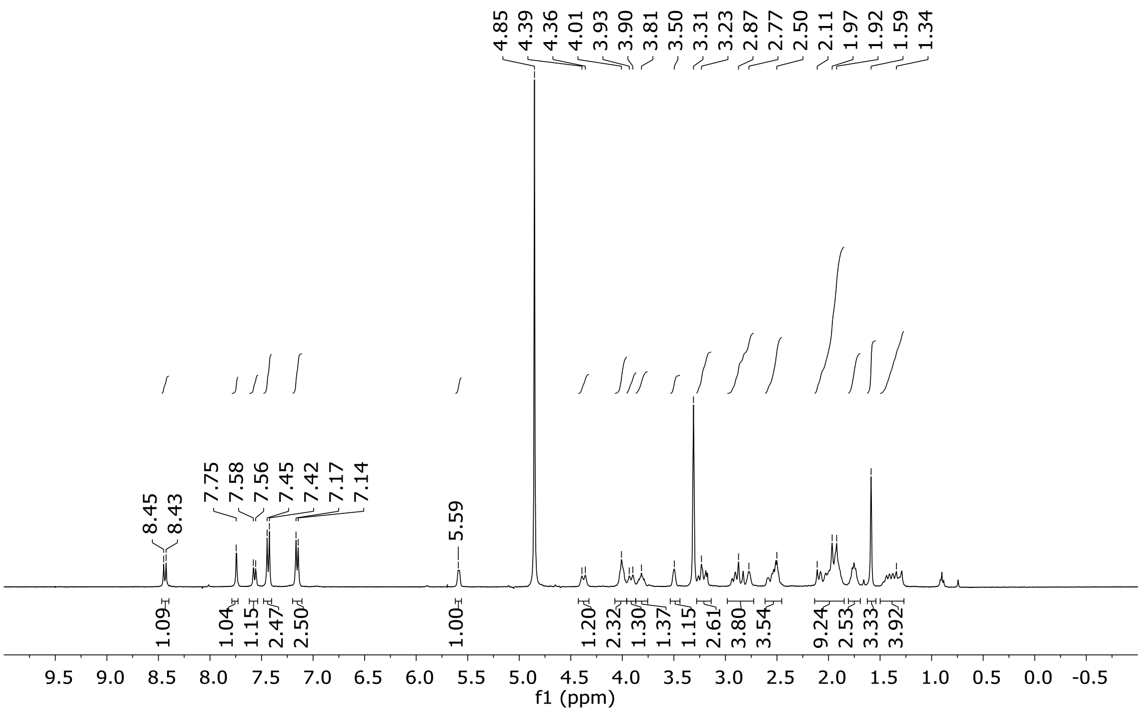

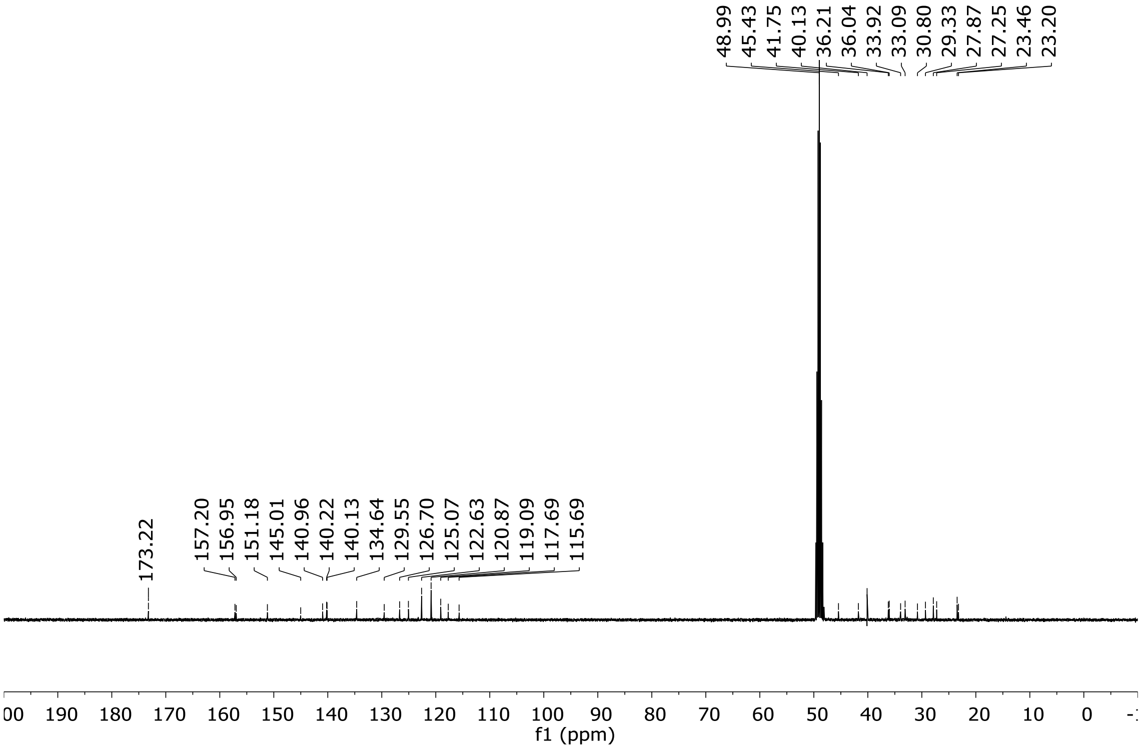
48.99
45.43
41.75
40.13
36.21
36.04
33.92
33.09
30.80
29.33
27.87
27.25
23.46
23.20
173.22
157.20
156.95
151.18
145.01
140.96
140.22
140.13
134.64
129.55
126.70
125.07
122.63
120.87
119.09
117.69
115.69
f1 (ppm)

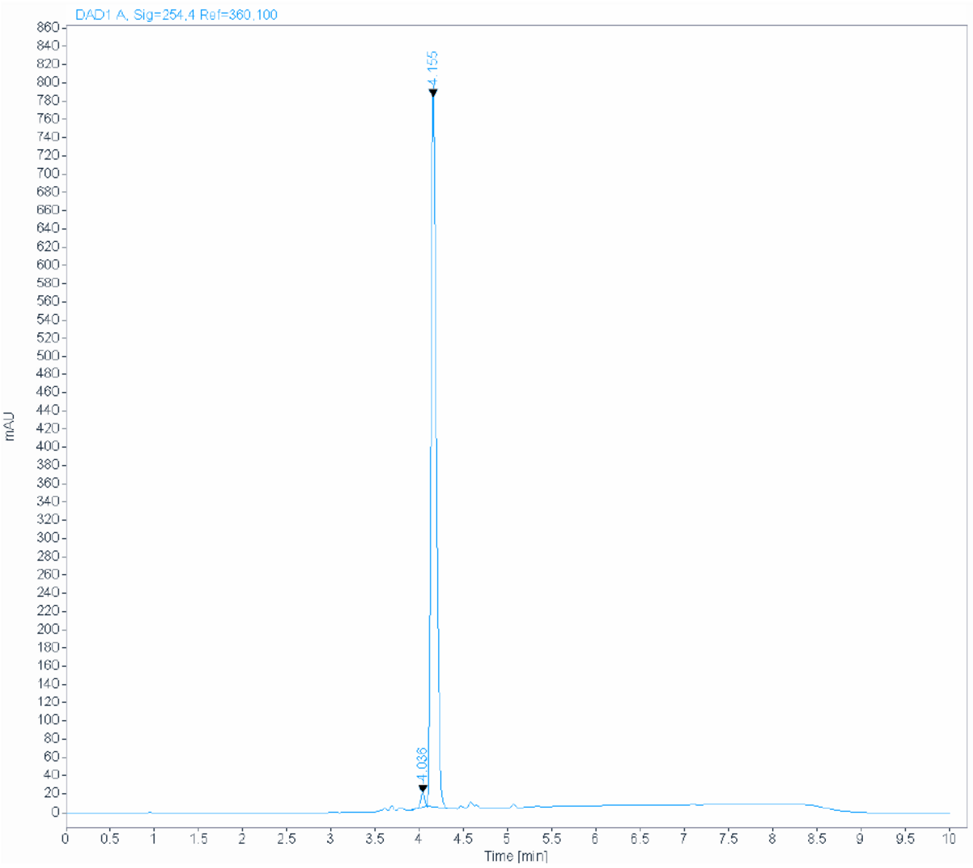
DAD1 A, Sig=254,4 Ref=360,100
4.155
4.036
mAU
Time [min]

(+)-(7*R*,11*R*)-1-{1-{5-[(3-Chloro-6,7,10,11-tetrahydro-9-methyl-7,11-methanocycloocta[*b*]quinolin-12-yl)amino]pentanoyl}piperidin-4-yl}-3-[4-(trifluoromethoxy)phenyl]urea [(+)-(7*R*,11*R*)-15]

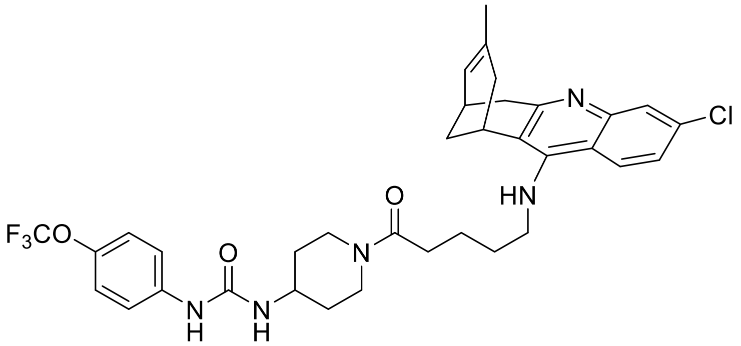

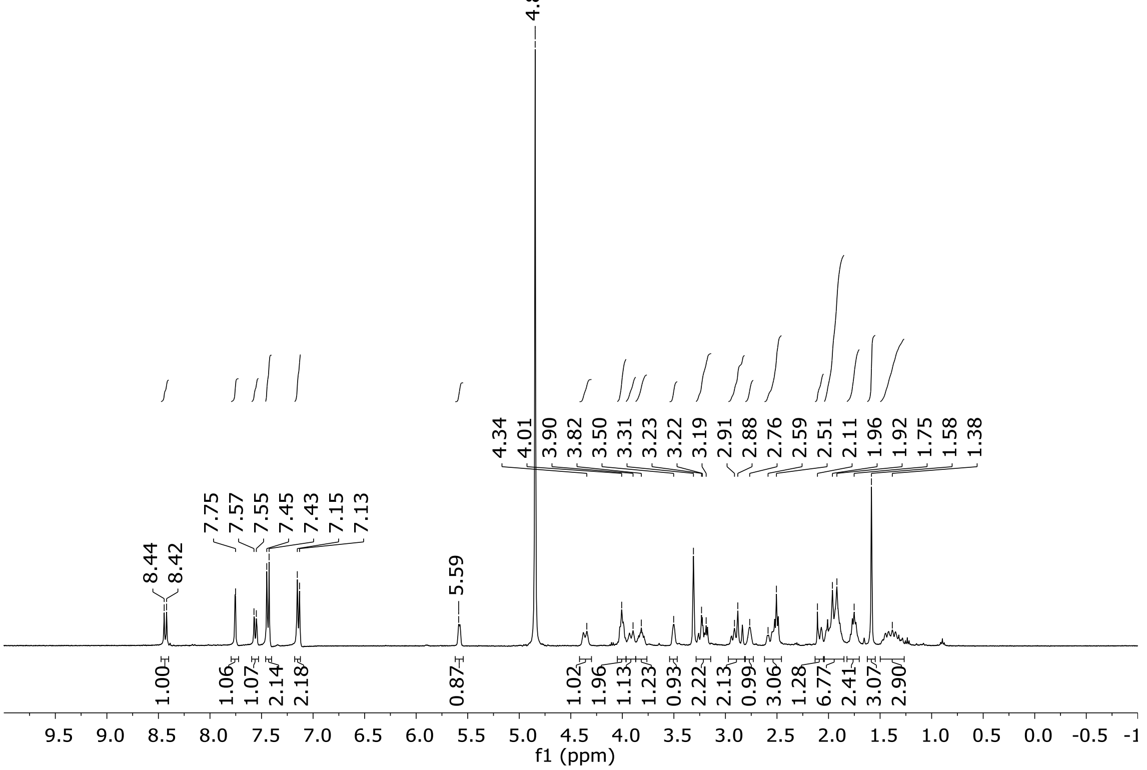

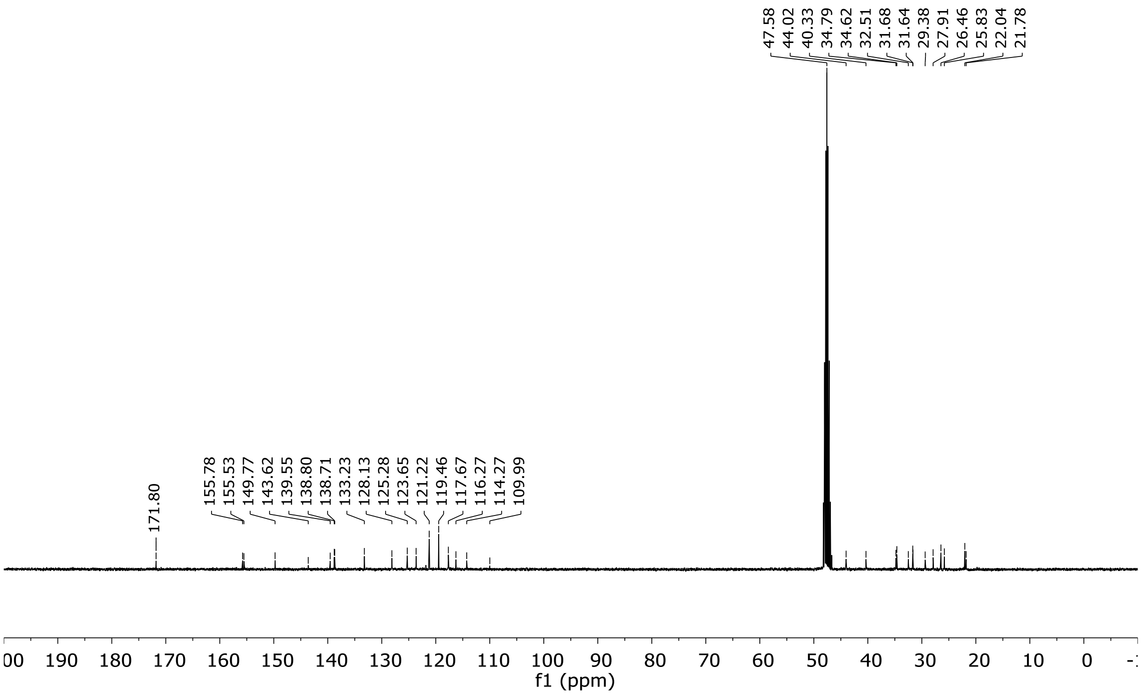

47.58
44.02
40.33
34.79
34.62
32.51
31.68
31.64
29.38
27.91
26.46
25.83
22.04
21.78
171.80
155.78
155.53
149.77
143.62
139.55
138.80
138.71
133.23
128.13
125.28
123.65
121.22
119.46
117.67
116.27
114.27
109.99
f1 (ppm)

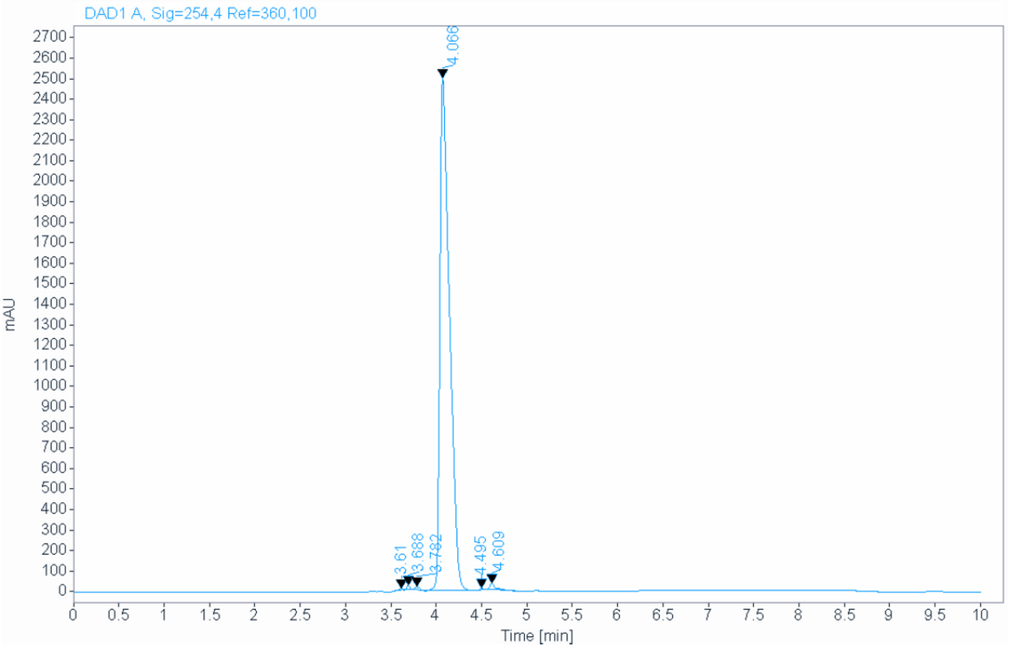

DAD1 A, Sig=254,4 Ref=360,100
mAU
Time [min]
4.066
3.61
3.688
3.782
4.495
4.609
